# Uncovering the molecular landscape of young-onset diffuse gastric cancer: A relieff-based feature selection analysis on RNA-Seq data

**DOI:** 10.1371/journal.pone.0354961

**Published:** 2026-08-06

**Authors:** Zahra Khalili Azni, Matia Sadat Borhani, Hossein Sabouri, Sayed Javad Sajadi, Maryam Pasandideh Arjmand

**Affiliations:** 1 Department of Biology, Faculty of Basic Sciences and Engineering, Gonbad Kavous University, Gonbad Kavous, Golestan, Iran; 2 Department of Plant Production, Faculty of Agriculture Sciences and Natural Resources, Gonbad Kavous University, Gonbad Kavous, Golestan, Iran; 3 Department of Plant Biotechnology, Faculty of Agricultural Sciences, University of Guilan, Rasht, Guilan, Iran; 4 Researcher, BioGenTAC lnc., Technology Incubator of Agricultural Biotechnology Research Institute of Iran-North Branch (ABRII), Rasht, Guilan, Iran; Xiangya Hospital Central South University, CHINA

## Abstract

**Background:**

Diffuse Gastric Cancer (DGC) is an aggressive subtype with a poor prognosis and a lack of specific biomarkers, representing a critical unmet need in oncology. This study aimed to elucidate the key molecular drivers of DGC by integrating RNA-seq data with a multi-faceted bioinformatics approach.

**Methods:**

We analyzed RNA-seq data from young-onset DGC and normal tissues (GSE113255, GSE122401). Machine learning (ML) feature selection (ReliefF algorithm) was used to prioritize genes, followed by protein-protein interaction network analysis to identify hub genes. Their roles were further investigated through Gene Ontology, KEGG pathway analysis, tumor microenvironment immune infiltration, miRNA-regulatory network analysis, transcription factor prediction, and computational drug repurposing analyses.

**Results:**

Our ML‑driven approach identified seven hub genes central to DGC pathogenesis including *CCL5*, *CXCR4*, *MMP9*, *FOXP3*, *IL18*, *TNFSF11*, and *TNFSF13B*. Among these, three prioritized core hub genes (*CXCR4*, *MMP9*, and *TNFSF13B*) were selected based on statistically significant overexpression and complementary functional roles. Specifically, *CXCR4* showed a Fold Change of 3.12 (Log_2_FC = 1.64, FDR = 0.01), *MMP9* exhibited the highest magnitude of upregulation (Fold Change = 16.58, Log_2_FC = 4.05), and *TNFSF13B* demonstrated the most statistically significant differential expression (Fold Change = 2.32, Log_2_FC = 1.21, FDR < 0.000001). High *CXCR4* expression was identified as a potential prognostic indicator associated with poorer overall survival in the TCGA‑STAD cohort (HR = 1.5, p = 0.0072). We delineated a core regulatory circuitry where NF‑κB (NFKB1/RELA) masterfully regulates the hub gene network. Drug repurposing analysis nominated several FDA‑approved agents, including the VEGF‑A inhibitor Bevacizumab, which indirectly suppresses *CXCR4* and *MMP9*.

**Conclusion:**

This pilot study establishes a robust integrative framework that synergizes ML with network biology. It nominates *CXCR4* and *TNFSF13B* as candidate therapeutic targets and highlights the potential of ML‑based feature selection for discovering biologically relevant, context‑dependent prognostic indicators in DGC. However, we emphasize that these findings are exploratory and hypothesis‑generating, requiring independent validation through experimental studies and larger cohorts before any clinical translation.

## 1. Introduction

Gastric cancer (GC) is still burdened with a poor prognosis, ranking among the leading causes of cancer-related mortality worldwide [1]. According to the Lauren classification, GC histopathological subtypes are intestinal (IGC), diffuse (DGC), and mixed variants, which represent approximately 50%, 30%, and 20% of cases, respectively [2]. Over recent decades, IGC incidence has steadily declined, while DGC rates have relatively increased due to its elusive molecular mechanisms, late diagnoses, and poor response to conventional chemotherapy. DGC tends to aggressive growth in the gastric wall, with a preference for peritoneal dissemination and metastasis [3]. Unfortunately, DGC patients have a low survival rate, averaging approximately 18 months [4,5].

The pathogenesis of DGC is fundamentally different from that of IGC, which arises through environmentally driven stepwise progression. Hereditary diffuse gastric cancer (HDGC) develops by genetic processes [4,6]. In fact, HDGC is a rare autosomal-dominant syndrome (≤3%) that dramatically increases a person’s risk of developing DGC (67% male, 83% female) and lobular breast cancer risk (≤55% female) [5,7]. Most commonly, this disease is driven by germline mutation or inactivation in the E-cadherin gene (*CDH1*), and less commonly by pathogenic or likely pathogenic variant in other genes including *CTNNA1* (Catenin alpha-1), *RHOA* (Ras homolog family member A), *BRCA2* (breast cancer 2), *PALB2* (Partner and localizer of BRCA2), *STK11* (Serine/threonine kinase 11), *SDHB* (Succinate dehydrogenase), *PRSS1* (cationicc cationic trypsinogen), *ATM* (Ataxia-telangiectasia mutated), *MSR1* (Macrophage scavenger receptor 1), and *CLDN18-ARHGAP26* (claudin 18- Rho GTPase Activating Protein 26) fusions [8–10]. HDGC is typified by a strong family history, early onset (often before age 40), and a definitive primary prevention strategy in the form of prophylactic total gastrectomy for identified carriers [11]. In contrast, sporadic DGC may arise from acquired somatic mutations, without a significant family history, and lacks a hereditary component, thus shifting its management toward risk factor modification and symptomatic detection rather than prophylactic surgery. Both entities share similar histopathologic features, such as signet ring cell morphology (mucin‑filled cells with displaced and crescent‑shaped nuclei) and less differentiated cells at the base, which predominantly reside in the lamina propria or epithelial glands’ basement membrane [5]. This infiltrative nature complicates early endoscopic detection [3,7]. The indistinguishable histologic features of HDGC and sporadic DGC suggest shared progression mechanisms [5]. Research efforts were predominantly centered on identifying germline mutations and hereditary cancer syndromes, such as HDGC [12,13]. However, there is a substantially growing emphasis on elucidating alterations in gene expression and epigenetic regulation. While investigations specifically focused on early-onset gastric cancer (under 40 years) remain less extensive, an emerging consensus indicates that the dysregulation of gene expression, whether mediated by mutational, epigenetic, or alternative mechanisms, is central to the aggressive tumor biology observed in younger patients. As a result, this area of research is currently highly dynamic, with a clear trajectory toward the discovery and validation of gene expression-based biomarkers for improved prognostic stratification and targeted therapeutic strategies [14–17].

The high-dimensional nature of omics data, where the number of features (e.g., genes, proteins) vastly exceeds the number of samples (the “n << p” problem), presents challenges such as high risk of model overfitting, redundancy, and the increased computational cost and complexity [18]. In this regard, the feature selection methods have become an indispensable strategy in high-dimensional data analysis, offering substantial improvements in both dimensionality reduction and computational performance. By identifying and retaining the most informative variables, these methods effectively mitigate the risk of overfitting, enhance the scalability of subsequent modeling steps, and facilitate the extraction of robust, interpretable patterns from complex datasets [19]. Therefore, the feature selection methods offer a powerful approach in cancer research, particularly in genomics and proteomics, which is not merely a preprocessing step but a fundamental component of the analytical pipeline [20–23].

FS methods are broadly categorized into four classes: (1) filter methods, which rank features using statistical or information-theoretic metrics independent of the learning algorithm; (2) wrapper methods, which evaluate feature subsets based on model performance; (3) embedded methods, which integrate feature selection within the model training process; and (4) hybrid models [24–26]. Univariate filters (e.g., F-test) provide exceptional computational efficiency and scalability, making them ideal for a rapid initial reduction of feature space dimensionality. Crucially, because they evaluate features based on intrinsic statistical properties rather than a predictive model, they remain entirely independent of the classifier, thereby significantly mitigating the risk of overfitting. However, in the context of high-dimensional transcriptomic data, conventional univariate statistical methods often fail to capture complex feature interactions that may be critical for understanding disease pathogenesis [26]. To address this limitation, we employed the ReliefF (Regression ReliefF) algorithm, a robust multivariate feature selection technique. The multivariate filters, such as Minimum Redundancy Maximum Relevance (mRMR) and RReliefF, retain the benefits of model independence and reduced overfitting while introducing a critical additional capacity, *i.e*., the ability to identify and model complex dependencies and interactions between features, thus capturing a more holistic representation of the data’s underlying structure [27]. ReliefF is particularly well-suited for analyzing “small n, large p” datasets as it evaluates features based on their ability to distinguish between instances of different classes that are nearest neighbors in the multidimensional space. This approach enables the identification of genes involved in complex biological interactions that might be overlooked by univariate methods, making it ideal for uncovering the intricate molecular mechanisms underlying young-onset DGC [28].

To the best of our knowledge, this study is the first to apply machine learning-based feature selection to RNA-seq data for the analysis of DGC in patients younger than 40 years. The limited number of cases of young-onset DGC has made it difficult to conduct large-scale studies and our study is designed as a pilot investigation. Our primary aim is not to provide a final predictive model, but to test the feasibility of this integrated transcriptomic and ML framework. Ultimately, we seek to generate robust hypotheses about oncogenic pathways and candidate driver genes, thereby laying the groundwork for future validation studies with larger cohorts.

## 2. Materials and methods

### 2.1. Data acquisition and preprocessing

Early‑onset gastric cancer (≤45 years) more frequently presents as diffuse-type lesions arising in histologically normal mucosa, suggesting a greater role for genetic alterations compared to conventional GC (60–80 years), which is more strongly influenced by environmental factors [29]. Given that diffuse‑type gastric cancer consistently presents at a younger age than intestinal‑type gastric cancer, and to specifically investigate the molecular drivers of young‑onset DGC, we restricted our inclusion criteria to patients under 40 years of age. We performed a systematic search of the Gene Expression Omnibus (GEO) database (https://www.ncbi.nlm.nih.gov/) and selected all available samples that met the following criteria: first; all samples were under 40 years old, second; the cancerous tissues were diffuse type, and third; all samples were paired-end sequenced. Ultimately, 12 samples (6 normal and 6 tumor) from two datasets (GSE113255, GSE122401) were identified and selected for this pilot analysis (S1 Table). The mean age of patients with available age data in our cohort (n = 9 out of 12 samples) was 30.9 years (range: 25–35 years; SD: ± 3.4 years).

To assess and mitigate potential batch effects when combining datasets GSE113255 and GSE122401, we performed Principal Component Analysis (PCA) on the normalized expression matrix of all 12 samples (6 normal and 6 tumor) using the prcomp function in R version 4.3.2 (within the RStudio environment 2023.12.1) with scaling and centering [30]. Visualization was performed using the ggplot2 package to confirm the absence of significant batch-related clustering prior to downstream differential expression analysis.

**Fig 1:**
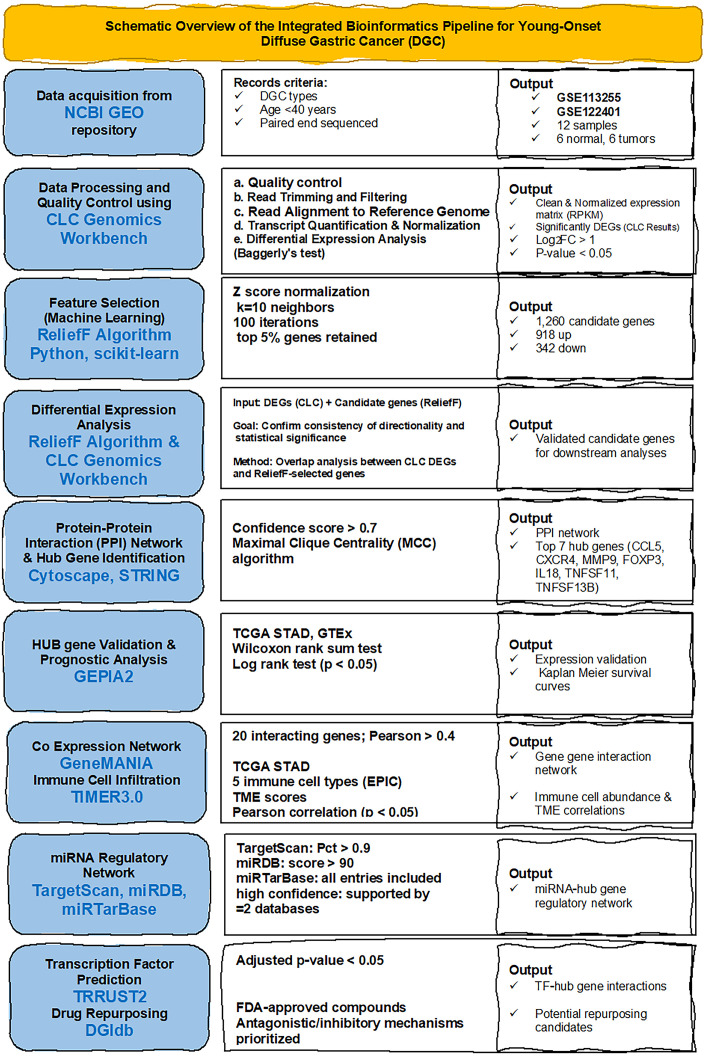
Comprehensive workflow of the bioinformatics analysis for identifying and validating hub genes in diffuse gastric cancer (DGC). The schematic outlines the sequential process from RNA‑seq data acquisition to multi‑omics validation. RNA‑seq data were acquired from the GEO public repository (GSE113255 and GSE122401), comprising 12 samples (6 normal and 6 DGC tissues) from patients under 40 years of age with paired‑end sequencing. After quality control, trimming, and alignment to the GRCh38 reference genome using CLC Genomics Workbench v20.0, differential expression analysis was performed using Baggerly’s test with thresholds of |Log_2_FC| > 1 and p-value < 0.05. Feature selection was conducted using the ReliefF algorithm on all 21,542 genes (k = 10, 100 iterations), retaining the top 5% (n = 1,260) for downstream analyses. Functional enrichment was performed using DAVID and SR plot (GO, KEGG; p-value < 0.05). Protein‑protein interaction networks were constructed via STRING (confidence > 0.7) and visualized in Cytoscape, with hub genes identified using the MCC algorithm (CytoHubba; top 7 nodes). Validation was conducted using GEPIA2 (TCGA‑STAD and GTEx) with Wilcoxon rank‑sum test and Log‑rank test (p < 0.05). Additional analyses included co‑expression networks (GeneMANIA; Pearson > 0.4), immune infiltration (TIMER3.0; 5 immune cell types, TME scores), miRNA regulation (TargetScan, miRDB, miRTarBase; high‑confidence: ≥ 2 databases), transcription factor prediction (TRRUST; adjusted p-value < 0.05), and drug repurposing (DGIdb; FDA‑approved compounds, antagonistic/inhibitory mechanisms prioritized).

The overall analytical workflow for identifying and validating hub genes in DGC is summarized in Fig 1, which illustrates the integrated bioinformatics pipeline encompassing data acquisition, preprocessing, feature selection, network analysis, and comprehensive multi-omics validation employed throughout this study.

### 2.2. Differential expression analysis of RNA-Seq Data

#### 2.2.1. Quality control assessment of raw reads.

The RNA-Seq data analysis protocol commenced with rigorous quality control (QC) to eliminate low-quality reads and ensure the reliability of downstream analyses. Initial QC steps incorporated GC content analysis via binomial expectation modeling to assess sequence composition bias, k-mer frequency profiling to detect overrepresented sequences, and ambiguous base quantification to filter reads containing indeterminate nucleotides (N). Read accuracy was further evaluated through Phred score distribution analysis across read lengths, while Sequencing Reads QC tools (*e.g*., FastQC) provided a comprehensive assessment of read integrity. Adapter contamination was specifically addressed using exact k-mer alignment against a curated database of Illumina adapter sequences, ensuring the removal of artifactual sequences before downstream processing [31].

#### 2.2.2. Read trimming and filtering.

Following QC assessment, the Trim Sequences Module in CLC Genomics Workbench v20.0 (Qiagen, Aarhus, Denmark; https://digitalinsights.qiagen.com/) was employed to process raw reads in FASTQ format [32,33]. The Trim Sequences tool uses a dynamic sliding window technique, calculating the average Phred quality score by traversing each read within a user-defined window (usually 5–15 bp). When the average quality fell below a threshold of Q20, bases from the ends of the reads were trimmed. Additionally, adapter sequences were eliminated by combining approximate matching based on a modified Levenshtein distance algorithm with exact k-mer matching, permitting a small number of mismatches. To maintain data integrity for subsequent analyses, reads shorter than 50 bp after trimming were excluded. Through this process, clean, high-quality data were generated for all downstream analyses.

#### 2.2.3. Read alignment to reference genome.

The high-quality, trimmed reads were then aligned to the human reference genome GRCh38/hg38 in FASTA format, downloaded from the ENSEMBL database [34]. Alignment was performed using the RNA-Seq Analysis pipeline in CLC Genomics Workbench. Read mapping leveraged a seed-and-extend alignment strategy, where initial seeds were identified using hash tables, and alignments were extended through dynamic programming based on a modified Smith-Waterman algorithm. Splice junctions were predicted using a Maximum Entropy Model trained on annotated exon-intron boundaries, thereby enhancing the accurate detection of spliced alignments. Mapping parameters were optimized for high specificity, allowing a maximum of two mismatches per read, requiring a minimum of 90% sequence identity, and filtering out multi-mapped reads based on alignment scores. Following successful alignment, transcript quantification was subsequently performed, and expression values were normalized using the Reads Per Kilobase of transcript per Million mapped reads (RPKM) method for subsequent differential expression analysis between tumor and normal samples [33].

#### 2.2.4. Differential expression analysis and statistical assessment.

To identify statistically significant differentially expressed genes between tumor and normal samples, we employed the proportion‑based statistical analysis tool implemented within the CLC Genomics Workbench, which utilizes Baggerly’s test. Baggerly’s test is a rank‑based statistical method designed to compare expression proportions between two groups by evaluating the distribution of read counts across biological conditions. Unlike parametric tests that assume normal distribution and equal variances, Baggerly’s test is robust to heteroscedasticity and does not require equal variance between groups, making it particularly suitable for small sample sizes and datasets with unbalanced group variances, characteristics inherent to our rare DGC dataset. Genes were considered significantly differentially expressed if they met the criteria of an absolute log_2_ fold change > 1 and a p‑value < 0.05 [35].

### 2.3. Feature selection and identification of candidate genes via machine learning

#### 2.3.1. Data standardization and preparation for machine learning.

A Python (v3.11.12) script was used for preprocessing of the transcriptomic data obtained from CLC Genomics Workbench v20.0 analysis (S2 Table). The data set was transposed that genes were the features (columns), and samples were the observations (rows). A label vector was created with a class of ‘0’ for the six tumor (T) samples and ‘1’ for the six normal (N) samples. Given the pilot and exploratory nature of the current study with the overall aim of finding a robust collection of candidate genes rather than training a general predictive model, all available samples (n = 12) were utilized for feature selection. This approach maximizes detection power for differential expression patterns in this rare population. The feature values were subsequently normalized with the Z-score normalization (std_dev = 1, mean = 0) to avoid biasing the algorithms, particularly the distance-based ReliefF, by the inherent variation of the scales of gene expression.

#### 2.3.2. Candidate gene selection using machine learning algorithm.

To identify the most informative genes associated with DGC, we applied the ReliefF algorithm to the entire normalized expression matrix containing all 21,542 genes. ReliefF is a multivariate feature selection method that assigns a weight to each feature based on its ability to discriminate between instances of different classes (tumor vs. normal) by evaluating the nearest neighbors in the multidimensional space. This approach enables the identification of genes involved in complex biological interactions that might be overlooked by univariate methods. The analysis was implemented in Python 3.10 using scikit-learn (v1.2.2) within Google Colab (https://colab.research.google.com/), configured with k = 10 nearest neighbors and 100 iterations. Genes were then ranked by their ReliefF weights in descending order [36–38]. To reduce computational complexity for downstream analyses while maintaining biological relevance, we retained the top 5% of genes (n = 1,260) for subsequent network and functional enrichment analyses.

### 2.4. Functional annotation and pathway enrichment analysis of candidate genes

To elucidate the biological functions and signaling pathways associated with the candidate genes identified through ReliefF-based feature selection, functional enrichment analysis was performed on the candidate genes identified by the ReliefF algorithm using the Database for Annotation, Visualization, and Integrated Discovery (DAVID, v6.8; https://david.ncifcrf.gov) and SR plot online (https://bioinformatics.com.cn/en). The analysis identified significantly enriched Gene Ontology (GO) terms for biological processes (BP), cellular components (CC), and molecular functions (MF), as well as Kyoto Encyclopedia of Genes and Genomes (KEGG) pathways. Statistical significance was defined using a false discovery rate (FDR) threshold of < 0.05, calculated via the Benjamini-Hochberg method to correct for multiple testing [39,40].

### 2.5. Construction of protein-protein interaction network and hub gene identification

To explore the functional relationships and identify the most central genes among the candidate set, a protein-protein interaction (PPI) network was constructed for the candidate genes identified by the ReliefF algorithm using the StringApp plugin (v2.2.0) in Cytoscape (v3.10.3). All interaction types (predicted and experimentally validated) were retrieved from the STRING database (v11.5) with a minimum confidence score threshold of 0.7. From the resulting network, hub genes were identified using the CytoHubba plugin (v0.1) by applying the Maximal Clique Centrality (MCC) algorithm. The top 7 nodes ranked by MCC score were selected as hub genes for subsequent validation and prognostic analysis [41,42]. While PPI networks derived from transcriptomic data provide a useful framework for understanding potential functional interactions, it is important to note that mRNA levels do not always reflect protein activity. However, these predictions assume a direct relationship between gene expression and protein function.

To confirm the consistency of expression patterns, the candidate hub genes selected via CytoHubba were further compared with the differential expression results obtained from the CLC Genomics Workbench v20.0 RNA-Seq analysis pipeline. Genes were considered significantly differentially expressed if they met the criteria of an absolute log_2_ fold change > 1 and a p-value < 0.05 [35]. This comparison aimed to confirm the directionality (upregulated or downregulated) and statistical significance of the selected hub genes prior to downstream validation.

### 2.6. Validation of Hub genes’ expression and prognostic significance

To visually confirm the differential expression of the hub genes within our own dataset, violin plots comparing their expression levels in DGC tumors versus normal tissues were generated using R version 4.3.2 within the RStudio environment (2023.12.1).

For external validation using independent public datasets, the expression patterns and prognostic significance of the hub genes were assessed using the Gene Expression Profiling Interactive Analysis (GEPIA2, v2.0; http://gepia2.cancer-pku.cn) platform. GEPIA2 is an interactive web-based tool that integrates RNA-seq expression data from The Cancer Genome Atlas (TCGA) stomach adenocarcinoma (STAD) cohort and normal tissue samples from the Genotype-Tissue Expression (GTEx) project, providing a robust reference for validation. Survival analysis was performed through GEPIA2 by generating Kaplan-Meier curves, in which patients from the TCGA-STAD cohort were stratified into high and low expression groups based on the median expression level of each hub gene. Statistical significance of survival differences was determined using the log-rank test, with a significance threshold of p < 0.05. Differential expression of hub genes between TCGA tumor and GTEx normal tissues was visualized using box plots, with significance assessed by GEPIA2 via the Wilcoxon rank-sum test [43].

### 2.7. Co-expression and transcriptional regulatory network analysis

To investigate the functional associations, potential co-expression patterns, and regulatory relationships of the identified hub genes, we constructed a gene-gene interaction network using GeneMANIA (v3.6.0; http://www.genemania.org). GeneMANIA is a web-based tool that predicts gene function and identifies functionally similar genes by integrating multiple genomic and proteomic interaction databases. The analysis was performed using all 7 hub genes identified through PPI network analysis, queried against the Homo sapiens genome-wide dataset with a limit of 20 resultant genes per query. The network integrated multiple interaction types, including co-expression, physical interactions, genetic interactions, and pathway co-membership derived from databases such as BioGRID and STRING. Interactions were weighted by confidence scores, with co-expressed genes requiring a Pearson correlation coefficient of > 0.4 for inclusion. Functional enrichment analysis of the genes within the resulting network was performed using GeneMANIA’s built-in features to identify overrepresented biological pathways and functional categories associated with the hub genes and their interacting partners [44].

### 2.8. Evaluation of immune cell infiltration patterns associated with hub genes

To investigate the potential impact of the identified hub genes on the tumor immune microenvironment (TME) and their association with immune cell infiltration, the relationship between the 7 hub genes and the TME was evaluated using the TCGA-STAD cohort as a validation dataset via the TIMER3.0 web server (https://compbio.cn/timer3/). TIMER3.0 is a comprehensive web-based platform that systematically analyzes immune cell infiltration across multiple cancer types using TCGA and other public datasets. Although the STAD cohort comprises multiple histological subtypes, it provides a robust platform for the initial validation of TME interactions in gastric cancer.

To comprehensively characterize the TME, the following analyses were performed: First, the abundance of five immune cell types (B cells, CD8 + T cells, CD4 + T cells, macrophages, and natural killer (NK) cells) was estimated via the EPIC (Estimating the Proportion of Immune and Cancer cells) method, which uses deconvolution of bulk transcriptomic data to infer immune cell fractions. Second, to gain a global view of the TME composition, we extracted three key scores for each sample: the Immune Score_ESTIMATE (reflecting infiltrating immune cells), Stroma Score_ESTIMATE (reflecting stromal presence), and Estimate Score_ESTIMATE (inversely correlating with tumor purity). Additionally, the MSI Score_TIDE was retrieved as a predictor of microsatellite instability and potential immune escape. To assess the associations between hub gene expression and TME characteristics, Pearson’s correlation analysis was performed between each hub gene’s expression level (log2(TPM + 1)) and each of the immune cell abundances and TME scores obtained from TIMER3.0. Statistical significance was defined as p < 0.05, with both correlation coefficients (R) and corresponding p-values reported for complete interpretation of the relationships [45,46].

### 2.9. Post-transcriptional regulatory network of hub genes

To identify potential microRNA (miRNA) regulators of the hub genes, a comprehensive in silico analysis was performed using three complementary databases: TargetScan (https://www.targetscan.org/vert_80/) for evolutionarily conserved miRNA-binding sites, miRDB (https://mirdb.org/) for machine-learning-based predictions, and miRTarBase (https://mirtarbase.cuhk.edu.cn/) for experimentally validated interactions. Database-specific quality thresholds were first applied, retaining only predictions with a probability of conserved targeting (Pct) > 0.9 in TargetScan and a target score > 90 in miRDB; all entries in miRTarBase were included to capitalize on available experimental evidence. To finalize a high-confidence set, only miRNA-gene interactions supported by at least two of the three databases were considered. Finally, the resulting high-confidence miRNA-hub gene regulatory network was visualized using SR plot online [47–50].

### 2.10. Therapeutic repurposing opportunities targeting hub genes

To identify potential therapeutic agents that could be repurposed for targeting the identified hub genes, the 7 prioritized hub genes were systematically queried against the DGIdb (Drug-Gene Interaction Database, version 5.0; https://dgidb.org/). DGIdb is a comprehensive, publicly available resource that integrates drug-gene interaction data from over 30 curated sources, including DrugBank, PharmGKB, and ChEMBL. The search was configured to retrieve only interactions with known drug categories and evidence derived from curated sources. To prioritize clinically actionable candidates, resulting drug-gene pairs were filtered to highlight interactions specifically with existing FDA-approved compounds, with a particular focus on those with antagonistic or inhibitory mechanisms against the hub gene products, as these are most likely to offer therapeutic benefit in cancer treatment. This approach aimed to prioritize candidates for subsequent experimental validation [51].

### 2.11. Transcriptional regulatory network governing hub gene expression

To identify potential transcription factors (TFs) that regulate the expression of the identified hub genes at the transcriptional level, we employed TRRUST version 2 (Transcriptional Regulatory Relationships Unraveled by Sentence-based Text mining; https://www.grnpedia.org/trrust/). TRRUST is a curated database of transcriptional regulatory networks in human and mouse, constructed through systematic text mining of PubMed abstracts, providing experimentally validated and literature-curated TF-target interactions. The analysis was conducted using the database’s built-in prediction tools to ascertain TFs that are significantly associated with the hub gene set. TF-hub gene interactions were considered significant if they met the threshold of an adjusted p-value < 0.05, and the direction of regulation (activation or repression) was recorded where available from the database [52].

## 3. Results

### 3.1. Identification of differentially expressed genes (DEGs) using feature selection methods

PCA of the combined dataset confirmed samples clustered primarily by tissue type (tumor vs. normal) rather than by dataset origin, with no apparent separation between the two GEO series, supporting the feasibility of combining these datasets (S1 Fig). Normal samples from both datasets (GSE113255 and GSE122401) formed a distinct cluster, indicating similar expression profiles regardless of dataset origin. Interestingly, two tumor samples (SRR7014369 and SRR8281398) clustered near normal samples, reflecting the known molecular heterogeneity of DGC and the intrinsic variability of tumor samples. These findings confirm the absence of significant batch effects and support the feasibility of combining GSE113255 and GSE122401 for downstream analyses. Therefore, no batch correction was applied, as doing so could inadvertently remove true biological variation and introduce bias, particularly given the limited sample size (n = 6 per group).

The ReliefF algorithm was applied to the full expression matrix of all 21,542 genes. Based on the ReliefF weights, genes were ranked by their discriminatory power between tumor and normal samples. From this ranking, we selected the top 5% of genes (n = 1,260) with the highest ReliefF weights as candidate features for downstream analysis. To determine the direction of expression changes, we compared the mean expression values of each gene between tumor and normal samples. Genes with higher mean expression in tumor samples were classified as upregulated, while those with lower mean expression in tumor samples were classified as downregulated. Among the 1,260 selected genes, 918 were upregulated and 342 were downregulated in cancerous tissues compared to normal tissues. S3 Table summarizes the top 50 upregulated and downregulated genes among those selected by the ReliefF algorithm, based on their mean expression differences between tumor and normal samples. Additionally, the complete results of the differential expression analysis performed using CLC Genomics Workbench v20.0 and the ReliefF algorithm are provided in S4 and S5 Tables, respectively.

### 3.2. Functional enrichment analysis

To functionally characterize the selected genes, KEGG pathway and Gene Ontology (GO) enrichment analyses were conducted using the DAVID bioinformatics database (2021 update) and SRplot. GO analysis revealed significant enrichment of biological processes (BP) in both upregulated and downregulated gene sets. Upregulated genes were associated with intermediate filament organization (GO:0045109), telomere maintenance (GO:0000723), response to ATP (GO:0033198), and regulation of the cell cycle (GO:0051726). However, downregulated genes were enriched in lipid metabolic processes (GO:0006629), transport across the blood-brain barrier (GO:0150104), fatty acid biosynthesis (GO:0006633), and immune response (GO:0006955) (S2 Fig).

Molecular function (MF) analysis highlighted protein binding (GO:0005515), transcription corepressor binding (GO:0001222), ATP hydrolysis activity (GO:0016887), and GTPase activity (GO:0005096) as key functions of upregulated genes. Downregulated genes were linked to antigen binding (GO:0003823), nucleotide binding (GO:0000166), linoleate 9S-lipoxygenase activity (GO:1990136), and calcium ion binding (GO:0005509) (S3 Fig).

Cellular component (CC) analysis demonstrated that upregulated genes localized predominantly to the nucleoplasm (GO:0005654), cytosol (GO:0005829), T cell receptor complex (GO:0042101), intracellular membrane-bounded organelles (GO:0043231), and nucleus (GO:0005634), whereas downregulated genes were enriched in the immunoglobulin complex (GO:0019814), apical plasma membrane (GO:0016324), neuron projections (GO:0043005), and mitochondria (GO:0005739) (S4 Fig).

KEGG pathway analysis identified distinct signaling cascades enriched in DEGs. Upregulated genes were significantly associated with T helper 17 (Th17) cell differentiation (hsa04659), intestinal immune network for IgA production (hsa04672), *Yersinia* infection (hsa05135), and tuberculosis (hsa05152), while downregulated genes were enriched in metabolic pathways (hsa01100), oxidative phosphorylation (hsa00190), chemical carcinogenesis–reactive oxygen species (hsa05208), glycosphingolipid biosynthesis (hsa00603), and amyotrophic lateral sclerosis (hsa05014) (S5 Fig). The complete Gene Ontology (BP, MF, CC) and KEGG pathway enrichment results for the upregulated and downregulated genes are provided in S6 Table.

### 3.3. Analysis of protein-protein interaction network and Hub gene modules

**Fig 2:**
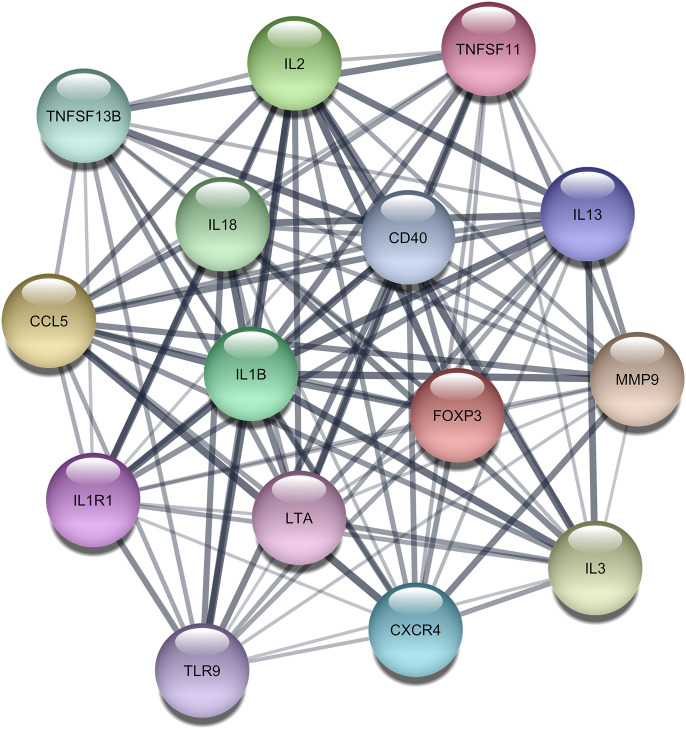
Identification of hub genes using the Maximal Clique Centrality (MCC) algorithm in Cytoscape. Hub genes were prioritized via the cytoHubba plugin using MCC scoring (threshold: 14.571). Cytoscape (v. 3.10.3) was used for visualization.

In biological systems, biomolecules operate through coordinated interactions. To investigate the functional relationships among the selected genes, a PPI network was constructed using the STRING database for common proteins across the datasets. Subsequent analysis of the PPI network via the cytoHubba plugin in Cytoscape identified the initial seven hub genes including, *CCL5* (chemokine ligand 5), *CXCR4* (C-X-C chemokine receptor type 4 or RANTES), *MMP9* (Matrix Metallopeptidase 9, gelatinase-B), *FOXP3* (forkhead box P3), *IL18* (Interleukin 18), *TNFSF11* (TNF Superfamily Member 11 or RANKL), and *TNFSF13B* (TNF Superfamily Member 13b encoding, BAFF or BLYS cytokine) that exhibited the highest topological significance (*e.g*., degree centrality) compared to other nodes in the network (Fig 2).

Following the identification of candidate hub genes through a ReliefF-based feature selection analysis, the results were compared with the differential expression results obtained from the CLC Genomics Workbench v20.0 pipeline (Table 1). As shown in Table 1, all seven hub genes showed consistent directional changes (upregulated or downregulated) between the ReliefF and CLC differential expression analyses. In the CLC analysis, *MMP9* demonstrated the most substantial fold change (16.58), followed by *TNFSF11* (4.73) and *CXCR4* (3.12). However, only two genes reached statistical significance in the conventional differential expression test including *CXCR4* (FDR = 0.01) and *TNFSF13B* (FDR < 0.000001). The remaining genes, including *CCL5* (FDR = 1.00), *FOXP3* (FDR = 0.14), *IL18* (FDR = 0.82), *MMP9* (FDR = 0.34), and *TNFSF11* (FDR = 0.06), did not achieve significance in the univariate analysis. This discrepancy is expected given the fundamental differences between univariate and multivariate approaches, *i.e.,* univariate tests assess each gene independently and are limited by statistical power, while multivariate methods like ReliefF capture complex interactions and coordinated expression patterns that may not be detectable in single-gene analyses. The complementary nature of these approaches, univariate tests identifying genes with large individual effects and ReliefF identifying genes with coordinated multivariate signals, provides a more comprehensive understanding of the molecular landscape of DGC.

**Table 1:** Comparative analysis of hub gene expression from the ReliefF-based feature selection algorithm and the CLC Genomics Workbench v20.0 differential expression pipeline.

Feature ID	ReliefF Weights	Mean Expression (Normal Mean RPKM)	Mean Expression (Tumor Mean RPKM)	Direction (ReliefF-based)	Fold Change (Baggerley’s test, CLC)	FDR p-value (Baggerley’s test, CLC)
**CCL5**	0.070	977.42	1025.07	Up	1.09	1.00
**CXCR4**	−0.017	1116.93	3318.95	Up	3.12	**0.01**
**FOXP3**	−0.015	43.25	126.00	Up	3.04	0.14
**IL18**	0.027	395.80	313.68	Down	−1.22	0.82
**MMP9**	0.033	146.40	2343.65	Up	16.58	0.34
**TNFSF11**	−0.005	10.93	50.60	Up	4.73	0.06
**TNFSF13B**	0.014	173.60	413.10	Up	2.32	**< 0.000001**

The ReliefF weights for the seven hub genes are also presented in Table 1. Among the hub genes, *CCL5* exhibited the highest ReliefF weight (0.0699), indicating its strong multivariate discriminatory power between tumor and normal samples. *MMP9* (0.0334) and *IL18* (0.0274) also showed positive weights, confirming their contribution to class separation. *TNFSF13B* (0.0141) displayed a weak positive weight, while *TNFSF11* (−0.0045), *FOXP3* (−0.0152), and *CXCR4* (−0.0165) showed negative or near-zero weights, suggesting limited contribution to multivariate class separation despite their significance in univariate differential expression or PPI network centrality. Notably, *CXCR4* demonstrated a high fold change (3.12) but a negative ReliefF weight (−0.0165), highlighting the fundamental difference between univariate and multivariate feature selection approaches. While *CXCR4* shows differential expression between tumor and normal tissues in univariate analysis, its multivariate discriminatory power is limited, possibly due to heterogeneous expression patterns across samples or its interaction with other genes in the network. These findings underscore the complementary value of ReliefF in identifying genes with true multivariate discriminatory power, and together with PPI network analysis, provide a robust framework for prioritizing biologically relevant hub genes in DGC.

### 3.4. Gene enrichment analysis of hub genes

GO enrichment analysis of the hub genes, conducted using the DAVID database, highlighted their significant involvement in critical biological processes, including immune response, inflammatory response, response to viral infection, positive regulation of the phosphatidylinositol 3-kinase/protein kinase B (PI3K/AKT) signaling pathway, and positive regulation of homotypic cell-cell adhesion. CC analysis revealed enrichment of hub genes in the extracellular space, extracellular region, and cytoplasm. Also, MF analysis further identified two key functional annotations, *i.e*., cytokine activity and TNF receptor binding (S6 Fig). The complete Gene Ontology (BP, MF, CC) enrichment results for the hub genes are provided in S6 Table.

### 3.5. Validation and Prognostic value of hub genes

**Fig 3:**
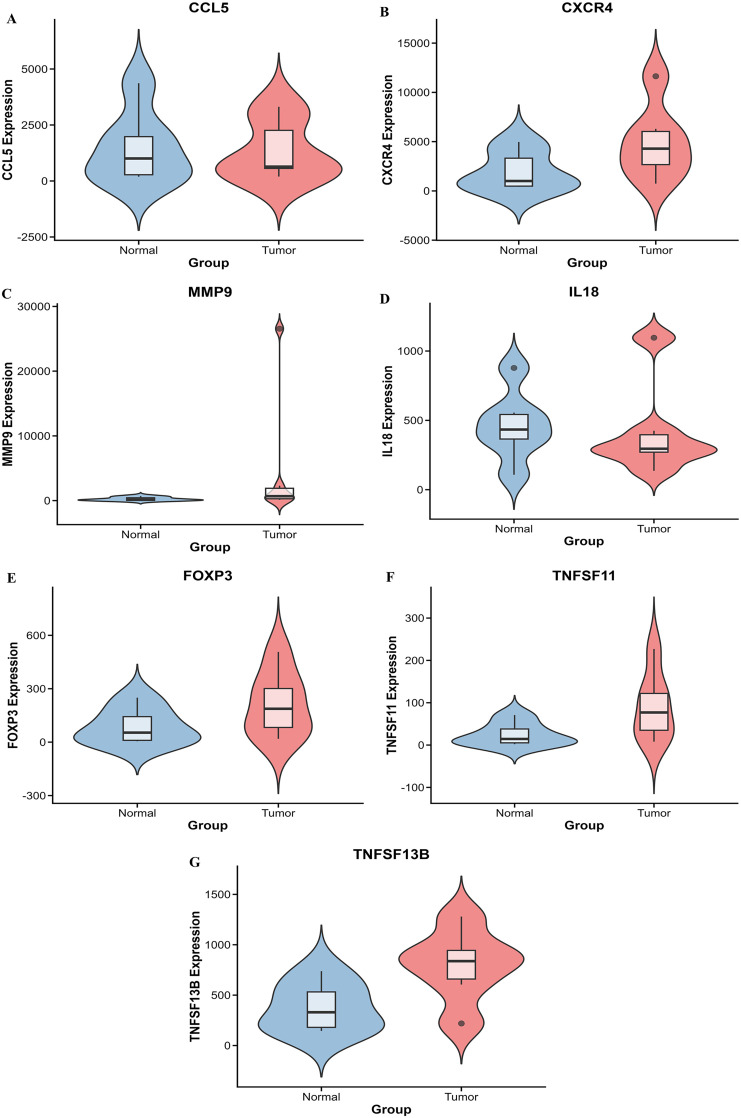
Violin plots comparing Hub gene expression distributions between diffuse gastric cancer (DGC) tissues and normal samples. The y‑axis represents ‘Expression (Raw Read Counts)’, and the x‑axis indicates the sample groups (Tumor vs. Normal). Central lines within the plots denote the median expression, while the box boundaries indicate the interquartile range (IQR). This visualization highlights significant differences in expression profiles, including variations in distribution shape, spread, and central tendency, underscoring transcriptomic alterations in DGC relative to normal tissue. (A) *CCL5*, (B) *CXCR4*, (C) *MMP9*, (D) *IL18*, (E) *FOXP3*, (F) *TNFSF11*, and (G) *TNFSF13B*.

**Fig 4:**
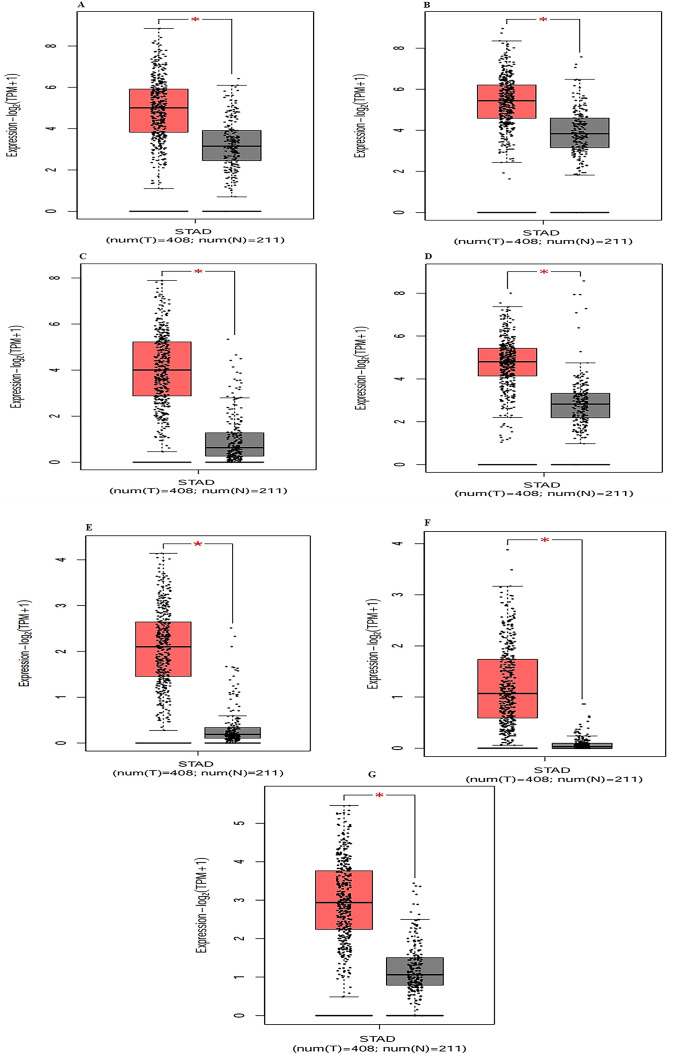
Expression validation of the seven network-derived hub genes in gastric cancer. Box plots comparing mRNA expression levels between gastric tumor tissues (red; n = 408) and matched normal tissues (gray; n = 211) from the Stomach Adenocarcinoma (STAD) cohort in the GEPIA database. The validated hub genes are: (A) *CCL5*, (B) *CXCR4*, (C) *MMP9,* (D) *IL18*, (E) *FOXP3*, (F) *TNFSF11*, and (G) *TNFSF13B*.

Violin plots illustrating the distribution of expression levels in DGC tissues compared to normal samples are presented in Fig 3. Additionally, to evaluate the differential expression of hub genes in GC, we analyzed transcriptomic data from tumors and normal tissues using GEPIA, a widely recognized platform for cancer-specific expression profiling. A comparative boxplot visualization revealed distinct expression patterns for the hub genes across these tissue types (Fig 4).

**Fig 5:**
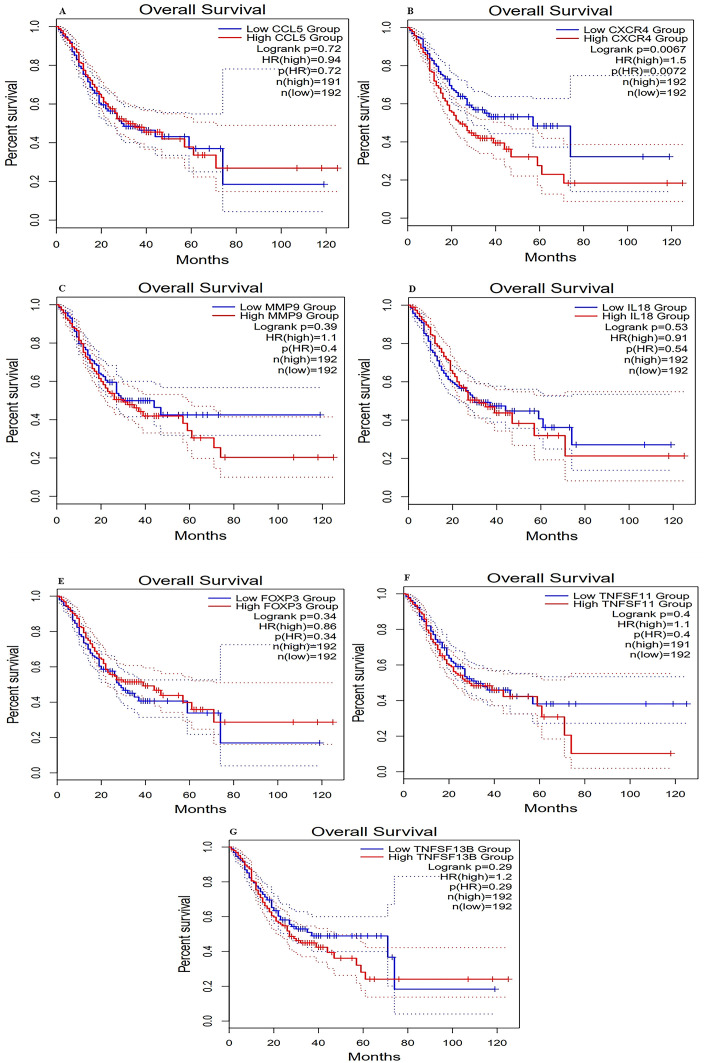
Prognostic value of hub genes in gastric cancer. Kaplan-Meier overall survival curves for gastric cancer patients from the GEPIA database, stratified by high (red) and low (blue) expression of the seven hub genes: (A) *CCL5*, (B) *CXCR4*, (C) *MMP9,* (D) *IL18*, (E) *FOXP3*, (F) *TNFSF11*, and (G) *TNFSF13B*. The hazard ratio (HR) and p-value from the logrank test are indicated for each analysis.

We further assessed the prognostic significance of the initial seven hub genes by generating overall survival (OS) curves in GEPIA, stratified by gene expression levels (Fig 5). Hazard ratio (HR) analysis corroborated these findings: *CCL5* (HR = 0.94, p = 0.72), *IL18* (HR = 0.91, p = 0.54), *TNFSF11* (HR = 1.1, p = 0.4), *TNFSF13B* (HR = 1.2, p = 0.29), *CXCR4* (HR = 1.5, p = 0.0072), *MMP9* (HR = 1.1, p = 0.4), *FOXP3* (HR = 0.86, p = 0.34). Notably, elevated *CXCR4* expression (measured in transcripts per million, TPM) was also associated with a statistically significant 1.5-fold increase in mortality risk (*p* < 0.01), suggesting it as a candidate prognostic marker in GC requiring further validation.

### 3.6. Co-expression network of hub genes

**Fig 6:**
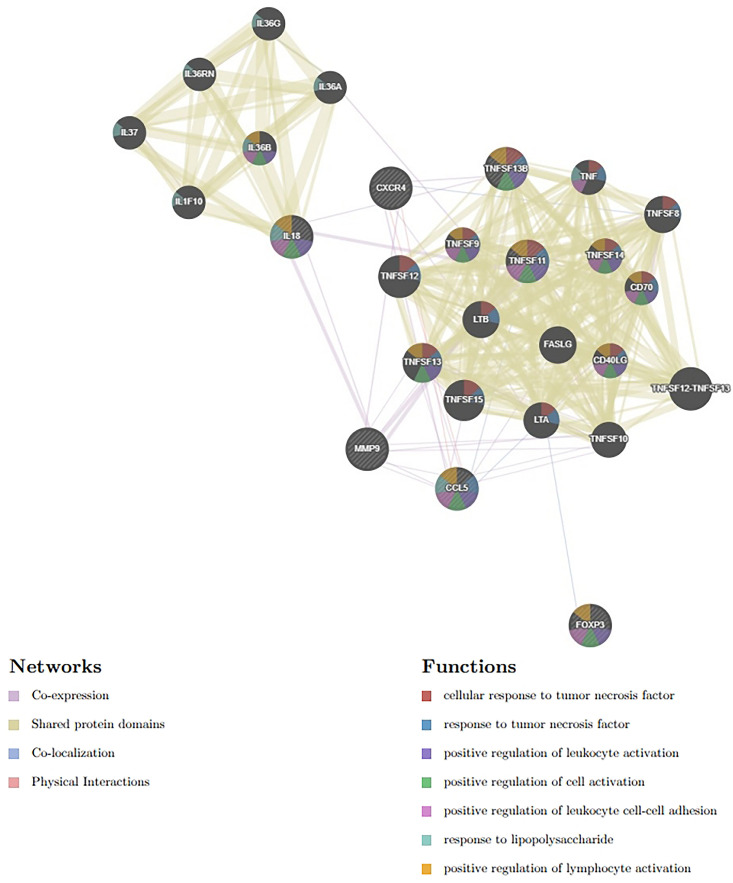
Co-expression network and functional enrichment of hub genes in gastric cancer. The network, generated using GeneMANIA, depicts interactions between the identified hub genes. Connecting lines (edges) represent predicted functional associations: purple for co-expression and red for physical interactions. The colored nodes represent the hub genes, with the node color indicating their primary associated biological function, as defined in the legend.

Co-expression network analysis of hub genes, performed using GeneMANIA, revealed robust co-expression relationships between the identified hub genes and key members of TNF superfamily (*TNFSF12*-*TNFSF13*, *TNFSF15*, *TNFSF8*, *TNFSF10*, *TNFSF14*, *FASLG*, *LTB*, *LTA*, *TNFSF9*, *TNF*, *CD40LG*, *CD70*), as well as IL-1 family (*IL1F10*, *IL36G*, *IL37*, *IL36RN*, and *IL36A*), and *IL12B*. Functional enrichment analysis of co-expressed genes highlighted their significant association with cancer-related biological processes, including cellular response to TNF, positive regulation of leukocyte and lymphocyte activation, positive regulation of cell-cell adhesion, and response to lipopolysaccharide (Fig 6).

### 3.7. Immune cell infiltration analysis of hub geness

Our comprehensive TME analysis revealed that all hub genes except *IL-18* showed statistically significant associations with TME components, reflecting their distinct biological roles (Table 2). *CCL5* demonstrates the strongest overall immune signature (Immune Score: R = 0.79; Estimate Score: R = 0.68; Stroma Score: R = 0.46; all adj p < 0.0001), consistent with its known function as a potent chemokine recruiting T lymphocytes and macrophages. *TNFSF13B* exhibited robust dual and significant associations with both immune and stromal components (Immune Score: R = 0.76; Estimate Score: R = 0.73; Stroma Score: R = 0.58; all adj p < 0.0001), aligning with its role in B-cell activation and humoral immune responses. *FOXP3*, a specific regulatory T-cell (Treg) marker, showed strong and significant immune correlations (Immune Score: R = 0.65, adj p < 0.0001), confirming its expression reflects Treg presence in tumors. *CXCR4* displayed significant involvement across all TME aspects (Immune Score: R = 0.62; Stroma Score: R = 0.57; Estimate Score: R = 0.65; all adj p < 0.0001), supporting its dual role in metastasis and immune cell recruitment. *MMP9* demonstrated strong stromal associations (Stroma Score: R = 0.30, adj p < 0.0001), expected its function in extracellular matrix (ECM) remodeling. *TNFSF11* showed a distinct pattern with significant MSI Score association (R = −0.2737, adj p < 0.0000), suggesting potential relevance to immunotherapy response. In contrast, IL-18 showed no significant TME correlations, indicating its role in GC may operate through mechanisms independent of cellular composition (Table 2).

**Table 2:** Correlation analysis between hub gene expression and tumor microenvironment characteristics in gastric cancer estimated via TIMER3.0. Spearman’s correlation coefficients (R) and adjusted p-values are shown for associations between hub genes and immune infiltration scores (Immune Score, Stroma Score, Estimate Score, MSI Score). Colors represent Spearman’s correlation strength (R) ranging from −1.0 (blue, negative correlation) to +1.0 (red, positive correlation).

Infiltrates/ Score	*CXCR4*	*CCL5*	*TNFSF11*	*TNFSF13B*	*MMP9*	*FOXP3*	*IL18*
**Immune Score**	0.6174 (adj p < 0.0000)	0.7917 (adj p < 0.0000)	0.1668 (adj p = 0.0037)	0.7642 (adj p < 0.0000)	0.4128 (adj p < 0.0000)	0.6475 (adj p < 0.0000)	0.0777 (adj p = 0.2900)
**Stroma Score**	0.5687 (adj p < 0.0000)	0.4613 (adj p < 0.0000)	0.2824 (adj p < 0.0000)	0.5810 (adj p < 0.0000)	0.2962 (adj p < 0.0000)	0.3976 (adj p < 0.0000)	0.0121 (adj p = 0.9466)
**Estimate Score**	0.6521 (adj p < 0.0000)	0.6799 (adj p < 0.0000)	0.2424 (adj p < 0.0000)	0.7316 (adj p < 0.0000)	0.3805 (adj p < 0.0000)	0.5621 (adj p < 0.0000)	0.0465 (adj p = 0.5794)
**MSI Score**	−0.0842 (adj p = 0.1296)	0.0237 (adj p = 0.6744)	−0.2737 (adj p < 0.0000)	0.0136 (adj p = 0.8304)	−0.1276 (adj p = 0.0198)	−0.1499 (adj p = 0.0050)	0.0949 (adj p = 0.1754)

**Fig 7:**
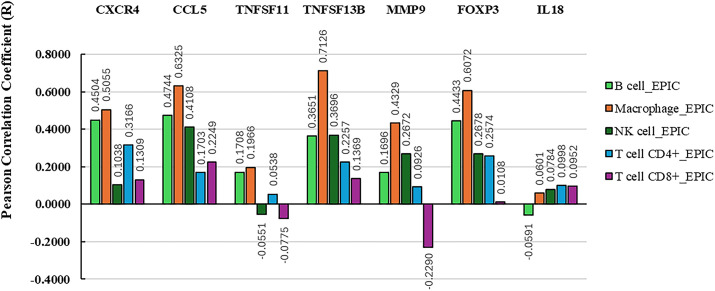
Correlation of hub genes with immune cell infiltration in gastric cancer. Bar plots showing Spearman’s correlation coefficients (R) between the expression of the seven hub genes (*CCL5*, *CXCR4*, *MMP9*, *FOXP3*, *IL18*, *TNFSF11*, and *TNFSF13B*) and the abundance of five immune cell subsets (B cells, Macrophages, NK cells, CD4 + T cells, and CD8 + T cells) in the TCGA‑STAD cohort. Immune cell proportions were estimated using the EPIC algorithm via the TIMER3.0 web server. The height of each bar represents the correlation coefficient (R), with positive values indicating a positive association and negative values indicating a negative association. The exact R value is displayed above each bar for precise interpretation.

Analysis of hub gene associations with specific immune cell populations in GC revealed distinct infiltration patterns, with macrophage interactions being most prominent (Fig 7). *TNFSF13B* demonstrated the strongest overall correlation with macrophages (R = 0.71, adj p < 0.0001), followed by *CCL5* (R = 0.63, adj p < 0.0001) and *FOXP3* (R = 0.61, adj p < 0.0001). B cell infiltration showed significant positive correlations across multiple hub genes, particularly *CCL5* (R = 0.47, adj p < 0.0001), *CXCR4* (R = 0.45, adj p < 0.0001), and *FOXP3* (R = 0.44, adj p < 0.0001). T CD4 + cell associations were strongest with *CXCR4* (R = 0.32, adj p < 0.0001) and *FOXP3* (R = 0.26, adj p < 0.0001), while CD8 + T cell correlations were generally weaker, with *CCL5* showing the strongest positive association (R = 0.22, adj p < 0.0001). Notably, MMP9 exhibited a significant negative correlation with T CD8 + cells (R = −0.23, adj p < 0.0001), and TNFSF11 showed minimal associations across most immune cell types. IL-18 consistently demonstrated no significant correlations with any immune cell population. These findings highlight the specialized roles of hub genes in recruiting and modulating specific immune cell subsets within the gastric cancer microenvironment. The detailed correlation analysis between hub gene expression and immune cell infiltration is summarized in S7 Table.

### 3.8. miRNA-hub genes analysis

Our integrated bioinformatic analysis identified a high-confidence network of miRNA-gene interactions potentially central to DGC pathogenesis. The hub genes were extensively targeted by miRNAs, with several key regulators emerging (Table 3). Notably, *CXCR4* was strongly regulated by multiple miRNAs, including miR-139-5p and miR-9-5p, both supported by all three databases and strong experimental validation (e.g., Western blot, qPCR). The *MMP9* gene was also strongly regulated by hsa-miR-149-5p based on the miRTarBase database. Similarly, the immunomodulatory gene *TNFSF11* (RANKL) was potentially co-regulated by hsa-miR-18a-5p, supported by the miRTarBase database. Additionally, *TNFSF13b* (BAFF) was targeted by a cohesive set of miRNAs, all supported by a consensus of TargetScan and miRDB. These results delineate a complex post-transcriptional regulatory network in DGC, highlighting specific miRNA families as potential master regulators of key hub genes involved in tumor immunity and progression. The complete list of predicted miRNA‑hub gene interactions is provided in S8 Table.

**Table 3:** miRNA regulators of hub genes identified through integrated bioinformatic analysis.

Gene	miRNA	TargetScan (Pct)	TargetScan (context++ score percentile)	miRDB (Target Score)	miRTarBase (Evidence)	Consensus (Count)
		> 0.9	> 90	> 90	Strong: Western Blot, qPCR (for the protein/mRNA level)	3 of 3
***CCL5***	**hsa-miR-146a-5p**	< 0.1	**99**	82	Luciferase reporter assay	3 of 3
*CCL5*	hsa-miR-146b-5p	< 0.1	**99**	82	NA	2 of 3
*CCL5*	hsa-miR-7153-5p	< 0.1	**99**	82	NA	2 of 3
***CXCR4***	**hsa-miR-9-5p**	0.59	**97**	78	**Strong: Immunohistochemistry//In situ hybridization//Luciferase reporter assay//qRT-PCR//Western blot**	**3 of 3**
***CXCR4***	**hsa-miR-139-5p**	< 0.1	**98**	**97**	**Strong: ChIP-seq//Flow//Immunohistochemistry//In situ hybridization//Luciferase reporter assay//Microarray//Northern blot//qRT-PCR//Western blot**	**3 of 3**
*CXCR4*	hsa-miR-520d-3p	0.58	93	74	NA	2 of 3
*CXCR4*	hsa-miR-520a-3p	0.58	92	74	NA	2 of 3
*CXCR4*	hsa-miR-520c-3p	0.58	91	74	NA	2 of 3
*CXCR4*	hsa-miR-373-3p	0.58	91	74	NA	2 of 3
*CXCR4*	hsa-miR-372-3p	0.58	90	74	NA	2 of 3
*CXCR4*	hsa-miR-613	0.13	98	71	NA	2 of 3
*CXCR4*	hsa-miR-1-3p	0.13	97	77	NA	2 of 3
*CXCR4*	hsa-miR-206	0.13	97	77	NA	2 of 3
***FOXP3***	**hsa-miR-34a-5p**	< 0.1	**94**	NA	**Microarray//qRT-PCR**	2 of 3
***MMP9***	**hsa-miR-149-5p**	NA	NA	55	**Strong: Luciferase reporter assay//qRT-PCR//Western blotting**	2 of 3
*TNFSF11*	hsa-miR-144-3p	0.19	**99**	**94**	NA	2 of 3
*TNFSF11*	hsa-miR-182-5p	0.15	97	67	NA	2 of 3
*TNFSF11*	hsa-miR-106b-5p	0.73	98	65	NA	2 of 3
*TNFSF11*	hsa-miR-526b-3p	0.73	97	65	NA	2 of 3
*TNFSF11*	hsa-miR-106a-5p	0.73	98	65	NA	2 of 3
*TNFSF11*	hsa-miR-20a-5p	0.73	97	65	NA	2 of 3
*TNFSF11*	hsa-miR-20b-5p	0.73	97	62	NA	2 of 3
*TNFSF11*	hsa-miR-93-5p	0.73	96	62	NA	2 of 3
*TNFSF11*	hsa-miR-17-5p	0.73	97	62	NA	2 of 3
*TNFSF11*	hsa-miR-519d-3p	0.73	97	62	NA	2 of 3
*TNFSF11*	hsa-miR-4262	0.11	92	51	NA	2 of 3
***TNFSF11***	**hsa-miR-18a-5p**	NA	NA	NA	**Strong: Microarray//Northern blot/ Luciferase reporter assay**	1 of 3
*TNFSF13b*	hsa-miR-195-5p	0.73	**98**	**96**	NA	2 of 3
*TNFSF13b*	hsa-miR-503-5p	0.64	**98**	68	NA	2 of 3
*TNFSF13b*	hsa-miR-15a-5p	0.79	**99**	**95**	NA	2 of 3
*TNFSF13b*	hsa-miR-497-5p	0.79	**99**	**93**	NA	2 of 3
*TNFSF13b*	hsa-miR-16-5p	0.79	**99**	**95**	NA	2 of 3
*TNFSF13b*	hsa-miR-424-5p	0.79	**99**	**93**	NA	2 of 3
*IL18*	hsa-miR-4770	< 0.1	**97**	58	NA	2 of 3
*IL18*	hsa-miR-143-3p	< 0.1	**97**	58	NA	2 of 3
*IL18*	hsa-miR-6088	< 0.1	**96**	56	NA	2 of 3
*IL18*	hsa-miR-4295	< 0.1	**90**	NA	NA	2 of 3
*IL18*	hsa-miR-129-5p	< 0.1	**97**	NA	NA	2 of 3
*IL18*	hsa-miR-23c	< 0.1	**99**	NA	NA	2 of 3
*IL18*	hsa-miR-23a-3p	< 0.1	**99**	NA	NA	2 of 3
*IL18*	hsa-miR-130a-5p	< 0.1	**99**	NA	NA	2 of 3
*IL18*	hsa-miR-23b-3p	< 0.1	**99**	NA	NA	2 of 3
***IL18***	**hsa-miR-130a-3p**	NA	NA	NA	**Strong: ELISA//Luciferase reporter assay//qRT-PCR**	1 of 3

### 3.9. Drug-gene interaction of hub genes

**Fig 8:**
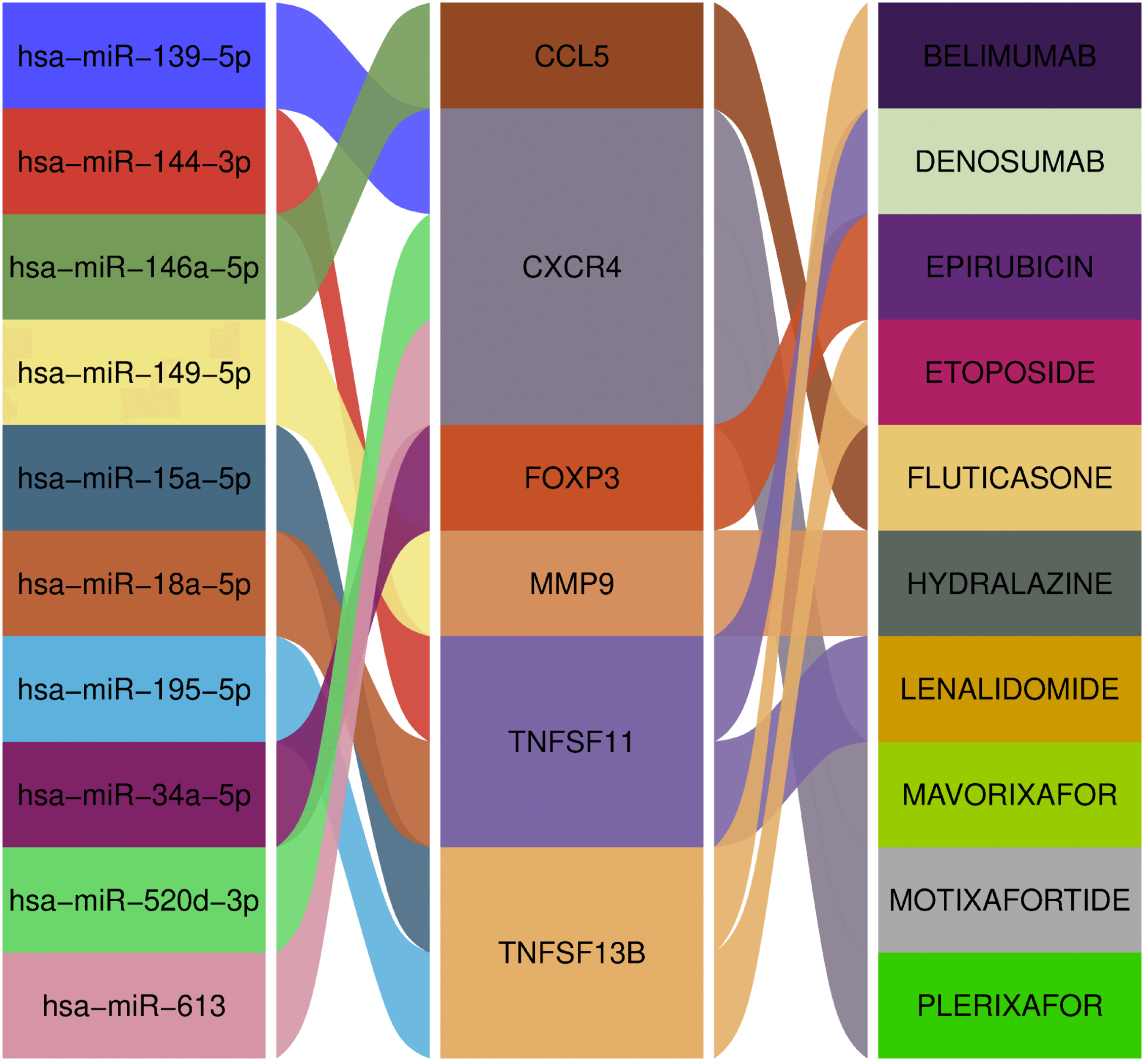
A comprehensive regulatory and drug-gene interaction network for diffuse gastric cancer (DGC). This integrated network depicts the identified hub genes (center), their upstream regulatory miRNAs, and FDA-approved drugs predicted to target them. The construction reveals potential post-transcriptional regulatory mechanisms and repurposable therapeutic strategies, highlighting the clinical translatability of the identified DGC hub genes.

**Fig 9:**
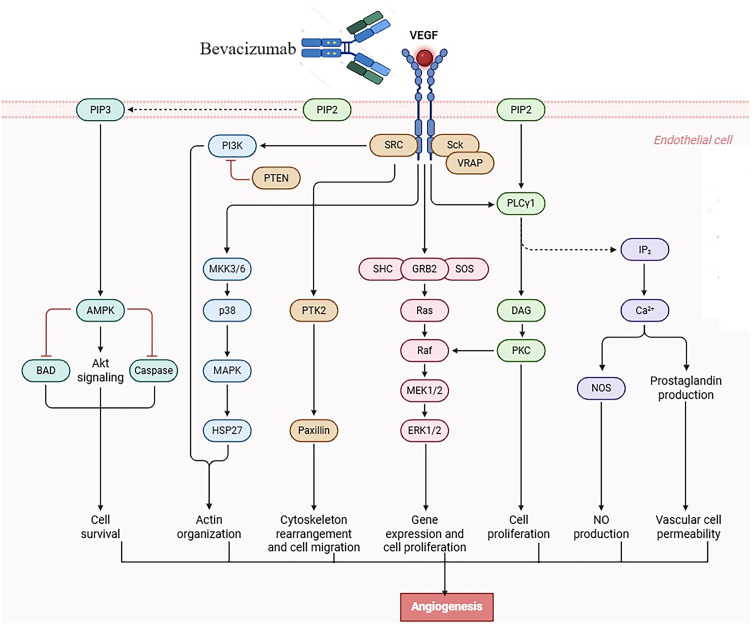
Mechanism of Bevacizumab-mediated suppression of the VEGF-A signaling pathway and its downstream effects on metastatic gene expression. The schematic illustrates how Bevacizumab, a monoclonal antibody targeting VEGF-A, inhibits VEGF-A/VEGFR-2 interaction, leading to suppression of PI3K/AKT and RAS/RAF/MEK/ERK signaling pathways. This ultimately reduces NF-κB activation and downregulates expression of pro-metastatic genes *CXCR4* and *MMP9*, thereby inhibiting tumor invasion and metastasis in gastric cancer. This schematic is based on a template provided by BioRender.com (https://www.biorender.com/).

Our computational drug repurposing analysis using the DGIdb database identified several FDA-approved drugs with high predicted affinity for key hub genes in DGC, revealing promising candidates for therapeutic repurposing (Fig 8, Table 4). Notably, the highest interaction scores were observed for Denosumab (an antiosteoporotic agent) with *TNFSF11* (score: 43.50) and Belimumab (an anti-inflammatory agent) with *TNFSF13B* (score: 32.63), suggesting a potent mechanistic link. Furthermore, the *CXCR4* axis emerged as a highly druggable target, with multiple approved antineoplastic agents like Mavorixafor (score: 5.22) and Plerixafor (score: 3.83) showing strong binding predictions. Other significant interactions included the antiinflammatory drug Fluticasone with *CCL5* (score: 2.75) and Lenalidomide with *TNFSF11* (score: 2.01), highlighting diverse pathways beyond conventional chemotherapy that could be exploited for novel treatment strategies in GC. Analysis of drug-gene interactions revealed that Bevacizumab (Avastin®) targets the regulatory network of both *CXCR4* and *MMP9*. The mechanism by which Bevacizumab suppresses VEGF-A pathway is illustrated in Fig 9. Finally, the full list of FDA‑approved drugs predicted to interact with the hub genes is presented in S9 Table.

**Table 4:** Clinically approved drugs predicted to interact with hub genes in gastric cancer, as identified by a computational drug repurposing analysis. Drugs are listed with their regulatory status, primary indication, and predicted interaction score.

Gene	Drug	Regulatory Approval	Indication	Interaction Score
*CXCR4*	Plerixafor	Approved	Antineoplastic agent	3.828
*CXCR4*	Cisplatin	Approved		0.014
*CXCR4*	Bevacizumab	Approved	Antineoplastic agent	0.104
*CXCR4*	Mavorixafor	Approved	HIV, antiviral agent	5.220
*CXCR4*	Motixafortide	Approved	Antineoplastic agent	3.132
*FOXP3*	Epirubicin	Approved	Antineoplastic Agents	0.725
*FOXP3*	Methotrexate	Approved	DMARD	0.475
*TNFSF11*	Denosumab	Approved	Antiosteoporotic agent	43.503
*TNFSF11*	Lenalidomide	Approved	Antineoplastic agent	2.008
*TNFSF13B*	Belimumab	Approved	DMARD, anti-inflammatory agent	32.627
*TNFSF13B*	Etoposide	Approved	Antineoplastic Agents	0.225
*CCL5*	Fluticasone	Approved	For treatment of symptomatic exophthalmos associated with thyroid-related eye disease, bronchodilator,glucocorticoid, Anti-inflammatory agent	2.748
*MMP9*	Celecoxib	Approved	NSAID	0.062
*MMP9*	Curcumin	Approved		0.032
*MMP9*	Hydralazine	Approved	Antihypertensive agent	0.209
*MMP9*	Bevacizumab	Approved	Antineoplastic agent	0.052
*MMP9*	Methyldopa Anhydrous	Approved	Antihypertensive agent	0.135
*MMP9*	Nifedipine	Approved	Antianginal agent Vasodilator agent	0.075

### 3.10. Prediction of transcription factors of Hub genes

Transcriptional regulatory network analysis using the TRRUST database identified several key transcription factors (TFs) as central regulators of the DGC hub gene landscape (Fig 10, Table 5). The TFs NFKB1 and RELA emerged as the most statistically significant master regulators (FDR = 1.57e-09 and 1.43e-07, respectively), demonstrating a complex regulatory profile by predominantly activating hub genes including *CCL5*, *CXCR4*, *FOXP3*, *IL18*, and *MMP9*. Notably, both TFs also exhibited a dual role by repressing *MMP9*. Further analysis defined a cohesive regulatory module wherein hub genes like *MMP9* and *CXCR4* are co-regulated by a suite of TFs. This intricate network pinpoints NF-κB signaling and MMP9 regulation as critical, multifaceted nodes in DGC pathogenesis.

**Table 5:** Regulatory network analysis of key transcription factors and their target hub genes in diffuse gastric cancer. The table summarizes significant transcription factor-hub gene interactions, including functional descriptions of each transcription factor, regulated target genes, supporting literature references (PubMed ID), and statistical significance values (P-value and FDR) for the identified interactions.

#	Key TF	Description	Genes	References (PMID)	P value	FDR
1	NFKB1	nuclear factor of kappa light polypeptide gene enhancer in B-cells 1	*CCL5* (Unknown)	11259372	1.12e-10	1.57e-09
			*CXCR4* (Unknown)	20157762		
			*IL18* (Unknown)	10227974		
			*MMP9* (Unknown)	11376631		
			*TNFSF13B* (Unknown)	16497967		
			*CCL5* (Activation)	11478739		
			*CXCR4* (Activation)	12690099		
			*FOXP3* (Activation)	20462637		
			*IL18* (Activation)	15963597		
			*MMP9* (Activation)	10359016		
			*MMP9* (Repression)	15605368		
2	RELA	v-rel reticuloendotheliosis viral oncogene homolog A (avian)	*CCL5* (Unknown)	11259372	2.04e-08	1.43e-07
			*CXCR4* (Unknown)	20157762		
			*FOXP3* (Unknown)	23178569		
			*IL18* (Unknown)	10227974		
			*MMP9* (Unknown)	11376631		
			*CCL5* (Activation)	11478739		
			*CXCR4* (Activation)	12690099		
			*FOXP3* (Activation)	20462637		
			*IL18* (Activation)	15963597		
			*MMP9* (Activation)	10359016		
			*MMP9* (Repression)	15086314		
3	IRF1	interferon regulatory factor 1	*FOXP3* (Repression)	18641303	6.46e-07	3.01e-06
			*CCL5* (Unknown)	10385645		
			*MMP9* (Unknown)	12105194		
4	STAT1	signal transducer and activator of transcription 1, 91kDa	*FOXP3* (Unknown)	19124747	2.94e-06	1.03e-05
			*TNFSF13B* (Unknown)	23271704		
			*MMP9* (Repression)	19965686		
5	KLF5	Kruppel-like factor 5 (intestinal)	*CXCR4* (Activation)	19460858	9.18e-06	2.5e-05
			*MMP9* (Activation)	18617520		
6	SNAI2	snail homolog 2 (Drosophila)	*CXCR4* (Activation)	22074556	1.07e-05	2.5e-05
			*MMP9* (Activation)	22074556		
7	NFKBIA	nuclear factor of kappa light polypeptide gene enhancer in B-cells inhibitor, alpha	*CCL5* (Repression)	15228586	2.01e-05	4.02e-05
			*MMP9* (Unknown)	11376631		
8	SMAD3	SMAD family member 3	*FOXP3* (Activation)	20937549	5.46e-05	9.55e-05
			*MMP9* (Repression)	15086314		
9	SIRT1	sirtuin 1	*MMP9* (Repression)	19376817	0.000132	0.000205
			*FOXP3* (Unknown)	21533107		
10	SPI1	spleen focus forming virus (SFFV) proviral integration oncogene spi1	*CCL5* (Unknown)	10760799	0.000221	0.000309
			*IL18* (Unknown)	14991611		
11	ETS1	Avian Erythroblastosis Virus E26 (V-Ets) Oncogene Homolog-1 V-Ets Avian Erythroblastosis Virus E26 Oncogene Homolog 1	*MMP9* (Activation)	22270366	0.000359	0.000456
			*CXCR4* (Unknown)	21741415		
			*MMP9* (Unknown)	10949659		
12	CREB1	cAMP responsive element binding protein 1	*CCL5* (Unknown)	11413310	0.000465	0.000543
			*CXCR4* (Unknown)	11874984		
13	JUN	jun proto-oncogene	*MMP9* (Activation)	15777790	0.00127	0.00136
			*MMP9* (Repression)	21474819		
			*MMP9* (Unknown)	10935492		
			*CCL5* (Unknown)	11413310		
14	SP1	Sp1 transcription factor	*MMP9* (Activation)	19616522	0.0121	0.0121
			*CCL5* (Unknown)	11259372		
			*MMP9* (Unknown)	11060311		

**Fig 10:**
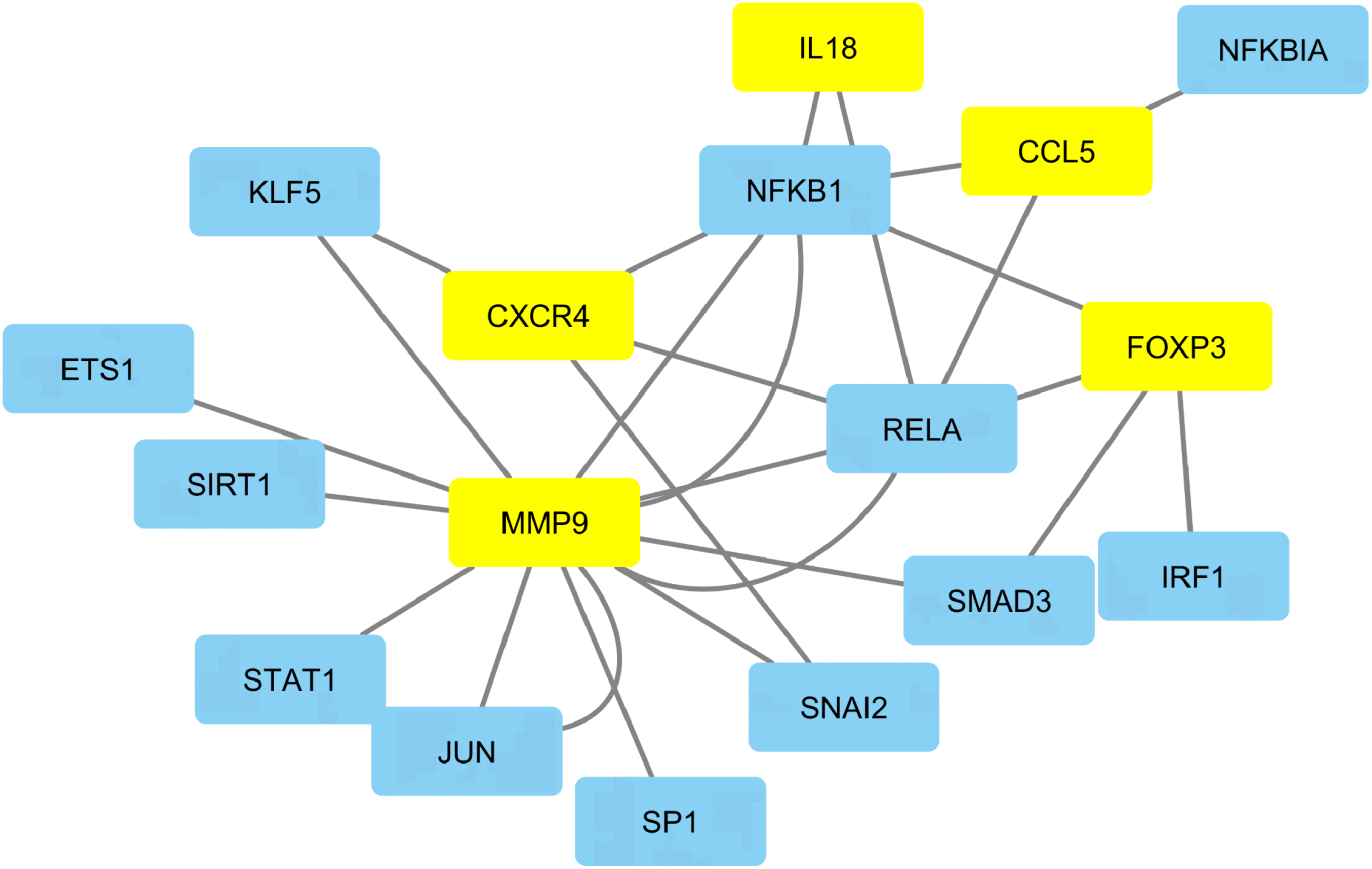
Master transcriptional regulators of the diffuse gastric cancer hub gene network. This network, constructed using the TRRUST database, reveals the key transcription factors (blue nodes) that potentially govern the expression of the identified hub genes (yellow nodes). The structure illustrates a hierarchical regulatory framework underlying DGC pathogenesis, pinpointing central TFs as potential master regulators.

## 4. Discussion

Gastric cancer (GC) is a critical global health burden, with projections indicating a dramatic rise in incidence and mortality in Asian countries [53]. The diffuse subtype (DGC), which constitutes approximately 30% of cases, is particularly aggressive, afflicts younger patients, and suffers from a lack of specific biomarkers for early detection and prognosis [54,55]. To address this diagnostic gap, this study leverages the power of machine learning (ML) to analyze RNA-seq data and identify key drivers of DGC pathogenesis. We analyzed six DGC tumors and six normal gastric tissue samples from the GEO database, processing 21,542 genes. The ReliefF algorithm, a feature selection method proven effective in prior oncological studies for prioritizing informative genetic features, was applied to this dataset [56,57]. The goal of this approach is to elucidate critical cancer-related pathways and identify a robust gene signature with potential as a diagnostic or prognostic biomarker for DGC.

The integration of GO and KEGG pathway analyses revealed that upregulated genes in our DGC dataset are critically involved in intermediate filament organization, telomere maintenance, ATP-responsive mechanisms, and cell cycle regulation, collectively illuminating the molecular basis of DGC’s aggressive phenotype. Specifically, the enrichment of intermediate filament organization, particularly through vimentin overexpression, strongly underscores the role of epithelial-mesenchymal transition (EMT) in promoting the invasive and migratory characteristics hallmark of DGC [58–61]. Concurrently, upregulated telomere maintenance genes, such as *TERT*, likely confer cellular immortality, enabling the relentless proliferation observed in these tumors [62,63]. Furthermore, the involvement of ATP-responsive mechanisms points to a dual role for extracellular ATP (eATP) in both activating purinergic signaling pathways to drive proliferation and invasion, and in contributing to an immunosuppressive tumor microenvironment via its conversion to adenosine [64–69]. Furthermore, eATP contributes to cancer therapy resistance by upregulating ATP-binding cassette (ABC) transporters, enhancing their drug-efflux capacity and promoting multidrug resistance [70,71]. Finally, the dysregulation of cell cycle checkpoints facilitates the uncontrolled mitotic division central to tumor growth [72]. Together, these pathways are not isolated events but likely form an interconnected network that drives the pathogenesis and therapeutic resistance of DGC, with vimentin-mediated EMT potentially serving as a cornerstone of its diffuse and invasive nature.

In contrast, the analysis of downregulated genes revealed significant enrichment in processes related to immune response, lipid metabolism, and fatty acid biosynthesis, collectively pointing to key hallmarks of DGC progression. The suppression of immune-related pathways aligns with an immunosuppressive tumor microenvironment, a known facilitator of immune evasion and tumor growth [73,74]. Simultaneously, we observed a paradoxical downregulation of de novo lipid and fatty acid biosynthesis genes, despite the high lipid demand required for the aggressive proliferation of DGC cells [75,76]. This finding underscores a critical metabolic rewiring, suggesting a shift in DGC tumors away from energy-intensive internal lipid synthesis and towards the more efficient scavenging of exogenous lipids from the tumor microenvironment [77]. Furthermore, the specific downregulation of genes such as *AKR1C3* (Aldo-Keto Reductase Family 1 Member C3), *HPGD* (15-Hydroxyprostaglandin Dehydrogenase), and *SMPD3* (Sphingomyelin Phosphodiesterase 3 or Neutral Sphingomyelinase 2) suggests a selective pressure to suppress their potential tumor-suppressive functions, including the inactivation of oncogenic signaling molecules and the induction of ceramide-mediated apoptosis [78–84]. Therefore, the observed transcriptional profile does not indicate a diminished role for lipids in DGC pathogenesis, but rather a sophisticated adaptation favoring lipid uptake and repurposing, while silencing metabolic checkpoints that could otherwise constrain tumor survival and progression.

KEGG pathway analysis of differentially expressed genes revealed distinct enrichments that illuminate key biological alterations in DGC. The most compelling insights emerged from the significant enrichment of oxidative phosphorylation (OXPHOS) among downregulated genes. This finding indicates fundamental metabolic reprogramming in DGC cells, characterized by a shift away from mitochondrial energy production. This is consistent with the Warburg effect, a hallmark of cancer metabolism where cells preferentially utilize glycolysis over OXPHOS, even under oxygen-rich conditions, to fuel rapid proliferation and anabolic growth [85]. According to the feature selection method results, OXPHOS-related genes that also showed statistically significant down-regulation were as follows: complex I (*NDUFA1, NDUFA2, NDUFV3*), complex II (*SDHA, SDHD*), complex III (*UQCRC1, UQCRC2*), complex IV (*COX5A, COX6B1, COX7A2*), and complex V (*ATP5F1A, ATP5PB, ATP5MG*). However, the feature selection method revealed that expression of some other proteins, such as SDHB (*p* = 0.06), SDHC (*p* = 0.31) in complex II, as well as CYC1(*p* = 0.19) in complex III, is decreased, but the statistical analysis of the CLC didn’t support them. Furthermore, the concomitant downregulation of the ‘Chemical carcinogenesis–reactive oxygen species’ pathway suggests a dual purpose for this metabolic adaptation. By reducing OXPHOS activity, DGC cells may not only meet their anabolic needs but also limit the production of mitochondrial reactive oxygen species (ROS), thereby evading ROS-induced cell death and promoting tumor cell survival [86]. This collective downregulation underscores a sophisticated metabolic strategy that supports both the biosynthetic and survival needs of DGC.

Network analysis identified the initial seven hub genes exhibiting high connectivity, *CCL5, CXCR4, MMP9, FOXP3, IL18, TNFSF11*, and *TNFSF13B*, a finding consistent with previous research. The specific roles of these genes are discussed below. Subsequent statistical validation using Baggerley’s test within the CLC software confirmed that *CXCR4* (*p* = 0.014) and *TNFSF13B* (*p* < 0.000001) were individually significant (Table 1).

*CXCR4*, a G-protein-coupled receptor (GPCR), is a key regulator of fundamental biological processes throughout organogenesis, hematopoiesis, and immune responses. The CXCR4/CXCL12 (SDF-1) axis is a well-established regulator of cell migration, playing a physiological role in stem cell homing and a pathological one in directing cancer stem cells to CXCL12-rich metastatic niches, including lymph nodes, bone marrow, liver, and lungs [87]. In GC, *CXCR4* overexpression is frequently observed and is often correlated with advanced disease and a poor prognosis, a finding confirmed by our study through both feature selection and statistical validation [88–92]. Notably, its expression appears particularly relevant to the diffuse (DGC) subtype, where it has been linked to more aggressive clinical behavior [89]. However, the relationship between *CXCR4* and specific clinical parameters such as TNM stage and Lauren classification remains a subject of debate, with some studies reporting conflicting associations [88,93–97]. These discrepancies may stem from methodological variations, tumor heterogeneity, or the influence of other components within the tumor microenvironment, suggesting that the functional impact of *CXCR4* is highly context dependent. Nonetheless, its confirmed upregulation in our DGC cohort solidifies its role as a key hub gene and a potential therapeutic target in this aggressive subtype.

Alongside *CXCR4*, *TNFSF13B* (also known as BAFF or BLyS) emerged as a significantly upregulated hub gene in GC, demonstrating statistically significant overexpression compared to normal controls (p < 0.000001). While well-characterized as a key immunomodulatory cytokine that promotes B cell proliferation and differentiation in hematological malignancies, its specific role in solid tumors like GC remains an emerging area of research [98–101]. In other cancers, TNFSF13B drives oncogenesis through B cell-dependent immune evasion, tumor progression, and inhibition of apoptosis [102]. Therefore, our robust identification of *TNFSF13B* as a central hub gene in GC provides a strong rationale to investigate these established oncogenic mechanisms within the gastric tumor microenvironment, positioning it as a promising and previously underappreciated therapeutic target for this disease.

MMP9, a zinc-dependent matrix metalloproteinase, plays a critical role in GC progression and metastasis by degrading extracellular matrix components, notably type IV collagen in basement membranes [103,104]. Its expression is influenced by factors such as *H. pylori* infection and specific promoter polymorphisms like the -1562C/T variant, which elevates its mRNA and protein levels [105]. Functionally, inhibiting MMP9 has been shown to suppress distant metastasis in GC through an MMP-9/PI3K/AKT/Snail-dependent pathway, underscoring its therapeutic relevance [106]. In our analysis, *MMP9* exhibited the most pronounced overexpression (fold change = 16.6) among all hub genes, yet this did not reach statistical significance (p = 0.34) (Table 1). This discrepancy between a large effect size and a non-significant p-value is a well-documented phenomenon, often attributed to factors such as limited sample size, technical variation, or tumor heterogeneity, rather than a lack of biological relevance. For example, the substantial fold change, consistent with extensive research linking *MMP9* overexpression to advanced GC stage and poor prognosis, solidifies its position as a biologically pivotal node in DGC progression, warranting further investigation in larger cohorts [107,108]. The ReliefF algorithm, which captures multivariate relationships, prioritized *MMP9* as a top feature despite the non-significant univariate p-value, highlighting the complementary value of machine learning approaches in small-sample transcriptomic studies.

Despite its high connectivity, *CCL5* was not individually statistically significant in our cohort; however, its central network position is strongly supported by extensive literature delineating its multifaceted role in GC aggressiveness. Elevated *CCL5* expression is consistently correlated with advanced tumor stage, metastasis, chemoresistance, and poor patient prognosis [109–113]. Mechanistically, the CCL5/CCR5 axis promotes tumor proliferation by activating oncogenic pathways like PI3K/AKT and NF-κB, leading to the upregulation of cell cycle regulators such as cyclin D1 and c-Myc [114]. Concurrently, this signaling enhances cell motility by facilitating F-actin polymerization, thereby driving invasion [115]. Beyond direct tumorigenic effects, *CCL5* orchestrates a potent immunosuppressive response. It has been shown to selectively induce Fas-FasL-mediated apoptosis in CD8 + T cells, effectively blunting the cytotoxic immune response and facilitating tumor immune evasion [116]. Thus, *CCL5* emerges as a pleiotropic driver in GC, simultaneously promoting tumor growth, metastatic dissemination, and an immunosuppressive tumor microenvironment.

The upregulation of *FOXP3*, a master regulator of regulatory T cells (Tregs), represents another key immunosuppressive mechanism in our DGC network. High levels of *FOXP3* ⁺ Tregs within the tumor microenvironment are an established marker of poor prognosis, significantly correlating with reduced overall survival and increased lymph node metastasis in GC [117]. Furthermore, *FOXP3* expression is not confined to Tregs and can be ectopically expressed by tumor cells themselves, potentially enabling them to directly suppress anti-tumor immunity [118]. The recruitment of these suppressive cells is facilitated, in part, by the CCL5/CCR5 axis, another hub in our network, which promotes the selective migration of Tregs into the tumor, creating a reinforced immunosuppressive circuit that disrupting this axis can inhibit [119]. Therefore, *FOXP3* upregulation is integral to the immunosuppressive landscape of DGC, working in concert with other hub genes like *CCL5* to facilitate immune evasion and tumor progression.

TNFSF11, also known as RANKL, is a member of the TNF superfamily best characterized for its central role in the RANKL-RANK-osteoprotegerin (OPG) signaling axis, a critical pathway driving osteoclast differentiation and activation [120]. Beyond its role in bone biology, RANKL is ubiquitously expressed and triggers diverse physiological and inflammatory responses upon binding its receptor RANK [121]. While the expression and function of the RANKL/RANK axis in GC cells remain poorly characterized, a genetic association has been demonstrated between a *TNFSF11* gene variant (rs9533156) and increased GC risk [122]. In this context, our observed upregulation of *TNFSF11* in GC tissues suggests a potential, yet uncharacterized, role for this pathway in gastric tumorigenesis. This novel finding warrants further validation in larger cohorts and functional investigation to elucidate whether RANKL signaling contributes to GC progression through mechanisms such as modulating the tumor microenvironment or influencing cell survival.

Although GEPIA analysis indicated upregulated expression across all identified hub genes, our results consistently demonstrated the downregulation of *IL18* (Fig 4, Table 1). Therefore, *IL18* was the sole hub gene in our analysis to be downregulated in DGC tissues, a finding that presents a compelling paradox given its complex, dual role in tumor biology. While IL18, a pro-inflammatory cytokine, can promote tumor progression through angiogenesis, invasion, and immune suppression, it also possesses potent anti-tumor activities, including the enhancement of natural killer cell (NK) cytotoxicity and the induction of apoptosis [123–128] Its downregulation in our DGC cohort suggests that the net effect of *IL18* in this specific subtype may be anti-tumorigenic, and its loss could therefore represent a novel immune evasion mechanism. While IL18’s role in *H. pylori*-associated intestinal GC (IGC) has been established, its function in DGC has remained largely unexplored [123]. Our identification of *IL18* as a downregulated hub gene provides the first preliminary evidence of its potential role as a tumor-suppressive factor in the pathogenesis of DGC, warranting further functional validation.

Prognostic evaluation via Kaplan-Meier overall survival (OS) analysis further suggested CXCR4 as a potential prognostic indicator, where elevated expression was associated with a 1.5-fold increase in mortality risk (Hazard Ratio [HR] = 1.5, p < 0.01), underscoring its pivotal role in GC aggressiveness (Fig 5). However, multivariable analysis and validation in independent cohorts are necessary before it can be considered a true biomarker. In contrast, the expression levels of the other six hub genes did not demonstrate statistically significant correlations with patient survival (p > 0.05).

The final selection of *CXCR4*, *MMP9*, and *TNFSF13B* as prioritized core hub genes was driven by their statistical significance, elevated expression profiles, and complementary functional roles in DGC pathogenesis. *CXCR4* and *TNFSF13B* were prioritized based on significant differential expression, mediating metastasis and a novel immunomodulatory axis, respectively, while *MMP9* was included due to its significant overexpression, underscoring its critical role in extracellular matrix remodeling. These genes operate within an NF-κB-coordinated network, where *CXCR4* promotes cellular migration, MMP9 facilitates tissue invasion, and *TNFSF13B* regulates immune responses. Notably, the VEGF-A inhibitor Bevacizumab (Avastin®) indirectly targets both *MMP9* and *CXCR4* by suppressing the VEGF-induced PI3K/AKT and RAS/RAF/MEK/ERK signaling cascades, thereby inhibiting NF-κB-mediated transcriptional activation of these pro-metastatic genes [129–132]. This integrated module not only elucidates key mechanisms of DGC progression but also highlights actionable therapeutic strategies,from VEGF pathway inhibition to novel immunomodulation via *TNFSF13B*, offering a multi-dimensional approach for targeted intervention.

Finally, it is worth noticing that ML approaches demonstrated acceptable performance in identifying biologically relevant DEGs in DGC independent of conventional *p*-value or fold-change thresholds. Conventional methods overlook genes that lack statistical significance (e.g., *MMP9* with fold-change = 16.6 but *p* = 0.34), while ML captures subtle but functionally relevant expression patterns by learning multidimensional relationships between gene networks. This sensitivity derives from ML’s capacity to: (i) integrate multi-omics datasets to detect combined regulators, (ii) eschew stringent statistical thresholds that dismiss context-dependent regulators, and (iii) capture nonlinear interactions in shared heterogeneous tumors, enabling detection of “invisible” mediators like Class II cancer genes (*TNFSF13B*). Though biological validation (e.g., siRNA) is still necessary to establish causality, ML surpasses reductionist analyses for exploratory biomarker discovery by its ability to unravel weak-but-concerted signals driving GC pathogenesis. We thus suggest hybrid pipelines that combine ML-based prioritization with network biology tools (e.g., KeyPathwayMiner) and experimental filtering to accelerate precision oncology in GC.

While our pilot study successfully demonstrates the feasibility of integrating machine learning with transcriptomic data for DGC analysis, the small sample size (n = 6 per group) imposes important limitations, and our findings should be considered hypothesis-generating rather than definitive. Therefore, we strongly encourage future studies with larger, independent cohorts to validate the candidate genes and pathways identified here and to transition from hypothesis generation to definitive predictive modeling. In addition, all findings presented here require independent validation through in vitro (*e.g*., qPCR, Western blot, functional assays) and in vivo studies before any clinical translation. We also acknowledge the absence of experimental validation. While our bioinformatic predictions are supported by multiple lines of evidence (literature, network analysis, and public database validation), definitive proof of causality requires targeted in vitro experiments such as gene knockdown/overexpression studies, migration/invasion assays, and immunohistochemical confirmation at the protein level.

## 5. Conclusions

In conclusion, this pilot study demonstrates the feasibility and utility of integrating RNA-seq data with ML-based feature selection to explore the molecular biology of young-onset DGC, having utilized all available RNA-seq samples from patients under 40 years of age. Our analytical framework provides a focused, high-priority set of candidate genes and pathways, nominating *CXCR4*, *MMP9* and *TNFSF13B* as key therapeutic targets and validating ML’s power for uncovering important genes that traditional statistics may overlook. Although definitive confirmation in a larger, independent cohort is an essential next step, this work provides a powerful methodological foundation and valuable insights. We believe this pilot study will be a valuable resource for guiding future research, and its findings are poised for significant improvement with increased sample sizes, paving the way for more precise diagnostic and therapeutic strategies in this aggressive cancer.

### Highlights

1. **Machine learning identifies a prioritized core hub gene network** (*CXCR4, MMP9, TNFSF13B*) driving diffuse gastric cancer (DGC) pathogenesis, revealing key drivers of metastasis, immune evasion, and extracellular matrix remodeling.
2. **Upregulated pathways** (EMT, telomere maintenance, ATP signaling, cell cycle) and **downregulated pathways** (immune response, OXPHOS, lipid biosynthesis) delineate the aggressive and metabolically rewired phenotype of DGC.
3. **The VEGF inhibitor Bevacizumab indirectly targets *CXCR4* and *MMP9***, providing a therapeutic rationale for its use in DGC by suppressing a central NF-κB-coordinated pro-metastatic network.
4. ***TNFSF13B* (BAFF) is a highly significant immunomodulatory hub gene** with exceptional therapeutic potential, positioning Belimumab as a promising repurposing candidate for DGC treatment.
5. **Machine learning surpasses conventional statistics** by identifying high-impact genes like *MMP9* (high fold-change, low p-value) and *TNFSF13B*, advocating for hybrid ML-network biology pipelines in precision oncology.

## Supporting information

S1 Fig Principal Component Analysis (PCA) of the combined RNA-seq datasets GSE113255 and GSE122401. PCA was performed on the normalized expression matrix of all 12 samples (6 normal and 6 tumor gastric tissues) from two independent GEO datasets. Samples are colored by tissue type (Normal: blue; Tumor: red) and shaped by dataset origin (GSE113255: circles; GSE122401: triangles).
(TIF)

S2 Fig Gene Ontology (GO) enrichment analysis of Biological Processes (BP) for up- and down-regulated genes in diffuse gastric cancer. The integrated Sankey–dot plot illustrates the relationship between significantly enriched BP terms and their associated differentially expressed genes. (A) Enriched BP terms for up-regulated genes. (B) Enriched BP terms for down-regulated genes. The Sankey diagram (left) connects each GO term to the corresponding genes, with node width proportional to the number of genes annotated to each term. The dot plot (right) visualizes the statistical significance (–log₁₀(p-value)) and gene count (dot size) for each enriched term. Only the significant terms (p-value < 0.05) are displayed.
(TIF)

S3 Fig Gene Ontology (GO) enrichment analysis of Molecular Functions (MF) for up- and down-regulated genes in diffuse gastric cancer. The combined Sankey–dot plot depicts the functional annotations of differentially expressed genes based on their molecular activities. (A) MF terms associated with up-regulated genes. (B) MF terms associated with down-regulated genes. The Sankey diagram (left) connects each GO term to the corresponding genes, with node width proportional to the number of genes annotated to each term. The dot plot (right) visualizes the statistical significance (–log₁₀(p-value)) and gene count (dot size) for each enriched term. Only the significant terms (p-value < 0.05) are displayed.
(TIF)

S4 Fig Gene Ontology (GO) enrichment analysis of Cellular Components (CC) for up- and down-regulated genes in diffuse gastric cancer. The Sankey–dot plot provides a comprehensive overview of the subcellular localization of differentially expressed gene products. (A) CC terms enriched among up-regulated genes. (B) CC terms enriched among down-regulated genes. The Sankey diagram (left) connects each GO term to the corresponding genes, with node width proportional to the number of genes annotated to each term. The dot plot (right) visualizes the statistical significance (–log₁₀(p-value)) and gene count (dot size) for each enriched term. Only the significant terms (p-value < 0.05) are displayed.
(TIF)

S5 Fig KEGG pathway enrichment analysis of up- and down-regulated genes in diffuse gastric cancer. The dot plot displays significantly enriched pathways (p-value < 0.05) for upregulated (red) and downregulated (green) genes. The dot size represents the number of DEGs mapped to each pathway.
(TIF)

S6 Fig Gene Ontology (GO) enrichment analysis of the identified hub genes in diffuse gastric cancer. The integrated Sankey–dot plot illustrates the functional annotations of the seven network-derived hub genes (CCL5, CXCR4, FOXP3, IL18, MMP9, TNFSF11, and TNFSF13B) across the three GO categories: (A) Biological Process (BP), (B) Molecular Function (MF), and (C) Cellular Component (CC). The Sankey diagram (left) connects each hub gene to its associated GO terms, with edge width proportional to the number of annotations and node size reflecting the number of genes mapped to each term. The dot plot (right) displays the enrichment significance (–log₁₀(p-value)) and gene count (dot size) for each significantly enriched term (p-value < 0.05).
(TIF)

S1 Table Cohort description of the exploratory RNA-seq analysis in young-onset diffuse gastric cancer. This table documents the most important data for the 12-sample pilot cohort of 6 DGC tumors and 6 normal tissues from datasets GSE113255 and GSE122401. N/A: Not available in GEO database.
(DOCX)

S2 Table Python code for ReliefF feature selection analysis. https://github.com/sjsajadi/UncoveringtheMolecularLandscapeofYoungOnsetDiffuseGastric.git
(DOCX)

S3 Table The top 50 most informative upregulated and downregulated genes identified by the RReliefF feature selection algorithm. (DOCX)

S4 Table CLC Genomics Workbench analysis results. (XLSX)

S5 Table ReliefF-based feature selection analysis results. (CSV)

S6 Table GO and KEGG enrichment analysis results of DEGs and hub genes. (XLSX)

S7 Table Immune cell infiltration analysis of hub genes. (ZIP)

S8 Table miRNA regulatory network analysis results of hub genes (ZIP)

S9 Table Drug-gene interaction analysis results. (XLSX)

## Abbreviations

Bp: Biological processes
CC: Cellular component
DEGs: Differentially expressed genes
ECM: Extracellular matrix
EMT: Epithelial-mesenchymal transition
FS: Feature selection
GC: Gastric cancer
GEPIA: Gene Expression Profiling Interactive Analysis
GO: Gene Ontology
HR: Hazard ratio
IGC: Intestinal gastric cancer
KEGG: Kyoto Encyclopedia of Genes and Genomes
MCC: Maximal Clique Centrality
MF: Molecular function
MMPs: Matrix metalloproteinases
ML: Machine learning
OS: Overall survival
OXPHOS: Oxidative phosphorylation
PPI: Protein-protein interaction
RNA-seq: RNA sequencing
TCGA: The Cancer Genome Atlas
STAD: Stomach Adenocarcinoma
