# Supplementary material for: Uncovering the molecular landscape of young-onset diffuse gastric cancer: A relieff-based feature selection analysis on RNA-Seq data
Source: PLoS One. 2026 Aug 6;21(8):e0354961. doi: 10.1371/journal.pone.0354961 (PMC13446702; doi:10.1371/journal.pone.0354961)
Supplement: Supplementary file 1 [file pone.0354961.s007.docx]

**S1 Table. Cohort description of the exploratory RNA-seq analysis in young-onset diffuse gastric cancer.** This table documents the most important data for the 12-sample pilot cohort of 6 DGC tumors and 6 normal tissues from datasets GSE113255 and GSE122401. N/A: Not available in GEO database.

| No. | GSE ID | GSM ID | SRA ID | Sample Type | Histology | Biospecimen type | Age | Sex | Library  Layout |
| --- | --- | --- | --- | --- | --- | --- | --- | --- | --- |
| 1 | GSE113255 | GSM3101070 | SRR7014300 | Normal | Normal mucosae | Tissue | N/A | N/A | Paired‐end |
| 2 | GSE113255 | GSM3101072 | SRR7014302 | Normal | Normal mucosae | Tissue | N/A | N/A | Paired‐end |
| 3 | GSE113255 | GSM3101068 | SRR7014298 | Normal | Normal mucosae | Tissue | N/A | N/A | Paired‐end |
| 4 | GSE122401 | GSM3501782 | SRR8281317 | Normal | Normal mucosae | Tissue | 33 | Male | Paired‐end |
| 5 | GSE122401 | GSM3501783 | SRR8281318 | Normal | Normal mucosae | Tissue | 29 | Male | Paired‐end |
| 6 | GSE122401 | GSM3501784 | SRR8281319 | Normal | Normal mucosae | Tissue | 30 | Female | Paired‐end |
| 7 | GSE113255 | GSM3101104 | SRR7014334 | Tumor | Diffuse Type | Tissue | 34 | Male | Paired‐end |
| 8 | GSE113255 | GSM3101117 | SRR7014347 | Tumor | Diffuse Type | Tissue | 25 | Female | Paired‐end |
| 9 | GSE113255 | GSM3101139 | SRR7014369 | Tumor | Diffuse Type | Tissue | 35 | Male | Paired‐end |
| 10 | GSE122401 | GSM3501862 | SRR8281397 | Tumor | Diffuse Type | Tissue | 33 | Male | Paired‐end |
| 11 | GSE122401 | GSM3501863 | SRR8281398 | Tumor | Diffuse Type | Tissue | 29 | Male | Paired‐end |
| 12 | GSE122401 | GSM3501864 | SRR8281399 | Tumor | Diffuse Type | Tissue | 30 | Female | Paired‐end |

GSE113255: https://www.ncbi.nlm.nih.gov/geo/query/acc.cgi?acc=GSE113255

GSE122401: https://www.ncbi.nlm.nih.gov/geo/query/acc.cgi?acc=GSE122401
