# Supplementary material for: Uncovering the molecular landscape of young-onset diffuse gastric cancer: A relieff-based feature selection analysis on RNA-Seq data
Source: PLoS One. 2026 Aug 6;21(8):e0354961. doi: 10.1371/journal.pone.0354961 (PMC13446702; doi:10.1371/journal.pone.0354961)
Supplement: Supplementary file 2 [file pone.0354961.s008.docx]

\# -*- coding: utf-8 -*-

"""code.ipynb

Automatically generated by Colab.

Original file is located at

https://colab.research.google.com/drive/1mB83KRWisZ9ZlqhzgEuhYvXWt2oGRiJJ

"""

\# =============================================================================

\# Feature Selection for Gastric Cancer Transcriptomic Data

\# Based on ReliefF Algorithm - Corrected Version

\# =============================================================================

\# Install necessary libraries

!pip install scikit-learn pandas numpy skrebate optuna boruta joblib

\# Import required libraries

import pandas as pd

from sklearn.feature_selection import mutual_info_classif, f_classif

from sklearn.ensemble import RandomForestClassifier

from sklearn.model_selection import cross_val_score, LeaveOneOut

from sklearn.metrics import accuracy_score, classification_report, confusion_matrix

from sklearn.preprocessing import StandardScaler

from skrebate import ReliefF

import warnings

warnings.filterwarnings("ignore")

import csv

import os

from google.colab import drive

SEED = 42

import os

import random

os.environ["PYTHONHASHSEED"] = str(SEED)

random.seed(SEED)

import numpy as np

np.random.seed(SEED)

\# =============================================================================

\# 1. DATA LOADING AND PREPROCESSING

\# =============================================================================

\# Mount Google Drive

drive.mount('/content/drive')

\# Transpose CSV file if needed (row = samples, columns = genes)

def transpose_csv(input_filename, output_filename, delimiter=","):

try:

with open(input_filename, 'r', newline='', encoding='utf-8') as infile:

reader = csv.reader(infile, delimiter=delimiter)

data = list(reader)

transposed_data = list(zip(*data))

with open(output_filename, 'w', newline='', encoding='utf-8') as outfile:

writer = csv.writer(outfile, delimiter=delimiter)

writer.writerows(transposed_data)

print(f"CSV file transposed and saved as '{output_filename}'")

except FileNotFoundError:

print(f"Error: Input file '{input_filename}' not found.")

except Exception as e:

print(f"An error occurred: {e}")

\# Transpose data if necessary (samples as rows, genes as columns)

transpose_csv("/content/drive/My Drive/Revised_ReliefF_Algorithm_Feature_Selection_for_Gastric_Cancer_Transcriptomic_Data/data/raw/data.csv",

"/content/drive/My Drive/Revised_ReliefF_Algorithm_Feature_Selection_for_Gastric_Cancer_Transcriptomic_Data/data/processed/data_transposed.csv")

\# Load the dataset

file_path = '/content/drive/My Drive/Revised_ReliefF_Algorithm_Feature_Selection_for_Gastric_Cancer_Transcriptomic_Data/data/processed/data_transposed.csv'

data = pd.read_csv(file_path)

print("Dataset shape:", data.shape)

\# =============================================================================

\# 2. FEATURE AND TARGET SEPARATION

\# =============================================================================

\# Last column is the target variable (tumor vs normal)

X = data.iloc[:, :-1].values # Features (all genes)

y = data.iloc[:, -1].values	# Target (class labels)

print(f"Number of features (genes): {X.shape[1]}")

print(f"Number of samples: {X.shape[0]}")

\# =============================================================================

\# 3. FEATURE SELECTION USING RELIEFF (CORRECTED)

\# =============================================================================

def feature_selection_relieff(X, y, n_neighbors=10, num_features=1260):

"""

Feature selection using ReliefF algorithm.

Parameters:

-----------

X : array-like, shape (n_samples, n_features)

Feature matrix

y : array-like, shape (n_samples,)

Target labels

n_neighbors : int, default=10

Number of nearest neighbors to consider

num_features : int, default=1260

Number of top features to select (5% of 21,542 genes)

Returns:

--------

top_features : array

Indices of selected features

scores : array

ReliefF importance scores for all features

"""

print(f"Running ReliefF with {n_neighbors} neighbors...")

\# Initialize ReliefF

relief = ReliefF(n_neighbors=n_neighbors,

n_features_to_select=num_features)

\# Fit the model

relief.fit(X, y)

\# Get feature importance scores

scores = relief.feature_importances_

\# Get indices of top features

top_features = np.lexsort((np.arange(len(scores)), -scores))[:num_features]

print(f"Selected {len(top_features)} features")

print(f"Feature importance score range: {scores.min():.4f} to {scores.max():.4f}")

return top_features, scores

\# =============================================================================

\# 4. SUPPLEMENTARY METHODS (mRMR and F-Test) - OPTIONAL

\# =============================================================================

def feature_selection_mrmr(X, y, num_features=1260):

"""Feature selection using Mutual Information (mRMR)."""

mi = mutual_info_classif(X, y)

top_features = np.argsort(mi)[-num_features:]

return top_features, mi

def feature_selection_ftest(X, y, num_features=1260):

"""Feature selection using F-test (ANOVA)."""

f_values, _ = f_classif(X, y)

top_features = np.argsort(f_values)[-num_features:]

return top_features, f_values

\# =============================================================================

\# 5. APPLY FEATURE SELECTION

\# =============================================================================

\# CORRECTED: Use all samples (no train/test split due to small sample size, n=12)

\# ReliefF was applied to the entire dataset of all 21,542 genes

total_genes = X.shape[1] # Should be 21,542

\# num_features = int(0.05 * total_genes) # 5% of total genes = 1,260

num_features = 1260 # Corrected: Assign as an integer, not a tuple

print(f"Total genes: {total_genes}")

print(f"Selecting top {num_features} features (5% of total)")

\# Convert X to float type for ReliefF to handle division operations correctly

X = X.astype(np.float64)

\# Apply ReliefF to all samples

features_relieff, scores_relieff = feature_selection_relieff(

X, y, n_neighbors=10, num_features=num_features

)

\# Optional: Apply supplementary methods

\# features_mrmr, scores_mrmr = feature_selection_mrmr(X, y, num_features)

\# features_ftest, scores_ftest = feature_selection_ftest(X, y, num_features)

\# =============================================================================

\# 6. SAVE RESULTS (CORRECTED)

\# =============================================================================

\# Define output directory

output_dir = "/content/drive/My Drive/Revised_ReliefF_Algorithm_Feature_Selection_for_Gastric_Cancer_Transcriptomic_Data/results"

os.makedirs(output_dir, exist_ok=True)

\# 6.1. Save feature importances

feature_names = [f"Gene_{i}" for i in features_relieff]

importance_df = pd.DataFrame({

"Feature": feature_names,

"ReliefF_Weight": scores_relieff[features_relieff]

})

\# Sort by importance (highest first)

importance_df = importance_df.sort_values("ReliefF_Weight", ascending=False)

importance_df.to_csv(

os.path.join(output_dir, "ReliefF_feature_importances.csv"),

index=False

)

print(f"Feature importances saved to {output_dir}ReliefF_feature_importances.csv")

\# 6.2. Save the reduced dataset (top 1,260 genes)

X_reduced = X[:, features_relieff]

reduced_data = pd.DataFrame(

X_reduced,

columns=[f"Gene_{i}" for i in features_relieff]

)

reduced_data['Target'] = y

reduced_data.to_csv(

os.path.join(output_dir, "reduced_dataset_top1260.csv"),

index=False

)

print(f"Reduced dataset saved to {output_dir}reduced_dataset_top1260.csv")

\# 6.3. Save summary statistics

summary_stats = {

"Total_genes": [total_genes],

"Selected_features": [num_features],

"Percentage_selected": [5.0],

"ReliefF_weight_range_min": [scores_relieff.min()],

"ReliefF_weight_range_max": [scores_relieff.max()],

}

summary_df = pd.DataFrame(summary_stats)

summary_df.to_csv(

os.path.join(output_dir, "feature_selection_summary.csv"),

index=False

)

print(f"Summary statistics saved to {output_dir}feature_selection_summary.csv")

\# =============================================================================

\# 7. VALIDATION: Leave-One-Out Cross-Validation (LOOCV)

\# =============================================================================

print("\n" + "="*60)

print("VALIDATION: Leave-One-Out Cross-Validation (LOOCV)")

print("="*60)

\# Use a simple classifier for validation (e.g., Random Forest)

classifier = RandomForestClassifier(

n_estimators=100,

random_state=SEED,

n_jobs=1

)

\# Perform LOOCV on the reduced dataset

loo = LeaveOneOut()

cv_scores = cross_val_score(classifier, X_reduced, y, cv=loo)

print(f"LOOCV Accuracy: {cv_scores.mean():.4f} (+/- {cv_scores.std():.4f})")

print(f"Number of samples: {len(cv_scores)}")

\# =============================================================================

\# 8. CLASSIFICATION REPORT (Optional)

\# =============================================================================

\# Train classifier on all data (for demonstration)

classifier.fit(X_reduced, y)

\# Since we don't have a separate test set, we report training performance

\# Note: This is for demonstration only; LOOCV is the primary validation method

y_pred = classifier.predict(X_reduced)

print("\nClassification Report (Training Data):")

print(classification_report(y, y_pred, target_names=['Normal', 'Tumor']))

print("\nFeature selection completed successfully.")
