# Supplementary material for: Uncovering the molecular landscape of young-onset diffuse gastric cancer: A relieff-based feature selection analysis on RNA-Seq data
Source: PLoS One. 2026 Aug 6;21(8):e0354961. doi: 10.1371/journal.pone.0354961 (PMC13446702; doi:10.1371/journal.pone.0354961)
Supplement: Supplementary file 3 [file pone.0354961.s009.docx]

**Supplementary Table S2.** The top 50 most informative upregulated and downregulated genes identified by the RReliefF feature selection algorithm.

| **DEGs** | **Gene name** |
| --- | --- |
| Upregulated | *PRND, WNT2, FAP, FNDC1, COL8A1, ITGBL1, P4HA3, SERPINE1, CRB1, STRA6, ADAMTS12, CTHRC1, SDS, SIRPD, PRRX1, DKK2, DCLK3, THSD7B, COL4A1, NOX4, MGP, CHRNA6, BGN, DPYSL5, THY1, DCSTAMP, THBS2, CPZ, MFAP2, GABRQ, SPHK1, FOXS1, PMCH, PGF, GABRD, KRT36, L1TD1, CCIN, CAMK1G, NKAIN4, CCR8, TREM2, CACNA1E, LKAAEAR1, GPR176, ELOVL2, OR2L13, MPP4, ANGPT2, COL6A3, DGKI, ASPN* |
| Downregulated | *PDIA2, GAS2L2, CD164L2, LGI3, MDFIC2, NEUROD1, VSTM2A, CPSF4L, SCGN, CHGA, GPHA2, C7orf78, LEMD1, GPR148, SLC7A3, SST, TUBB8B, SVOP, GAD2, UMOD, LTF, CLPS, GABRG2, DNAI1, KRT24, CHGB, PSAPL1, STXBP5L, CPLX2, NOL4, ARHGDIG, KIF1A, ASCL1, ROPN1B, GKN2, SLC38A3, AVPR1B, SCG3, C6, ADH1C, NSG2, MT1A, NPY, SLC5A5, FOXN1, RIPPLY3, ABCC8, LDHD, NUPR2* |
