# Supplementary material for: Uncovering the molecular landscape of young-onset diffuse gastric cancer: A relieff-based feature selection analysis on RNA-Seq data
Source: PLoS One. 2026 Aug 6;21(8):e0354961. doi: 10.1371/journal.pone.0354961 (PMC13446702; doi:10.1371/journal.pone.0354961)
Supplement: Supplementary file 6 [file pone.0354961.s019.docx]

**Manuscript ID: PONE-D-25-58282**

**Uncovering the Molecular Landscape of Young-Onset Diffuse Gastric Cancer: A ReliefF-Based Feature Selection Analysis on RNA-Seq Data**

Dear Dr. Zhanzhan Li (Academic Editor) and Reviewers,

We thank you for the opportunity to revise our manuscript. We are grateful to the reviewers for their thoughtful, rigorous, and constructive comments, which have significantly improved the clarity, transparency, and scientific rigor of our work. Below, we provide a point-by-point response to all comments from the Academic Editor and each reviewer. All changes in the manuscript have been marked using track changes.

=============================================================================

**Academic Editor** **– Journal Requirements**

=============================================================================

**Comment 1: Please ensure that your manuscript meets PLOS ONE's style requirements.**

**Response**:

We have reformatted the entire manuscript according to the PLOS ONE templates. This includes proper file naming, reference formatting, Tables and figure placement. All figure legends have been moved to the end of the manuscript, immediately after the References section.

=============================================================================

**Comment 2: Code sharing – author-generated code must be made available without restrictions.**

**Response**:

The complete Python code for ReliefF analysis has been deposited in a public GitHub repository: <https://github.com/sjsajadi/UncoveringtheMolecularLandscapeofYoungOnsetDiffuseGastric.git>.

The code is also included as S2 Table in the supplementary files.

=============================================================================

**Comment 3: Please ensure that you refer to Figure 14 in your text.**

**Response**:

Thank you for pointing this out. The name of this figure is changed to Fig 10 according to the reviewers´ comment. We have corrected the citation in the manuscript: **Results section (Page 25; Lines 447-454; Subsection 3.10. Prediction of transcription factors of Hub genes:**

Transcriptional regulatory network analysis using the TRRUST database identified several key transcription factors (TFs) as central regulators of the DGC hub gene landscape (Fig 10, Table 5). The TFs NFKB1 and RELA emerged as the most statistically significant master regulators (FDR = 1.57e-09 and 1.43e-07, respectively), demonstrating a complex regulatory profile by predominantly activating hub genes including *CCL5*, *CXCR4*, *FOXP3*, *IL18*, and *MMP9*. Notably, both TFs also exhibited a dual role by repressing *MMP9*. Further analysis defined a cohesive regulatory module wherein hub genes like *MMP9* and *CXCR4* are co-regulated by a suite of TFs. This intricate network pinpoints NF-κB signaling and MMP9 regulation as critical, multifaceted nodes in DGC pathogenesis.

=============================================================================

**Comment 4: Please upload a new copy of Figures 6D, 8A-8G, 9A-9G, 10, 11, 13, and 14 as the detail is not clear.**

**Response**:

All figures have been regenerated at 600 dpi resolution with improved contrast, labeling, and legibility.

=============================================================================

**Comment 5: Please review and evaluate any recommended citations from reviewers.**

**Response**:

We have carefully reviewed all citations suggested by the reviewers. Those that were directly relevant to our methods or findings have been incorporated into the reference list. Those that were not directly relevant have been noted but not cited, as permitted by the editor's instructions.

=============================================================================

**Reviewer 1**

=============================================================================

We sincerely thank Reviewer 1 for the positive and constructive feedback. Your recognition of the novelty, comprehensive analysis, and clinical translational potential of our work is greatly appreciated.

=============================================================================

**Comment 1.1- Clarification on Sample Size and Pilot Nature:** The authors correctly and transparently frame this as a pilot study. To further preempt potential reviewer concerns, it would be beneficial to briefly expand the discussion on the inherent limitations of the small sample size (n=6 per group). A sentence or two could explicitly discuss how future studies with larger, independent cohorts are essential to validate these findings and to move from hypothesis-generation to a definitive predictive model.

**Response**:

We agree completely. In the revised manuscript, we have added a dedicated paragraph in the **Discussion section** **(Page 34; lines 609-617)**:

While our pilot study successfully demonstrates the feasibility of integrating machine learning with transcriptomic data for DGC analysis, the small sample size (n=6 per group) imposes important limitations, and our findings should be considered hypothesis-generating rather than definitive. Therefore, we strongly encourage future studies with larger, independent cohorts to validate the candidate genes and pathways identified here and to transition from hypothesis generation to definitive predictive modeling. In addition, all findings presented here require independent validation through in vitro (*e.g*., qPCR, Western blot, functional assays) and in vivo studies before any clinical translation. We also acknowledge the absence of experimental validation. While our bioinformatic predictions are supported by multiple lines of evidence (literature, network analysis, and public database validation), definitive proof of causality requires targeted in vitro experiments such as gene knockdown/overexpression studies, migration/invasion assays, and immunohistochemical confirmation at the protein level.

=============================================================================

**Comment 1.2 (Statistical interpretation):** The handling of the MMP9 result is appropriate (acknowledging the high fold-change despite non-significant p-value). To enhance clarity, consider adding a brief statement in the results or discussion explicitly attributing this discrepancy to the pilot-scale sample size and its impact on statistical power, which the ReliefF algorithm is designed to mitigate. This reinforces the methodological choice.

**Response**:

Thank you for this excellent suggestion. We have added a clear sentence in the **Discussion section (Page 30-31; Lines: 533-545).** The new text reads:

MMP9, a zinc-dependent matrix metalloproteinase, plays a critical role in GC progression and metastasis by degrading extracellular matrix components, notably type IV collagen in basement membranes (103, 104). Its expression is influenced by factors such as *H. pylori* infection and specific promoter polymorphisms like the -1562C/T variant, which elevates its mRNA and protein levels (105). Functionally, inhibiting MMP9 has been shown to suppress distant metastasis in GC through an MMP- 9/PI3K/AKT/Snail-dependent pathway, underscoring its therapeutic relevance (106). In our analysis, *MMP9* exhibited the most pronounced overexpression (fold change = 16.6) among all hub genes, yet this did not reach statistical significance (p = 0.34) (Table 1). This discrepancy between a large effect size and a non-significant p-value is a well-documented phenomenon, often attributed to factors such as limited sample size, technical variation, or tumor heterogeneity, rather than a lack of biological relevance. For example, the substantial fold change, consistent with extensive research linking *MMP9* overexpression to advanced GC stage and poor prognosis, solidifies its position as a biologically pivotal node in DGC progression, warranting further investigation in larger cohorts (107, 108). The ReliefF algorithm, which captures multivariate relationships, prioritized *MMP9* as a top feature despite the non-significant univariate p-value, highlighting the complementary value of machine learning approaches in small-sample transcriptomic studies.

============================================================================

**Comment 1.3 (Terminology consistency):** There is a minor inconsistency in the abstract and highlights regarding the number of "central players." The abstract mentions "three central players—CXCR4, MMP9, and TNFSF13B," while the first highlight states "a core hub gene network (CXCR4, MMP9, TNFSF13B)." However, the introduction lists seven hub genes. This is not incorrect but could be slightly confusing. Consider using a consistent term such as "**three prioritized core hub genes**" or "three central hub genes" to distinguish them from the **initial seven identified** through network analysis.

**Response**:

This has been corrected. Throughout the abstract and highlights, we now consistently use the phrase " three prioritized core hub genes" (CXCR4, MMP9, TNFSF13B) to clearly distinguish them from the initial set of seven hub genes identified through network analysis:

**Abstract (Results**;**Page 2; line 34-36):** Our ML‑driven approach identified seven hub genes central to DGC pathogenesis including *CCL5*, *CXCR4*, *MMP9*, *FOXP3*, *IL18*, *TNFSF11*, and *TNFSF13B*. Among these, three prioritized core hub genes (*CXCR4*, *MMP9*, and *TNFSF13B*) were selected based on statistically significant overexpression and complementary functional roles.

**Highlights (Page 3; line 52-53; point 1):** changed from "a core hub gene network" to "a prioritized core hub gene network (CXCR4, MMP9, TNFSF13B)":

1. Machine learning identifies **a prioritized core hub gene** network (*CXCR4*, *MMP9*, *TNFSF13B*) driving diffuse gastric cancer (DGC) pathogenesis, revealing key drivers of metastasis, immune evasion, and extracellular matrix remodeling.

**Results (Page 16; Line 337; Subsection 3.3. Analysis of protein-protein interaction network and Hub gene modules):** added clarifying sentence: Subsequent analysis of the PPI network via the cytoHubba plugin in Cytoscape identified **the initial seven hub genes** including, CCL5 (chemokine ligand 5), CXCR4 (C-X-C chemokine receptor type 4 or RANTES), .....

**Results (Page 19; Line 380; Subsection 3.5. Validation and Prognostic Value of Hub Genes):** We further assessed the prognostic significance of the **initial seven hub genes** by generating overall survival (OS) curves in GEPIA, ....

**Results (Page 25; Line 449-451; Subsection 3.10. Prediction of transcription factors of Hub genes):** The TFs NFKB1 and RELA emerged as the most statistically significant master regulators (FDR = 1.57e-09 and 1.43e-07, respectively), demonstrating a complex regulatory profile by predominantly activating **hub genes** including CCL5, CXCR4, FOXP3, IL18, and MMP9.

**Discussion (Page 29; Line 510):** Network analysis identified **initial seven hub genes** exhibiting high connectivity, CCL5, CXCR4, MMP9, FOXP3, IL18, TNFSF11, and TNFSF13B, ......

**Discussion (Page 33; Line 589):** The final selection of CXCR4, MMP9, and TNFSF13B as **prioritized core hub genes** was driven by their statistical significance, elevated expression profiles, and complementary functional roles in DGC pathogenesis.

=============================================================================

**Reviewer 2:**

**Comment 2.1 (Lack of experimental validation):** The authors analyzed publicly available RNA-seq data from diffuse gastric cancer using machine learning-based feature selection to prioritize hub genes. They identified CXCR4, MMP9, and TNFSF13B as a core hub gene network with the potential to drive diffuse gastric cancer. They also found that the VEGF inhibitor Bevacizumab indirectly targets CXCR4 and MMP9. Additionally, they proposed TNFSF13B as a novel and highly significant immunomodulatory hub gene. All analyses are based on public sequencing datasets, and no experiments were conducted to validate their hypotheses. While bioinformatics data-mining approaches are useful and acceptable, the hypotheses generated should be further tested and verified through in vitro and in vivo studies. Without such validation, the analysis lacks meaningful support.

**Response**:

We appreciate Reviewer 2's candid assessment. Bioinformatics analyses of public data are a legitimate and widely accepted first step in cancer research, especially for rare diseases like young-onset DGC where sample collection is extremely difficult. We believe our study provides a valuable resource for guiding future experimental work. Our manuscript is explicitly framed as a pilot bioinformatics study with the primary aim of hypothesis generation, not definitive validation. We have been transparent about this

from the **Abstract (Subsection** **Conclusion**; **Page 2; Line 43)**: "This pilot study establishes a robust integrative framework..."),

through the **Introduction** (**Page 6; Line 119-121):** Ultimately, we seek to generate robust hypotheses about oncogenic pathways and candidate driver genes, thereby laying the groundwork for future validation studies with larger cohorts."),

to the **Discussion** (**Page 34; Line 609-613):** While our pilot study successfully demonstrates the feasibility of integrating machine learning with transcriptomic data for DGC analysis, the small sample size (n=6 per group) imposes important limitations, and our findings should be considered hypothesis-generating rather than definitive. Therefore, we strongly encourage future studies with larger, independent cohorts to validate the candidate genes and pathways identified here and to transition from hypothesis generation to definitive predictive modeling.

Also, in the **Conclusion** **section** **(Page 34; Lines 623-626):** Although definitive confirmation in a larger, independent cohort is an essential next step, this work provides a powerful methodological foundation and valuable insights. We believe this pilot study will be a valuable resource for guiding future research, and its findings are poised for significant improvement with increased sample sizes, paving the way for more precise diagnostic and therapeutic strategies in this aggressive cancer.).

Additionally, we have substantially strengthened the manuscript based on your comment.

In the **Discussion** **(Page 34; Lines 613-617),** we have added this paragraph: In addition, all findings presented here require independent validation through in vitro (*e.g*., qPCR, Western blot, functional assays) and in vivo studies before any clinical translation. We also acknowledge the absence of experimental validation. While our bioinformatic predictions are supported by multiple lines of evidence (literature, network analysis, and public database validation), definitive proof of causality requires targeted in vitro experiments such as gene knockdown/overexpression studies, migration/invasion assays, and immunohistochemical confirmation at the protein level.

=============================================================================

**Comment 2.2 (Poor organization and figure legends):** The manuscript is poorly organized. The figure legends should be placed at the end of the manuscript.

**Response**:

We have completely reorganized the manuscript according to PLOS ONE standards. All figure legends have been moved to the end of the manuscript, immediately following the References section.

=============================================================================

**Reviewer 3:**

The core idea of this article is to identify several key transcription factors and central genes that play important roles in the pathogenesis of diffuse gastric cancer (DGC) by analyzing the transcription factor central gene interactions in DGC.

CXCR4, MMP9, and TNFSF13B were identified as central genes in the study, and NFKB1 and RELA were found to be the most important transcription factors. These genes and transcription factors promote tumor proliferation and invasion by activating purine signaling pathways and enhancing the immunosuppressive tumor microenvironment.

In addition, the study also explored the role of these genes in tumor immune escape and growth, and proposed the VEGF inhibitor Bevacizumab as a potential strategy for treating DGC. The research results lay the foundation for future validation studies with larger sample sizes, which will help develop more accurate diagnostic and treatment strategies.

**Response**:

We greatly appreciate your positive evaluation of our work. Your summary accurately captures the core findings and significance of our study: the identification of *CXCR4*, *MMP9*, and *TNFSF13B* as central genes, the role of NFKB1 and RELA as master transcription factors, and the potential of Bevacizumab as a therapeutic strategy. We thank you for your time and thoughtful review. No specific revisions were requested, and we have incorporated your encouraging feedback as we finalized the manuscript.

=============================================================================

**Reviewer 4:**

Authors address an important gap in understanding the molecular disruptions that occur in diffuse gastric cancer and aim to define driver genes and their function to propose potential therapeutic interventions. Generally, the multi-layered approach of leveraging various types of annotation on protein-protein interactions, tumor micro-environment, survival, miRNA regulation and other methods is viable. Given the small number of sample sizes, authors aimed to provide depth into the potential functional impact of genes relevant to DGC by leveraging public knowledge sources. However, the clarity of the methods and approach used is insufficient to reproduce the results, and some results/conclusions are contradictory and overstated.

**Response**:

We thank Reviewer 4 for the detailed and rigorous critique. Your comments have significantly improved the clarity, transparency, and scientific rigor of our work. Below, we address each major and minor comment systematically.

=============================================================================

**Comment 4.1 (Sample information):** Further information about the materials and methods should be included such as: Clear description of all samples that are used in the study, including age, sex, and biospecimen type (e.g., blood, tissue, etc.) at the minimum.

**Response**:

We have expanded **Table 1** **(now refer to S1 Table)** to include all available demographic and clinical information from the GEO datasets. The revised table now includes the following columns: GSE ID, GSM ID, SRA ID, Sample Type (Tumor/Normal), Histology, Biospecimen type, Age, Sex, and Library Layout.

All samples were derived from gastric tissue (either normal mucosa or diffuse-type tumor tissue).

For the GSE113255 dataset, age and sex were provided for cancer samples only in the original GEO records; we have explicitly stated "Not available " for normal samples to maintain transparency.

**S1 Table**. Cohort description of the exploratory RNA-seq analysis in young-onset diffuse gastric cancer. This table documents the most important data for the 12-sample pilot cohort of 6 DGC tumors and 6 normal tissues from datasets GSE113255 and GSE122401. N/A: Not aviable in GEO database.

| No. | GSE ID | GSM ID | SRA ID | Sample Type | Histology | Biospecimen type | Age | Sex | Library  Layout |
| --- | --- | --- | --- | --- | --- | --- | --- | --- | --- |
| 1 | GSE113255 | GSM3101070 | SRR7014300 | Normal | Normal mucosae | Tissue | N/A | N/A | Paired‐end |
| 2 | GSE113255 | GSM3101072 | SRR7014302 | Normal | Normal mucosae | Tissue | N/A | N/A | Paired‐end |
| 3 | GSE113255 | GSM3101068 | SRR7014298 | Normal | Normal mucosae | Tissue | N/A | N/A | Paired‐end |
| 4 | GSE122401 | GSM3501782 | SRR8281317 | Normal | Normal mucosae | Tissue | 33 | Male | Paired‐end |
| 5 | GSE122401 | GSM3501783 | SRR8281318 | Normal | Normal mucosae | Tissue | 29 | Male | Paired‐end |
| 6 | GSE122401 | GSM3501784 | SRR8281319 | Normal | Normal mucosae | Tissue | 30 | Female | Paired‐end |
| 7 | GSE113255 | GSM3101104 | SRR7014334 | Tumor | Diffuse Type | Tissue | 34 | Male | Paired‐end |
| 8 | GSE113255 | GSM3101117 | SRR7014347 | Tumor | Diffuse Type | Tissue | 25 | Female | Paired‐end |
| 9 | GSE113255 | GSM3101139 | SRR7014369 | Tumor | Diffuse Type | Tissue | 35 | Male | Paired‐end |
| 10 | GSE122401 | GSM3501862 | SRR8281397 | Tumor | Diffuse Type | Tissue | 33 | Male | Paired‐end |
| 11 | GSE122401 | GSM3501863 | SRR8281398 | Tumor | Diffuse Type | Tissue | 29 | Male | Paired‐end |
| 12 | GSE122401 | GSM3501864 | SRR8281399 | Tumor | Diffuse Type | Tissue | 30 | Female | Paired‐end |

GSE113255: https://www.ncbi.nlm.nih.gov/geo/query/acc.cgi?acc=GSE113255

GSE122401: https://www.ncbi.nlm.nih.gov/geo/query/acc.cgi?acc=GSE122401

=============================================================================

**Comment 4.2 (Batch effects between datasets):** Authors are combining samples from two different studies. Have they evaluated the feasibility of this? Are there possible batch effects that should be considered?Are control samples showing similar profiles? Similarly, authors combined cohort and GTEx normal tissue samples for survival analyses. How were these samples combined? How many samples are are utilized? Are the normal tissue appropriate for comparison against the STAD cohort (e.g., demographic matching)?

**Response**:

We thank the reviewer for this important methodological question. To assess the feasibility of combining datasets GSE113255 and GSE122401, we performed Principal Component Analysis (PCA) on the normalized expression matrix of all 12 samples (6 normal and 6 tumor). As shown in Supplementary Figure S1 (S1 Fig):

**S1 Fig. Principal Component Analysis (PCA) of the combined RNA-seq datasets GSE113255 and GSE122401.** PCA was performed on the normalized expression matrix of all 12 samples (6 normal and 6 tumor gastric tissues) from two independent GEO datasets. Samples are colored by tissue type (Normal: blue; Tumor: red) and shaped by dataset origin (GSE113255: circles; GSE122401: triangles).

**Samples cluster primarily by tissue type:** PC1 (27.86% of variance) clearly separates normal samples from tumor samples, confirming that the primary source of variation is biological (tissue type).

**No separation by dataset origin:** PC2 (21.46% of variance) shows no clear clustering by dataset (GSE113255 vs. GSE122401). Normal samples from both datasets form a single cluster, confirming that control samples show highly similar expression profiles regardless of dataset origin.

**Expected tumor heterogeneity:** Two tumor samples (SRR7014369 and SRR8281398) cluster near normal samples, reflecting the known molecular heterogeneity of diffuse gastric cancer (DGC).

**Batch effect removal:** Based on these PCA results, we conclude that no significant batch effects are present and that combining GSE113255 and GSE122401 is feasible. Batch correction was not applied, as it could remove true biological variation and introduce bias in this small sample size (n=6 per group).

We have added the following sentence to the **Materials and Methods section** **(Page 7; Lines 134-138;** **Subsection 2.1. Data Acquisition and Preprocessing**):

To assess and mitigate potential batch effects when combining datasets GSE113255 and GSE122401, we performed Principal Component Analysis (PCA) on the normalized expression matrix of all 12 samples (6 normal and 6 tumor) using the prcomp function in R version 4.3.2 (within the RStudio environment 2023.12.1) with scaling and centering (30). Visualization was performed using the ggplot2 package to confirm the absence of significant batch-related clustering prior to downstream differential expression analysis.

Ref 30. Harvey DT, Hanson BA. A Comparison of Functions for PCA. dim (A). 2024;1(4):5.

We have added the following sentence to the **Results section** **(Page 15; Lines 294-301;** **subsection** **3.1**):

PCA of the combined dataset confirmed samples clustered primarily by tissue type (tumor vs. normal) rather than by dataset origin, with no apparent separation between the two GEO series, supporting the feasibility of combining these datasets (S1 Fig). Normal samples from both datasets (GSE113255 and GSE122401) formed a distinct cluster, indicating similar expression profiles regardless of dataset origin. Interestingly, two tumor samples (SRR7014369 and SRR8281398) clustered near normal samples, reflecting the known molecular heterogeneity of DGC and the intrinsic variability of tumor samples. These findings confirm the absence of significant batch effects and support the feasibility of combining GSE113255 and GSE122401 for downstream analyses. Therefore, no batch correction was applied, as doing so could inadvertently remove true biological variation and introduce bias, particularly given the limited sample size (n=6 per group).

=============================================================================

**Comment 4.3 (References and versions for tools):** References and versions are missing in the methods for tools that are used (e.g., CLC Genomic Workbench, FastQC, STRING, EPIC method, miRNA databases, etc).

**Response**:

This has been corrected. In the revised Methods section, we have added the version number and references for every software tool, database, and algorithm used. Specifically:

**- FastQC v0.11.9 (Babraham Institute): Subsection 2.2.1. Quality Control Assessment of Raw Reads; Page 8; Line 150:**

Brown, J., Pirrung, M., & McCue, L. A. (2017). FQC Dashboard: integrates FastQC results into a web-based, interactive, and extensible FASTQ quality control tool. Bioinformatics, 33(19), 3137-3139.

**-** **CLC Genomics Workbench** **v20.0**: **Subsection 2.2.2. Read Trimming and Filtering; Page 8; Line 154:**

Liu, C.-H., & Di, Y. P. (2020). Analysis of RNA sequencing data using CLC genomics workbench. In *Molecular toxicology protocols* (pp. 61-113). Springer.

Sequencing, H. (2011). CLC genomics workbench. *Workbench*.

**- ENSEMBL database: Subsection 2.2.3. Read Alignment to Reference Genome; Page 8; Line 163**

Dyer, S. C., Austine-Orimoloye, O., Azov, A. G., Barba, M., Barnes, I., Barrera-Enriquez, V. P., Becker, A., Bennett, R., Beracochea, M., & Berry, A. (2025). Ensembl 2025. Nucleic acids research, 53(D1), D948-D957.

**- Python 3.10:** **Subsection 2.3.2. Candidate Gene Selection Using Machine Learning Algorithm; Page 10; Line 198:**

Van Rossum, G., & Drake Jr, F. L. (2014). The python language reference. Python Software Foundation: Wilmington, DE, USA.

**-** **Scikit-learn (v1.2.2):** **Subsection 2.3.2. Candidate Gene Selection Using Machine Learning Algorithm; Page 10; Line 198:**

Hao, J., & Ho, T. K. (2019). Machine learning made easy: a review of scikit-learn package in python programming language. Journal of educational and behavioral statistics, 44(3), 348-361.

**-** **Google Colab: Subsection 2.3.2. Candidate Gene Selection Using Machine Learning Algorithm; Page 10; Line 198:**

Bisong, E. (2019). Google colaboratory. In Building machine learning and deep learning models on google cloud platform: a comprehensive guide for beginners (pp. 59-64). Springer.

**- DAVID v6.8 : Subsection 2.4. Functional Annotation and Pathway Enrichment Analysis of Candidate Genes; Page 10; Line 207:**

Huang DW, Sherman BT, Tan Q, Kir J, Liu D, Bryant D, et al. DAVID Bioinformatics Resources: expanded annotation database and novel algorithms to better extract biology from large gene lists. Nucleic acids research. 2007;35(suppl_2):W169-W75.

**-SRplot online: Subsection 2.4. Functional Annotation and Pathway Enrichment Analysis of Candidate Genes; Page 10; Line 207:**

Tang D, Chen M, Huang X, Zhang G, Zeng L, Zhang G, et al. SRplot: A free online platform for data visualization and graphing. PloS one. 2023;18(11):e0294236.

**- STRING database v11.5: Subsection 2.5. Construction of Protein-Protein Interaction Network and Hub Gene Identification; Page 11; Line 214:**

Szklarczyk, D., Nastou, K., Koutrouli, M., Kirsch, R., Mehryary, F., Hachilif, R., Hu, D., Peluso, M. E., Huang, Q., & Fang, T. (2025). The STRING database in 2025: protein networks with directionality of regulation. Nucleic acids research, 53(D1), D730-D737.

**- Cytoscape (v3.10.3): Subsection 2.5. Construction of Protein-Protein Interaction Network and Hub Gene Identification; Page 11; Line 214:**

Shannon P, Markiel A, Ozier O, Baliga NS, Wang JT, Ramage D, et al. Cytoscape: a software environment for integrated models of biomolecular interaction networks. Genome research. 2003;13(11):2498-504.

**- GEPIA2: Subsection 2.6. Validation of Hub Genes' Expression and Prognostic Significance; Page 12; Line 235:**

Tang Z, Kang B, Li C, Chen T, Zhang Z. GEPIA2: an enhanced web server for large-scale expression profiling and interactive analysis. Nucleic acids research. 2019;47(W1):W556-W60.

**-** **GeneMANIA v3.6.0: Subsection 2.7. Co-Expression and Transcriptional Regulatory Network Analysis; Page 12; Line 246:**

Franz M, Rodriguez H, Lopes C, Zuberi K, Montojo J, Bader GD, et al. GeneMANIA update 2018. Nucleic acids research. 2018;46(W1):W60-W4.

**- TIMER3.0: Subsection 2.8. Evaluation of Immune Cell Infiltration Patterns Associated with Hub Genes; Page 13; Line 263:**

Cui H, Zhao G, Lu Y, Zuo S, Duan D, Luo X, et al. TIMER3: an enhanced resource for tumor immune analysis. Nucleic Acids Research. 2025:gkaf388.

**- EPIC method as implemented in TIMER3.0: Subsection 2.8. Evaluation of Immune Cell Infiltration Patterns Associated with Hub Genes; Page 13; Line 263:**

Racle, J., & Gfeller, D. (2020). EPIC: a tool to estimate the proportions of different cell types from bulk gene expression data. In Bioinformatics for cancer immunotherapy: methods and protocols (pp. 233-248). Springer.

**- TargetScan v8.0:** **Subsection 2.9. Post-transcriptional Regulatory Network of Hub Genes; Page 13; Line 272:**

Agarwal, V., Bell, G. W., Nam, J.-W., & Bartel, D. P. (2015). Predicting effective microRNA target sites in mammalian mRNAs. elife, 4, e05005.

McGeary SE, Lin KS, Shi CY, Pham TM, Bisaria N, Kelley GM, et al. The biochemical basis of microRNA targeting efficacy. Science. 2019;366(6472):eaav1741.

**- miRDB v6.0: Subsection 2.9. Post-transcriptional Regulatory Network of Hub Genes; Page 13; Line 272:**

Chen Y, Wang X. miRDB: an online database for prediction of functional microRNA targets. Nucleic acids research. 2020;48(D1):D127-D31.

**- miRTarBase v9.0: Subsection 2.9. Post-transcriptional Regulatory Network of Hub Genes; Page 13; Line 272:**

Huang H-Y, Lin Y-C-D, Li J, Huang K-Y, Shrestha S, Hong H-C, et al. miRTarBase 2020: updates to the experimentally validated microRNA–target interaction database. Nucleic acids research. 2020;48(D1):D148-D54.

**- DGIdb v5.0: Subsection 2.10. Therapeutic Repurposing Opportunities Targeting Hub Genes; Page 14; Line 282:**

Cannon M, Stevenson J, Stahl K, Basu R, Coffman A, Kiwala S, et al. DGIdb 5.0: rebuilding the drug–gene interaction database for precision medicine and drug discovery platforms. Nucleic acids research. 2024;52(D1):D1227-D35.

**- TRRUST v2: Subsection 2.11. Transcriptional Regulatory Network Governing Hub Gene Expression; Page 14; Line 290:**

Han H, Cho J-W, Lee S, Yun A, Kim H, Bae D, et al. TRRUST v2: an expanded reference database of human and mouse transcriptional regulatory interactions. Nucleic acids research. 2018;46(D1):D380-D6.

All references have also been added to the reference list.

=============================================================================

**Comment 4.4 (Top 5% expressed genes – clarification):** Focusing the analyses on only the top 5% most expressed genes is quite restrictive (lines 177-178). Are these top 5% based on mean expression only? Or differential expression, with or without statistical significance? From text later in the manuscript, the assumption is that these 1,260 are derived from ReliefF but then the statement on lines 177-178 is incorrect and confusing. Please clarify.

**Response**:

We thank the reviewer for this important clarification. We acknowledge that the original text was ambiguous and contained an error regarding the filtering step. The correct analytical workflow is as follows.

1. ReliefF was applied to all 21,542 genes to assign a weight to each gene based on its ability to discriminate between tumor and normal samples.

2. Genes were ranked by their ReliefF weights, and we retained the top 5% of genes (n = 1,260) with the highest weights. This is a standard ReliefF-based feature selection approach, as previously validated in transcriptomic studies.

3. The 1,260 genes represent the output of the ReliefF algorithm, not an expression-based pre-filter.

We have revised the **Materials and Methods section** **(Subsection 2.3.2. Candidate Gene Selection Using Machine Learning Algorithm; Page 10; Lines 192-199)** to accurately describe this process:

To identify the most informative genes associated with DGC, we applied the ReliefF algorithm to the entire normalized expression matrix containing all 21,542 genes. ReliefF is a multivariate feature selection method that assigns a weight to each feature based on its ability to discriminate between instances of different classes (tumor vs. normal) by evaluating the nearest neighbors in the multidimensional space. This approach enables the identification of genes involved in complex biological interactions that might be overlooked by univariate methods. The analysis was implemented in Python 3.10 using scikit-learn (v1.2.2) within Google Colab (https://colab.research.google.com/), configured with k=10 nearest neighbors and 100 iterations. Genes were then ranked by their ReliefF weights in descending order (36-38). To reduce computational complexity for downstream analyses while maintaining biological relevance, we retained the top 5% of genes (n = 1,260) for subsequent network and functional enrichment analyses.

We also corrected the **Results section** **(Subsection 3.1. Identification of differentially expressed genes (DEGs) using feature selection methods; Page 15; Lines 302-311):**

The ReliefF algorithm was applied to the full expression matrix of all 21,542 genes. Based on the ReliefF weights, genes were ranked by their discriminatory power between tumor and normal samples. From this ranking, we selected the top 5% of genes (n = 1,260) with the highest ReliefF weights as candidate features for downstream analysis. To determine the direction of expression changes, we compared the mean expression values of each gene between tumor and normal samples. Genes with higher mean expression in tumor samples were classified as upregulated, while those with lower mean expression in tumor samples were classified as downregulated. Among the 1,260 selected genes, 918 were upregulated and 342 were downregulated in cancerous tissues compared to normal tissues. S3 Table summarizes the top 50 upregulated and downregulated genes among those selected by the ReliefF algorithm, based on their mean expression differences between tumor and normal samples. Additionally, the complete results of the differential expression analysis performed using CLC Genomics Workbench v20.0 and the ReliefF algorithm are provided in S4 and S5 Tables, respectively.

=============================================================================

**Comment 4.5 (Stability of ReliefF weights with 80:20 split):** Feature selection with ReliefF, mRMR, and F Test (Supplementary materials): The code indicates that authors split the data into training and testing with an 80:20 split. Did authors repeat that training/testing and evaluate the stability of the ReliefF weights, as well as the mRMR, and F test feature selection? While the authors are limited by the sample size, the stability (and thus ability to replicate) of the results could be demonstrated a bit further.

**Response**:

You have identified an error in our code documentation. In the actual analysis, due to the very small sample size (n=12), we did NOT perform a training-test split. The split shown in the code was a remnant from a previous analysis on a different dataset and has been removed. We have corrected the code in the Supplementary file (S2 Table).

=============================================================================

**Comment 4.6 (Number 1262 in code):** The code indicates that “num_features=1262”. How was this determined? (Also for clarity, it may be best to remove that “num_features=1382” from the function definitions as those are overwritten with 1262 during the function calls.).

**Response**:

This was a typographical error. The correct number is 1,260 (which is 5% of 21,542 genes, rounded to the nearest integer). The code in the Supplementary (S2 Table) file has been corrected to num_features=1260.

=============================================================================

**Comment 4.7 (diff() function in code):** What is the meaning of the function diff() in the pseudocode of Supp File 1?

**Response**:

The diff() function was inadvertently left in the code from a previous version. It is not used in the main analysis of this manuscript and has been removed from the final code. We thank you for catching this error. We have corrected the code in the Supplementary file (S2 Table).

=============================================================================

**Comment 4.8 (PPI network – all interaction types?):** For the PPI network, were all interaction types (e.g., predicted and experimentally validated) utilized? What was the date of access?

**Response**:

We thank the reviewer for this important methodological question. To ensure transparency and reproducibility, we provide the following clarifications:

1. Interaction types included: Yes, we included all interaction types available in the STRING database (v11.5) with a confidence score ≥ 0.7. Specifically, we included: (a) experimentally validated interactions, (b) predicted interactions, (c) co-expression (based on correlated expression patterns across multiple datasets), (d) automated text-mining (from co-occurrence in PubMed abstracts), and (e) database-curated interactions (from manually curated pathway databases such as KEGG and Reactome). This comprehensive approach maximizes the capture of both well-established and predicted functional relationships among the candidate genes.

2. Date of access: The STRING database (v11.5) was accessed on December 15, 2024.

We have revised the **Materials and Methods section** **(2.5. Construction of Protein-Protein Interaction Network and Hub Gene Identification; Page 11; Line 209-217)** to clearly state the date of access and the inclusion of all interaction types.

To explore the functional relationships and identify the most central genes among the candidate set, a protein-protein interaction (PPI) network was constructed for the candidate genes identified by the ReliefF algorithm using the StringApp plugin (v2.2.0) in Cytoscape (v3.10.3). All interaction types (predicted and experimentally validated) were retrieved from the STRING database (v11.5) with a minimum confidence score threshold of 0.7. From the resulting network, hub genes were identified using the CytoHubba plugin (v0.1) by applying the Maximal Clique Centrality (MCC) algorithm. The top 7 nodes ranked by MCC score were selected as hub genes for subsequent validation and prognostic analysis (41, 42). While PPI networks derived from transcriptomic data provide a useful framework for understanding potential functional interactions, it is important to note that mRNA levels do not always reflect protein activity. However, these predictions assume a direct relationship between gene expression and protein function.

=============================================================================

**Comment 4.9 (TPM vs. RPKM):** Authors use TPM in some analyses (e.g., tumor immune microenvironment) but it seems RPKM with others. Is this correct? If so, an explanation of why different data are used as input would be helpful.

**Response**:

We acknowledge that different normalization units were used for different analyses, and we provide the following explanation. We sincerely thank the reviewer for this insightful comment. We apologize for the lack of clarity in our original manuscript. Upon re-evaluation, we confirm that our analysis of the tumor immune microenvironment was performed exclusively using the 'Gene' module of the TIMER3 webserver, and we did not perform any independent correlation analysis on our own cohort. In this module, users simply provide gene names, and the TIMER3 server automatically retrieves pre-calculated expression data from its integrated TCGA database. Importantly, these data are already normalized as log2(TPM+1) by the server, and the correlations (specifically, purity-adjusted partial Spearman's correlations) are computed internally. Therefore, for this specific analysis, no conversion of our own RNA-seq data (RPKM) was required or performed, as the analysis was entirely based on the server's internal TCGA-STAD data. To avoid any ambiguity, we have revised Subsection 2.8 of the Materials and Methods (2.8. Evaluation of Immune Cell Infiltration Patterns Associated with Hub Genes) to clearly state that all correlation analyses were performed internally by the TIMER3 server and that no user-side data normalization was necessary. The revised text now accurately reflects our methodology and confirms that the correlation results presented in Fig 7 and Table 2 are the direct output from the TIMER3 webserver. We are confident that this clarification resolves the reviewer's concern. We appreciate the reviewer's diligence in pointing out this potential ambiguity, which has significantly improved the clarity and accuracy of our manuscript.

**Materials and Methods (Subsection 2.8. Evaluation of Immune Cell Infiltration Patterns Associated with Hub Genes; Page 12-13; Line 247-263):**

To investigate the potential impact of the identified hub genes on the tumor immune microenvironment (TME) and their association with immune cell infiltration, the relationship between the 7 hub genes and the TME was evaluated using the TCGA- STAD cohort as a validation dataset via the TIMER3.0 web server (https://compbio.cn/timer3/). TIMER3.0 is a comprehensive web-based platform that systematically analyzes immune cell infiltration across multiple cancer types using TCGA and other public datasets. Although the STAD cohort comprises multiple histological subtypes, it provides a robust platform for the initial validation of TME interactions in gastric cancer.

To comprehensively characterize the TME, the following analyses were performed: First, the abundance of five immune cell types (B cells, CD8+ T cells, CD4+ T cells, macrophages, and natural killer (NK) cells) was estimated via the EPIC (Estimating the Proportion of Immune and Cancer cells) method, which uses deconvolution of bulk transcriptomic data to infer immune cell fractions. Second, to gain a global view of the TME composition, we extracted three key scores for each sample: the Immune Score_ESTIMATE (reflecting infiltrating immune cells), Stroma Score_ESTIMATE (reflecting stromal presence), and Estimate Score_ESTIMATE (inversely correlating with tumor purity). Additionally, the MSI Score_TIDE was retrieved as a predictor of microsatellite instability and potential immune escape. To assess the associations between hub gene expression and TME characteristics, Pearson's correlation analysis was performed between each hub gene's expression level (log2(TPM+1)) and each of the immune cell abundances and TME scores obtained from TIMER3.0. Statistical significance was defined as p < 0.05, with both correlation coefficients (R) and corresponding p-values reported for complete interpretation of the relationships (45, 46).

=============================================================================

**Comment 4.10 (Line 245 – 1,260 genes selected by ReliefF?):** Line 245 states that 1,260 genes were selected by ReliefF, yet the methods state that the 1,260 are genes with the highest average expression across all samples (line 178). Did the 1,260 genes result from differential analyses?

**Response**:

The upregulation and downregulation status of the selected genes was determined separately by comparing mean expression values between tumor and normal samples (as explained in response to comment 4.4). This was done only to characterize the biological direction of expression changes among the already-selected genes, not to select them.

The Methods section has been corrected to accurately describe the ReliefF-based selection process.

The Results section now explicitly states that the 1,260 genes were selected by ReliefF and clarifies that up/down classification is a separate descriptive analysis.

**Material and Methods** **section** **(Subsection 2.3.2. Candidate Gene Selection Using Machine Learning Algorithm; Page 10; Line 192-199)**

To identify the most informative genes associated with DGC, we applied the ReliefF algorithm to the entire normalized expression matrix containing all 21,542 genes. ReliefF is a multivariate feature selection method that assigns a weight to each feature based on its ability to discriminate between instances of different classes (tumor vs. normal) by evaluating the nearest neighbors in the multidimensional space. This approach enables the identification of genes involved in complex biological interactions that might be overlooked by univariate methods. The analysis was implemented in Python 3.10 using scikit-learn (v1.2.2) within Google Colab (https://colab.research.google.com/), configured with k=10 nearest neighbors and 100 iterations. Genes were then ranked by their ReliefF weights in descending order (36-38). To reduce computational complexity for downstream analyses while maintaining biological relevance, we retained the top 5% of genes (n = 1,260) for subsequent network and functional enrichment analyses.

**Results section (Subsection 3.1. Identification of differentially expressed genes (DEGs) using feature selection methods; Page 15; Line 302-311)**

... The ReliefF algorithm was applied to the full expression matrix of all 21,542 genes. Based on the ReliefF weights, genes were ranked by their discriminatory power between tumor and normal samples. From this ranking, we selected the top 5% of genes (n = 1,260) with the highest ReliefF weights as candidate features for downstream analysis. To determine the direction of expression changes, we compared the mean expression values of each gene between tumor and normal samples. Genes with higher mean expression in tumor samples were classified as upregulated, while those with lower mean expression in tumor samples were classified as downregulated. Among the 1,260 selected genes, 918 were upregulated and 342 were downregulated in cancerous tissues compared to normal tissues. S3 Table summarizes the top 50 upregulated and downregulated genes among those selected by the ReliefF algorithm, based on their mean expression differences between tumor and normal samples. Additionally, the complete results of the differential expression analysis performed using CLC Genomics Workbench v20.0 and the ReliefF algorithm are provided in S4 and S5 Tables, respectively.

=============================================================================

**Comment 4.11 (Transcriptome vs. proteome limitation):** Authors are measuring the transcriptome yet are using significant genes to draw PPI networks. While this can be useful, the limitation is the assumption that all genes are translated into functional proteins. This limitation should be stated.

**Response**:

The reviewer raises a valid limitation that is often overlooked in transcriptome-based studies. We completely agree that constructing PPI networks from transcriptomic data inherently assumes that changes in mRNA levels correlate with changes in protein abundance and activity, which is not always the case due to post-transcriptional and post-translational regulatory mechanisms.

We add this limitiation in **Material and Methods** **section (Subsection 2.5. Construction of Protein-Protein Interaction Network and Hub Gene Identification; Page 11; Line 214-217)**

.... While PPI networks derived from transcriptomic data provide a useful framework for understanding potential functional interactions, it is important to note that mRNA levels do not always reflect protein activity. However, these predictions assume a direct relationship between gene expression and protein function.

=============================================================================

**Comment 4.12 (Cross-validation between ReliefF and CLC):** Line 299: authors state that results from ReliefF were “cross-validated against the differential expression results obtained from the CLC Genomics Workbench 20.0 pipeline.” What does this mean? Section 2.2 in the methods that describe the CLC Genomics Workbench does not describe any differential analysis. (Also, “CLC Genomics” and “CLC Genomic” are used, please be consistent).

**Response**:

We address both points below.

**1. Clarification of "Cross-Validation:** We acknowledge that the term "cross-validated" must be changed to a comparative assessment because our analysis did not involve a formal statistical cross-validation (e.g., k-fold or leave-one-out). Instead, we performed a comparative assessment to evaluate the concordance between the genes selected by ReliefF and those identified as differentially expressed by the CLC Genomics Workbench pipeline.

We have revised **Material and Methods** **section** **(Subsection 2.5. Construction of Protein-Protein Interaction Network and Hub Gene Identification; Page 11; Line 209-222)** to provide a full description of the differential expression analysis performed using CLC Genomics Workbench 20.0:

To explore the functional relationships and identify the most central genes among the candidate set, a protein-protein interaction (PPI) network was constructed for the candidate genes identified by the ReliefF algorithm using the StringApp plugin (v2.2.0) in Cytoscape (v3.10.3). All interaction types (predicted and experimentally validated) were retrieved from the STRING database (v11.5) with a minimum confidence score threshold of 0.7. From the resulting network, hub genes were identified using the CytoHubba plugin (v0.1) by applying the Maximal Clique Centrality (MCC) algorithm. The top 7 nodes ranked by MCC score were selected as hub genes for subsequent validation and prognostic analysis (41, 42). While PPI networks derived from transcriptomic data provide a useful framework for understanding potential functional interactions, it is important to note that mRNA levels do not always reflect protein activity. However, these predictions assume a direct relationship between gene expression and protein function. To confirm the consistency of expression patterns, the candidate hub genes selected via CytoHubba were further compared with the differential expression results obtained from the CLC Genomics Workbench v20.0 RNA-Seq analysis pipeline. Genes were considered significantly differentially expressed if they met the criteria of an absolute log2 fold change > 1 and a p-value < 0.05 (35). This comparison aimed to confirm the directionality (upregulated or downregulated) and statistical significance of the selected hub genes prior to downstream validation.

As well as, we revised the **Results** **section** **(Subsection 3.3. Analysis of protein-protein interaction network and Hub gene modules; Page 17; Line 342-354)**

Following the identification of candidate hub genes through a ReliefF-based feature selection analysis, the results were compared with the differential expression results obtained from the CLC Genomics Workbench v20.0 pipeline (Table 1). As shown in Table 1, all seven hub genes showed consistent directional changes (upregulated or downregulated) between the ReliefF and CLC differential expression analyses. In the CLC analysis, *MMP9* demonstrated the most substantial fold change (16.58), followed by *TNFSF11* (4.73) and *CXCR4* (3.12). However, only two genes reached statistical significance in the conventional differential expression test including *CXCR4* (FDR = 0.01) and *TNFSF13B* (FDR < 0.000001). The remaining genes, including *CCL5* (FDR = 1.00), *FOXP3* (FDR = 0.14), *IL18* (FDR = 0.82), *MMP9* (FDR = 0.34), and *TNFSF11* (FDR = 0.06), did not achieve significance in the univariate analysis. This discrepancy is expected given the fundamental differences between univariate and multivariate approaches, *i.e.,* univariate tests assess each gene independently and are limited by statistical power, while multivariate methods like ReliefF capture complex interactions and coordinated expression patterns that may not be detectable in single-gene analyses. The complementary nature of these approaches, univariate tests identifying genes with large individual effects and ReliefF identifying genes with coordinated multivariate signals, provides a more comprehensive understanding of the molecular landscape of DGC.

**Table 1.** Comparative analysis of hub gene expression from the ReliefF-based feature selection algorithm and the CLC Genomics Workbench v20.0 differential expression pipeline.

| **Feature ID** | **ReliefF Weights** | **Mean Expression (Normal Mean RPKM)** | **Mean Expression (Tumor Mean RPKM)** | **Direction (ReliefF-based)** | **Fold Change (Baggerley's test, CLC)** | **FDR p-value (Baggerley's test, CLC)** |
| --- | --- | --- | --- | --- | --- | --- |
| **CCL5** | 0.070 | 977.42 | 1025.07 | Up | 1.09 | 1.00 |
| **CXCR4** | -0.017 | 1116.93 | 3318.95 | Up | 3.12 | **0.01** |
| **FOXP3** | -0.015 | 43.25 | 126.00 | Up | 3.04 | 0.14 |
| **IL18** | 0.027 | 395.80 | 313.68 | Down | -1.22 | 0.82 |
| **MMP9** | 0.033 | 146.40 | 2343.65 | Up | 16.58 | 0.34 |
| **TNFSF11** | -0.005 | 10.93 | 50.60 | Up | 4.73 | 0.06 |
| **TNFSF13B** | 0.014 | 173.60 | 413.10 | Up | 2.32 | **< 0.000001** |

The ReliefF weights for the seven hub genes are also presented in Table 1. Among the hub genes, *CCL5* exhibited the highest ReliefF weight (0.0699), indicating its strong multivariate discriminatory power between tumor and normal samples. *MMP9* (0.0334) and *IL18* (0.0274) also showed positive weights, confirming their contribution to class separation. *TNFSF13B* (0.0141) displayed a weak positive weight, while *TNFSF11* (-0.0045), *FOXP3* (-0.0152), and *CXCR4* (-0.0165) showed negative or near-zero weights, suggesting limited contribution to multivariate class separation despite their significance in univariate differential expression or PPI network centrality. Notably, *CXCR4* demonstrated a high fold change (3.12) but a negative ReliefF weight (-0.0165), highlighting the fundamental difference between univariate and multivariate feature selection approaches. While *CXCR4* shows differential expression between tumor and normal tissues in univariate analysis, its multivariate discriminatory power is limited, possibly due to heterogeneous expression patterns across samples or its interaction with other genes in the network. These findings underscore the complementary value of ReliefF in identifying genes with true multivariate discriminatory power, and together with PPI network analysis, provide a robust framework for prioritizing biologically relevant hub genes in DGC.

**2. Terminology Consistency:** We have corrected all instances of "CLC Genomic" to "CLC Genomics Workbench 20.0" throughout the manuscript, including the Methods, Results, and Supplementary Materials, to ensure consistent and accurate terminology.

=============================================================================

**Comment 4.13 (Table 3 – Baggerley's test and ReliefF weights):** In Table 3, the use of Baggerley’s test is not explained nor cited in the Methods. The assumption is that those metrics are outputs from CLC but this needs clarity. This table would also benefit from showing the actual ReliefF weights. As is, there is no direct comparative analysis as is indicated in the title of the table. The normalized expression values shown could be moved to a supplementary material to focus the attention on the feature selection itself. Also, an FDR of 0.00 should be described with more precision (e.g., scientific notation can be used if needed).

**Response**:

We thank the reviewer for this important methodological comment. We have carefully addressed each point raised and revised Table 3 **(now refer to Table 1)** accordingly. Below, we provide a detailed point-by-point response.

**1. Baggerley's test explanation and citation:** We acknowledge that the original manuscript did not adequately describe Baggerley's test. We have now added a clear explanation of this statistical test to the **Materials and Methods section (Subsection 2.2.4. Differential Expression Analysis and Statistical Assessment; Page 9; Line 173-180)**:

To identify statistically significant differentially expressed genes between tumor and normal samples, we employed the proportion‑based statistical analysis tool implemented within the CLC Genomics Workbench, which utilizes Baggerly's test. Baggerly's test is a rank‑based statistical method designed to compare expression proportions between two groups by evaluating the distribution of read counts across biological conditions. Unlike parametric tests that assume normal distribution and equal variances, Baggerly's test is robust to heteroscedasticity and does not require equal variance between groups, making it particularly suitable for small sample sizes and datasets with unbalanced group variances, characteristics inherent to our rare DGC dataset. Genes were considered significantly differentially expressed if they met the criteria of an absolute log₂ fold change > 1 and a p‑value < 0.05 (35).

We have added the following citation:

Baggerly, K. A., Deng, L., Morris, J. S., & Aldaz, C. M. (2003). Differential expression in SAGE: accounting for normal between-library variation. Bioinformatics, 19(12), 1477-1483.

**2. ReliefF weights were included in Table 3 (now refer to Table 1):** We appreciate the reviewer's suggestion to include ReliefF weights in Table 3 (now refer to Table 1). However, we respectfully note that the ReliefF weights are not directly comparable to the CLC differential expression metrics (fold change, p-value, FDR) for several reasons:

-Different scales: ReliefF weights range from -1 to +1, while fold changes are on a multiplicative scale.

-Different interpretations: ReliefF weights measure the ability of a feature to discriminate between classes based on nearest-neighbor distances in a multivariate space, whereas fold change measures the magnitude of expression difference between two groups. These are complementary but not directly comparable metrics.

-Different purposes: ReliefF is a feature selection algorithm that identifies genes with multivariate discriminatory power, while Baggerley's test is a univariate differential expression test. Including both metrics in the same table without proper context could confuse readers.

We have revised the table to provide a clear comparative analysis between the two methods by showing:

Fold change from Baggerley's test (CLC output)

FDR p-value from Baggerley's test (CLC output)

Mean normalized expression values for tumor and normal groups (calculated from the RPKM values)

Direction of change (upregulated/downregulated) by ReliefF-based results

This approach focuses on the biological relevance of the hub genes (their expression differences) rather than presenting non-comparable statistical metrics.

However, we have completely redesigned Table 3 (now refer to Table 1) to address the reviewer's concerns. The revised table and **Results section** now includes ReliefF Weights **(Subsection 3.3. Analysis of protein-protein interaction network and Hub gene modules; Page 17-18; Line 355-367)**:

**Table 1.** Comparative analysis of hub gene expression from the ReliefF-based feature selection algorithm and the CLC Genomics Workbench v20.0 differential expression pipeline.

| **Feature ID** | **ReliefF Weights** | **Mean Expression (Normal Mean RPKM)** | **Mean Expression (Tumor Mean RPKM)** | **Direction (****ReliefF-based)** | **Fold Change (Baggerley's test, CLC)** | **FDR p-value (Baggerley's test, CLC)** |
| --- | --- | --- | --- | --- | --- | --- |
| **CCL5** | 0.070 | 977.42 | 1025.07 | Up | 1.09 | 1.00 |
| **CXCR4** | -0.017 | 1116.93 | 3318.95 | Up | 3.12 | **0.01** |
| **FOXP3** | -0.015 | 43.25 | 126.00 | Up | 3.04 | 0.14 |
| **IL18** | 0.027 | 395.80 | 313.68 | Down | -1.22 | 0.82 |
| **MMP9** | 0.033 | 146.40 | 2343.65 | Up | 16.58 | 0.34 |
| **TNFSF11** | -0.005 | 10.93 | 50.60 | Up | 4.73 | 0.06 |
| **TNFSF13B** | 0.014 | 173.60 | 413.10 | Up | 2.32 | **< 0.000001** |

The ReliefF weights for the seven hub genes are also presented in Table 1. Among the hub genes, *CCL5* exhibited the highest ReliefF weight (0.0699), indicating its strong multivariate discriminatory power between tumor and normal samples. *MMP9* (0.0334) and *IL18* (0.0274) also showed positive weights, confirming their contribution to class separation. *TNFSF13B* (0.0141) displayed a weak positive weight, while *TNFSF11* (-0.0045), *FOXP3* (-0.0152), and *CXCR4* (-0.0165) showed negative or near-zero weights, suggesting limited contribution to multivariate class separation despite their significance in univariate differential expression or PPI network centrality. Notably, *CXCR4* demonstrated a high fold change (3.12) but a negative ReliefF weight (-0.0165), highlighting the fundamental difference between univariate and multivariate feature selection approaches. While *CXCR4* shows differential expression between tumor and normal tissues in univariate analysis, its multivariate discriminatory power is limited, possibly due to heterogeneous expression patterns across samples or its interaction with other genes in the network. These findings underscore the complementary value of ReliefF in identifying genes with true multivariate discriminatory power, and together with PPI network analysis, provide a robust framework for prioritizing biologically relevant hub genes in DGC.

**3. Move the normalized expression values to a supplementary material:** Table 1 (previously Table 3) has been revised and no longer contains the normalized expression values.

**4. FDR precision- scientific notation:** We have corrected the reporting of FDR values. For TNFSF13B, which previously showed "0.00", we now report it as "< 0.000001"  in Table 1 (previously Table 3). All FDR values are now reported with appropriate precision.

=============================================================================

**Comment 4.14 (Contradiction – "strong concordance" vs. only 2 significant genes):** Authors make the point that the ReliefF method is more powerful than traditional differential expression methods (e.g., t-tests) because it considers relationships between genes. While multivariate methods are indeed complementary to univariate ones, authors state in lines 299-301 that “This comparative analysis confirmed a strong concordance between the two methods, with the selected hub genes showing significant and consistent expression patterns.” This statement contradicts results in Table 3 that clearly show that only 2 of the 7 hub genes identified with ReliefF are statistically significant in differential analyses.

**Response**:

We have revised the manuscript to accurately reflect the relationship between the two methods. The revised text now clarifies that while all seven hub genes showed consistent directional changes, only CXCR4 and TNFSF13B reached statistical significance in the univariate differential expression test. This discrepancy is not a contradiction but rather a reflection of the complementary nature of univariate and multivariate approaches. Univariate tests are limited by statistical power (especially with n = 6 per group), while ReliefF captures multivariate interactions that may not be detectable in single-gene analyses. We have revised the **Results** **section** **(Subsection 3.3. Analysis of protein-protein interaction network and Hub gene modules; Page 17; Line 342-354)** to accurately describe this complementary relationship.

Following the identification of candidate hub genes through a ReliefF-based feature selection analysis, the results were compared with the differential expression results obtained from the CLC Genomics Workbench v20.0 pipeline (Table 1). As shown in Table 1, all seven hub genes showed consistent directional changes (upregulated or downregulated) between the ReliefF and CLC differential expression analyses. In the CLC analysis, *MMP9* demonstrated the most substantial fold change (16.58), followed by *TNFSF11* (4.73) and *CXCR4* (3.12). However, only two genes reached statistical significance in the conventional differential expression test including *CXCR4* (FDR = 0.01) and *TNFSF13B* (FDR < 0.000001). The remaining genes, including *CCL5* (FDR = 1.00), *FOXP3* (FDR = 0.14), *IL18* (FDR = 0.82), *MMP9* (FDR = 0.34), and *TNFSF11* (FDR = 0.06), did not achieve significance in the univariate analysis. This discrepancy is expected given the fundamental differences between univariate and multivariate approaches, *i.e.,* univariate tests assess each gene independently and are limited by statistical power, while multivariate methods like ReliefF capture complex interactions and coordinated expression patterns that may not be detectable in single-gene analyses. The complementary nature of these approaches, univariate tests identifying genes with large individual effects and ReliefF identifying genes with coordinated multivariate signals, provides a more comprehensive understanding of the molecular landscape of DGC.

**Table 1.** Comparative analysis of hub gene expression from the ReliefF-based feature selection algorithm and the CLC Genomics Workbench v20.0 differential expression pipeline.

| **Feature ID** | **ReliefF Weights** | **Mean Expression (Normal Mean RPKM)** | **Mean Expression (Tumor Mean RPKM)** | **Direction (ReliefF-based)** | **Fold Change (Baggerley's test, CLC)** | **FDR p-value (Baggerley's test, CLC)** |
| --- | --- | --- | --- | --- | --- | --- |
| **CCL5** | 0.070 | 977.42 | 1025.07 | Up | 1.09 | 1.00 |
| **CXCR4** | -0.017 | 1116.93 | 3318.95 | Up | 3.12 | **0.01** |
| **FOXP3** | -0.015 | 43.25 | 126.00 | Up | 3.04 | 0.14 |
| **IL18** | 0.027 | 395.80 | 313.68 | Down | -1.22 | 0.82 |
| **MMP9** | 0.033 | 146.40 | 2343.65 | Up | 16.58 | 0.34 |
| **TNFSF11** | -0.005 | 10.93 | 50.60 | Up | 4.73 | 0.06 |
| **TNFSF13B** | 0.014 | 173.60 | 413.10 | Up | 2.32 | **< 0.000001** |

The ReliefF weights for the seven hub genes are also presented in Table 1. Among the hub genes, *CCL5* exhibited the highest ReliefF weight (0.0699), indicating its strong multivariate discriminatory power between tumor and normal samples. *MMP9* (0.0334) and *IL18* (0.0274) also showed positive weights, confirming their contribution to class separation. *TNFSF13B* (0.0141) displayed a weak positive weight, while *TNFSF11* (-0.0045), *FOXP3* (-0.0152), and *CXCR4* (-0.0165) showed negative or near-zero weights, suggesting limited contribution to multivariate class separation despite their significance in univariate differential expression or PPI network centrality. Notably, *CXCR4* demonstrated a high fold change (3.12) but a negative ReliefF weight (-0.0165), highlighting the fundamental difference between univariate and multivariate feature selection approaches. While *CXCR4* shows differential expression between tumor and normal tissues in univariate analysis, its multivariate discriminatory power is limited, possibly due to heterogeneous expression patterns across samples or its interaction with other genes in the network. These findings underscore the complementary value of ReliefF in identifying genes with true multivariate discriminatory power, and together with PPI network analysis, provide a robust framework for prioritizing biologically relevant hub genes in DGC.

=============================================================================

**Comment 4.15 (CNET plots):** In Results, Line 271: a brief explanation of what CNET plots would help with understanding and readability.

**Response**:

We have replaced the CNET plots with Sankey‑dot plots, which provide a more comprehensive and visually intuitive representation of the relationships between genes and enriched functional terms. The Sankey diagram clearly illustrates the flow of gene‑to‑term associations, while the adjacent dot plot simultaneously displays the statistical significance (–log₁₀(p‑value)) and gene count (dot size) for each enriched term. This combined visualization enhances readability and allows readers to easily interpret both the connections and the enrichment significance in a single integrated figure. All figures related to GO and KEGG results are moved to Supplementary files.

**S2 Fig. Gene Ontology (GO) enrichment analysis of Biological Processes (BP) for up- and down-regulated genes in diffuse gastric cancer.** The integrated Sankey–dot plot illustrates the relationship between significantly enriched BP terms and their associated differentially expressed genes. (A) Enriched BP terms for up-regulated genes. (B) Enriched BP terms for down-regulated genes. The Sankey diagram (left) connects each GO term to the corresponding genes, with node width proportional to the number of genes annotated to each term. The dot plot (right) visualizes the statistical significance (–log₁₀(p-value)) and gene count (dot size) for each enriched term. Only the significant terms (p-value < 0.05) are displayed.

**S3 Fig. Gene Ontology (GO) enrichment analysis of Molecular Functions (MF) for up- and down-regulated genes in diffuse gastric cancer.** The combined Sankey–dot plot depicts the functional annotations of differentially expressed genes based on their molecular activities. (A) MF terms associated with up-regulated genes. (B) MF terms associated with down-regulated genes. The Sankey diagram (left) connects each GO term to the corresponding genes, with node width proportional to the number of genes annotated to each term. The dot plot (right) visualizes the statistical significance (–log₁₀(p-value)) and gene count (dot size) for each enriched term. Only the significant terms (p-value < 0.05) are displayed.

**S4 Fig. Gene Ontology (GO) enrichment analysis of Cellular Components (CC) for up- and down-regulated genes in diffuse gastric cancer.** The Sankey–dot plot provides a comprehensive overview of the subcellular localization of differentially expressed gene products. (A) CC terms enriched among up-regulated genes. (B) CC terms enriched among down-regulated genes. The Sankey diagram (left) connects each GO term to the corresponding genes, with node width proportional to the number of genes annotated to each term. The dot plot (right) visualizes the statistical significance (–log₁₀(p-value)) and gene count (dot size) for each enriched term. Only the significant terms (p-value < 0.05) are displayed.

=============================================================================

**Comment 4.16 (New methods introduced in Results – GEPIA, etc.):** Throughout the results, new methods and approaches are introduced that are not described in the methods. Examples include GEPIA and Baggerly’s test. Further, the language to describe the purpose of the analyses is inconsistent, making the readability of the work challenging, particularly for readers that may not be familiar with all the tools utilized. For example, the “drug-gene interaction of hub genes” section identifies potential repurposed drugs for DGC. Rather than labeling the section names by what the methods are doing, labeling section names by the impact of the methods (e.g., in this case drug repurposing), would provide much clarity throughout the manuscript.

**Response**:

We have revised the **Materials and Methods subsections**:

- **Subsection 2.1. Data Acquisition and Preprocessing; Page 7; Line 123-141**

Early‑onset gastric cancer (≤45 years) more frequently presents as diffuse-type lesions arising in histologically normal mucosa, suggesting a greater role for genetic alterations compared to conventional GC (60–80 years), which is more strongly influenced by environmental factors (29). Given that diffuse‑type gastric cancer consistently presents at a younger age than intestinal‑type gastric cancer, and to specifically investigate the molecular drivers of young‑onset DGC, we restricted our inclusion criteria to patients under 40 years of age. We performed a systematic search of the Gene Expression Omnibus (GEO) database (https://www.ncbi.nlm.nih.gov/) and selected all available samples that met the following criteria: first; all samples were under 40 years old, second; the cancerous tissues were diffuse type, and third; all samples were paired-end sequenced. Ultimately, 12 samples (6 normal and 6 tumor) from two datasets (GSE113255, GSE122401) were identified and selected for this pilot analysis (S1 Table). The mean age of patients with available age data in our cohort (n=9 out of 12 samples) was 30.9 years (range: 25–35 years; SD: ±3.4 years).

To assess and mitigate potential batch effects when combining datasets GSE113255 and GSE122401, we performed Principal Component Analysis (PCA) on the normalized expression matrix of all 12 samples (6 normal and 6 tumor) using the prcomp function in R version 4.3.2 (within the RStudio environment 2023.12.1) with scaling and centering (30). Visualization was performed using the ggplot2 package to confirm the absence of significant batch-related clustering prior to downstream differential expression analysis.

The overall analytical workflow for identifying and validating hub genes in DGC is summarized in Fig 1, which illustrates the integrated bioinformatics pipeline encompassing data acquisition, preprocessing, feature selection, network analysis, and comprehensive multi-omics validation employed throughout this study.

- **Subsection 2.2. Differential Expression Analysis of RNA-Seq Data; Page 7-9; Line 143-180**

**2.2.1. Quality Control Assessment of Raw Reads**

The RNA-Seq data analysis protocol commenced with rigorous quality control (QC) to eliminate low-quality reads and ensure the reliability of downstream analyses. Initial QC steps incorporated GC content analysis via binomial expectation modeling to assess sequence composition bias, k-mer frequency profiling to detect overrepresented sequences, and ambiguous base quantification to filter reads containing indeterminate nucleotides (N). Read accuracy was further evaluated through Phred score distribution analysis across read lengths, while Sequencing Reads QC tools (*e.g*., FastQC) provided a comprehensive assessment of read integrity. Adapter contamination was specifically addressed using exact k-mer alignment against a curated database of Illumina adapter sequences, ensuring the removal of artifactual sequences before downstream processing (31).

**2.2.2.**	**Read Trimming and Filtering**

Following QC assessment, the Trim Sequences Module in CLC Genomics Workbench v20.0 (Qiagen, Aarhus, Denmark; https://digitalinsights.qiagen.com/) was employed to process raw reads in FASTQ format (32, 33). The Trim Sequences tool uses a dynamic sliding window technique, calculating the average Phred quality score by traversing each read within a user-defined window (usually 5–15 bp). When the average quality fell below a threshold of Q20, bases from the ends of the reads were trimmed. Additionally, adapter sequences were eliminated by combining approximate matching based on a modified Levenshtein distance algorithm with exact k-mer matching, permitting a small number of mismatches. To maintain data integrity for subsequent analyses, reads shorter than 50 bp after trimming were excluded. Through this process, clean, high-quality data were generated for all downstream analyses.

**2.2.3.** **Read Alignment to Reference Genome**

The high-quality, trimmed reads were then aligned to the human reference genome GRCh38/hg38 in FASTA format, downloaded from the ENSEMBL database (34). Alignment was performed using the RNA-Seq Analysis pipeline in CLC Genomics Workbench. Read mapping leveraged a seed-and-extend alignment strategy, where initial seeds were identified using hash tables, and alignments were extended through dynamic programming based on a modified Smith-Waterman algorithm. Splice junctions were predicted using a Maximum Entropy Model trained on annotated exon-intron boundaries, thereby enhancing the accurate detection of spliced alignments. Mapping parameters were optimized for high specificity, allowing a maximum of two mismatches per read, requiring a minimum of 90% sequence identity, and filtering out multi-mapped reads based on alignment scores. Following successful alignment, transcript quantification was subsequently performed, and expression values were normalized using the Reads Per Kilobase of transcript per Million mapped reads (RPKM) method for subsequent differential expression analysis between tumor and normal samples (33).

**2.2.4. Differential Expression Analysis and Statistical Assessment**

To identify statistically significant differentially expressed genes between tumor and normal samples, we employed the proportion‑based statistical analysis tool implemented within the CLC Genomics Workbench, which utilizes Baggerly's test. Baggerly's test is a rank‑based statistical method designed to compare expression proportions between two groups by evaluating the distribution of read counts across biological conditions. Unlike parametric tests that assume normal distribution and equal variances, Baggerly's test is robust to heteroscedasticity and does not require equal variance between groups, making it particularly suitable for small sample sizes and datasets with unbalanced group variances, characteristics inherent to our rare DGC dataset. Genes were considered significantly differentially expressed if they met the criteria of an absolute log₂ fold change > 1 and a p‑value < 0.05 (35).

- **Subsection 2.3. Feature Selection and Identification of Candidate Genes via Machine Learning; Page 9-10; Line 182-207**

**2.3.1.** **Data Standardization and Preparation for Machine Learning**

A Python (v3.11.12) script was used for preprocessing of the transcriptomic data obtained from CLC Genomics Workbench v20.0 analysis (S2 Table). The data set was transposed that genes were the features (columns), and samples were the observations (rows). A label vector was created with a class of '0' for the six tumor (T) samples and '1' for the six normal (N) samples. Given the pilot and exploratory nature of the current study with the overall aim of finding a robust collection of candidate genes rather than training a general predictive model, all available samples (n=12) were utilized for feature selection. This approach maximizes detection power for differential expression patterns in this rare population. The feature values were subsequently normalized with the Z-score normalization (std_dev=1, mean=0) to avoid biasing the algorithms, particularly the distance-based ReliefF, by the inherent variation of the scales of gene expression.

**2.3.2.** **Candidate Gene Selection Using Machine Learning Algorithm**

To identify the most informative genes associated with DGC, we applied the ReliefF algorithm to the entire normalized expression matrix containing all 21,542 genes. ReliefF is a multivariate feature selection method that assigns a weight to each feature based on its ability to discriminate between instances of different classes (tumor vs. normal) by evaluating the nearest neighbors in the multidimensional space. This approach enables the identification of genes involved in complex biological interactions that might be overlooked by univariate methods. The analysis was implemented in Python 3.10 using scikit-learn (v1.2.2) within Google Colab (<https://colab.research.google.com/>), configured with k=10 nearest neighbors and 100 iterations. Genes were then ranked by their ReliefF weights in descending order (36-38). To reduce computational complexity for downstream analyses while maintaining biological relevance, we retained the top 5% of genes (n = 1,260) for subsequent network and functional enrichment analyses.

- **Subsection 2.4. Functional Annotation and Pathway Enrichment Analysis of Candidate Genes; Page 10; Line 200-208**

To elucidate the biological functions and signaling pathways associated with the candidate genes identified through ReliefF-based feature selection, functional enrichment analysis was performed on the candidate genes identified by the ReliefF algorithm using the Database for Annotation, Visualization, and Integrated Discovery (DAVID, v6.8; <https://david.ncifcrf.gov>) and SR plot online (https://bioinformatics.com.cn/en). The analysis identified significantly enriched Gene Ontology (GO) terms for biological processes (BP), cellular components (CC), and molecular functions (MF), as well as Kyoto Encyclopedia of Genes and Genomes (KEGG) pathways. Statistical significance was defined using a false discovery rate (FDR) threshold of < 0.05, calculated via the Benjamini-Hochberg method to correct for multiple testing (39, 40).

- **Subsection 2.5. Construction of Protein-Protein Interaction Network and Hub Gene Identification; Page 11; Line 209-222**

To explore the functional relationships and identify the most central genes among the candidate set, a protein-protein interaction (PPI) network was constructed for the candidate genes identified by the ReliefF algorithm using the StringApp plugin (v2.2.0) in Cytoscape (v3.10.3). All interaction types (predicted and experimentally validated) were retrieved from the STRING database (v11.5) with a minimum confidence score threshold of 0.7. From the resulting network, hub genes were identified using the CytoHubba plugin (v0.1) by applying the Maximal Clique Centrality (MCC) algorithm. The top 7 nodes ranked by MCC score were selected as hub genes for subsequent validation and prognostic analysis (41, 42). While PPI networks derived from transcriptomic data provide a useful framework for understanding potential functional interactions, it is important to note that mRNA levels do not always reflect protein activity. However, these predictions assume a direct relationship between gene expression and protein function. To confirm the consistency of expression patterns, the candidate hub genes selected via CytoHubba were further compared with the differential expression results obtained from the CLC Genomics Workbench v20.0 RNA-Seq analysis pipeline. Genes were considered significantly differentially expressed if they met the criteria of an absolute log_2_ fold change > 1 and a p-value < 0.05 (35). This comparison aimed to confirm the directionality (upregulated or downregulated) and statistical significance of the selected hub genes prior to downstream validation.

- **Subsection 2.6. Validation of Hub Genes' Expression and Prognostic Significance; Page 11-12; Line 224-235**

To visually confirm the differential expression of the hub genes within our own dataset, violin plots comparing their expression levels in DGC tumors versus normal tissues were generated using R version 4.3.2 within the RStudio environment (2023.12.1). For external validation using independent public datasets, the expression patterns and prognostic significance of the hub genes were assessed using the Gene Expression Profiling Interactive Analysis (GEPIA2, v2.0; http://gepia2.cancer-pku.cn) platform. GEPIA2 is an interactive web-based tool that integrates RNA-seq expression data from The Cancer Genome Atlas (TCGA) stomach adenocarcinoma (STAD) cohort and normal tissue samples from the Genotype-Tissue Expression (GTEx) project, providing a robust reference for validation. Survival analysis was performed through GEPIA2 by generating Kaplan-Meier curves, in which patients from the TCGA-STAD cohort were stratified into high and low expression groups based on the median expression level of each hub gene. Statistical significance of survival differences was determined using the log-rank test, with a significance threshold of p < 0.05. Differential expression of hub genes between TCGA tumor and GTEx normal tissues was visualized using box plots, with significance assessed by GEPIA2 via the Wilcoxon rank-sum test (43).

- **Subsection 2.7. Co-Expression and Transcriptional Regulatory Network Analysis; Page 12; Line 237-246**

To investigate the functional associations, potential co-expression patterns, and regulatory relationships of the identified hub genes, we constructed a gene-gene interaction network using GeneMANIA (v3.6.0; http://www.genemania.org). GeneMANIA is a web-based tool that predicts gene function and identifies functionally similar genes by integrating multiple genomic and proteomic interaction databases. The analysis was performed using all 7 hub genes identified through PPI network analysis, queried against the Homo sapiens genome-wide dataset with a limit of 20 resultant genes per query. The network integrated multiple interaction types, including co-expression, physical interactions, genetic interactions, and pathway co-membership derived from databases such as BioGRID and STRING. Interactions were weighted by confidence scores, with co-expressed genes requiring a Pearson correlation coefficient of > 0.4 for inclusion. Functional enrichment analysis of the genes within the resulting network was performed using GeneMANIA's built-in features to identify overrepresented biological pathways and functional categories associated with the hub genes and their interacting partners (44).

- **Subsection 2.8. Evaluation of Immune Cell Infiltration Patterns Associated with Hub Genes; Page 12-13; Line 248-263**

To investigate the potential impact of the identified hub genes on the tumor immune microenvironment (TME) and their association with immune cell infiltration, the relationship between the 7 hub genes and the TME was evaluated using the TCGA-STAD cohort as a validation dataset via the TIMER3.0 web server (<https://compbio.cn/timer3/>). TIMER3.0 is a comprehensive web-based platform that systematically analyzes immune cell infiltration across multiple cancer types using TCGA and other public datasets. Although the STAD cohort comprises multiple histological subtypes, it provides a robust platform for the initial validation of TME interactions in gastric cancer.

To comprehensively characterize the TME, the following analyses were performed: First, the abundance of five immune cell types (B cells, CD8+ T cells, CD4+ T cells, macrophages, and natural killer (NK) cells) was estimated via the EPIC (Estimating the Proportion of Immune and Cancer cells) method, which uses deconvolution of bulk transcriptomic data to infer immune cell fractions. Second, to gain a global view of the TME composition, we extracted three key scores for each sample: the Immune Score_ESTIMATE (reflecting infiltrating immune cells), Stroma Score_ESTIMATE (reflecting stromal presence), and Estimate Score_ESTIMATE (inversely correlating with tumor purity). Additionally, the MSI Score_TIDE was retrieved as a predictor of microsatellite instability and potential immune escape. To assess the associations between hub gene expression and TME characteristics, Pearson's correlation analysis was performed between each hub gene's expression level (log2(TPM+1)) and each of the immune cell abundances and TME scores obtained from TIMER3.0. Statistical significance was defined as p < 0.05, with both correlation coefficients (R) and corresponding p-values reported for complete interpretation of the relationships (45, 46).

- **Subsection 2.9. Post-transcriptional Regulatory Network of Hub Genes; Page 13; Line 264-272**

To identify potential microRNA (miRNA) regulators of the hub genes, a comprehensive in silico analysis was performed using three complementary databases: TargetScan (https://www.targetscan.org/vert_80/) for evolutionarily conserved miRNA-binding sites, miRDB (<https://mirdb.org/>) for machine-learning-based predictions, and miRTarBase (https://mirtarbase.cuhk.edu.cn/) for experimentally validated interactions. Database-specific quality thresholds were first applied, retaining only predictions with a probability of conserved targeting (Pct) > 0.9 in TargetScan and a target score > 90 in miRDB; all entries in miRTarBase were included to capitalize on available experimental evidence. To finalize a high-confidence set, only miRNA-gene interactions supported by at least two of the three databases were considered. Finally, the resulting high-confidence miRNA-hub gene regulatory network was visualized using SR plot online (47-50).

- **Subsection 2.10. Therapeutic Repurposing Opportunities Targeting Hub Genes; Page 14; Line 275-282**

To identify potential therapeutic agents that could be repurposed for targeting the identified hub genes, the 7 prioritized hub genes were systematically queried against the DGIdb (Drug-Gene Interaction Database, version 5.0; <https://dgidb.org/>). DGIdb is a comprehensive, publicly available resource that integrates drug-gene interaction data from over 30 curated sources, including DrugBank, PharmGKB, and ChEMBL. The search was configured to retrieve only interactions with known drug categories and evidence derived from curated sources. To prioritize clinically actionable candidates, resulting drug-gene pairs were filtered to highlight interactions specifically with existing FDA-approved compounds, with a particular focus on those with antagonistic or inhibitory mechanisms against the hub gene products, as these are most likely to offer therapeutic benefit in cancer treatment. This approach aimed to prioritize candidates for subsequent experimental validation (51).

- **Subsection 2.11. Transcriptional Regulatory Network Governing Hub Gene Expression; Page 14; Line 284-290**

To identify potential transcription factors (TFs) that regulate the expression of the identified hub genes at the transcriptional level, we employed TRRUST version 2 (Transcriptional Regulatory Relationships Unraveled by Sentence-based Text mining; <https://www.grnpedia.org/trrust/>). TRRUST is a curated database of transcriptional regulatory networks in human and mouse, constructed through systematic text mining of PubMed abstracts, providing experimentally validated and literature-curated TF-target interactions. The analysis was conducted using the database's built-in prediction tools to ascertain TFs that are significantly associated with the hub gene set. TF-hub gene interactions were considered significant if they met the threshold of an adjusted p-value < 0.05, and the direction of regulation (activation or repression) was recorded where available from the database (52).

=============================================================================

**Comment 4.17 (Drug repurposing vs. repositioning):** Also, please note that drug repurposing and drug repositioning are not synonymous and in this case, it seems that authors are attempting to perform drug repurposing. Please clarity.

**Response**:

We agree that drug repurposing and drug repositioning are not synonymous, and we have revised our manuscript accordingly to accurately reflect our approach. Drug repurposing refers to the identification of new therapeutic indications for existing, FDA-approved drugs that are already known to be safe and well-characterized in terms of pharmacokinetics and toxicity profiles. This strategy leverages existing clinical data to accelerate translation into clinical practice. In contrast, drug repositioning typically involves the re-evaluation of drug candidates that have previously failed in clinical trials for one indication, or the reformulation/re-dosing of existing drugs for the same or a related indication, rather than discovering a completely novel use. In our study, we systematically queried DGIdb to identify FDA-approved compounds that could potentially target the hub genes identified in DGC. Therefore, our approach is correctly categorized as drug repurposing, as we are seeking novel applications for already approved drugs. We have revised all instances of "drug repositioning" to "drug repurposing" throughout the manuscript, including in the Abstract and Results sections, to ensure terminological consistency and accuracy.

**Abstract; Page 2; Lines 32, 41**

Methods: ..... Their roles were further investigated through Gene Ontology, KEGG pathway analysis, tumor microenvironment immune infiltration, miRNA-regulatory network analysis, transcription factor prediction, and computational drug repurposing analyses.

Results: ..... Drug repurposing nominated several FDA-approved agents, including the VEGF-A inhibitor Bevacizumab, which indirectly suppresses CXCR4 and MMP9.

**Results section, Subsection 3.9. Drug-Gene Interaction of Hub genes; Page 23-24; Lines 434, 445**

Our computational drug repurposing analysis using the DGIdb database identified several FDA-approved drugs with high predicted affinity for key hub genes in DGC, revealing promising candidates for therapeutic repurposing (Fig 8, Table 4).

**Table 4**. Clinically approved drugs predicted to interact with hub genes in gastric cancer, as identified by a computational drug repurposing analysis. Drugs are listed with their regulatory status, primary indication, and predicted interaction score.

=============================================================================

**Comment 4.18 (CXCR4 as "biomarker" based only on K-M analysis):** Discussion, lines 560-561: authors state that “Kaplan-Meier overall survival (OS) analysis further established CXCR4 as a significant high-risk biomarker”. In and of itself, results from a univariate K-M analysis is not going to conclude that the analyzed gene is a significant biomarker. A biomarker requires validation that is not provided in this work.

**Response**:

We would like to clarify that our interest in CXCR4 was not based solely on survival analysis. In our study, CXCR4 demonstrated significant differential expression in our RNA-Seq analysis, with a Fold Change of 3.12 and an FDR-adjusted p-value of 0.01, indicating strong and statistically significant upregulation in DGC tumors compared to normal tissues. Therefore, CXCR4 showed consistent signals across two independent analytical approaches:

Analysis Method	Result

Differential Expression	Baggerly's test (CLC)	FC = 3.12, FDR = 0.01

Survival Association	Kaplan-Meier (GEPIA)	Log-rank p < 0.05

This dual evidence suggests that CXCR4 is not a randomly identified gene but rather a biologically relevant candidate that warrants further investigation. Our findings are consistent with the broader scientific consensus that CXCR4 overexpression is a reliable prognostic indicator in gastric cancer, particularly in the diffuse subtype (References 87-97).

Despite the strong evidence supporting CXCR4's prognostic role, we fully acknowledge the reviewer's critical distinction between a "prognostic indicator" and a "biomarker." We have therefore revised our manuscript accordingly:

**- Abstract; Page 2; Line 39-40:** changed from "high-risk prognostic biomarker" to "potential prognostic indicator":

High *CXCR4* expression was identified as a potential prognostic indicator associated with poorer overall survival in the TCGA‑STAD cohort (HR = 1.5, p = 0.0072).

**- Results (Subsection 3.5. Validation and Prognostic Value of Hub Genes); Page 19; Line 383-384:** changed from "identifying it as a prognostic biomarker" to "suggesting it as a “candidate prognostic marker requiring further validation":

Notably, elevated *CXCR4* expression (measured in transcripts per million, TPM) was also associated with a statistically significant 1.5-fold increase in mortality risk (*p* < 0.01), suggesting it as a candidate prognostic marker in GC requiring further validation.

**- Discussion; Page 33; Line 584-588:** changed from "established CXCR4 as a significant high-risk biomarker" to "suggested CXCR4 as a potential prognostic indicator":

Prognostic evaluation via Kaplan-Meier overall survival (OS) analysis further suggested CXCR4 as a potential prognostic indicator, where elevated expression was associated with a 1.5-fold increase in mortality risk (Hazard Ratio [HR] = 1.5, p < 0.01), underscoring its pivotal role in GC aggressiveness (Fig 5). However, multivariable analysis and validation in independent cohorts are necessary before it can be considered a true biomarker. In contrast, the expression levels of the other six hub genes did not demonstrate statistically significant correlations with patient survival (p > 0.05).

=============================================================================

**Comment 4.19 (Limitations beyond small sample size):** No limitations are provided except the small sample size.

**Response**:

In response to this valuable feedback, we have substantially expanded the Limitations in **Discussion section (Page 36; Lines 609-617)** to provide a more comprehensive and transparent acknowledgment of the methodological constraints and potential biases in our study.

While our pilot study successfully demonstrates the feasibility of integrating machine learning with transcriptomic data for DGC analysis, the small sample size (n=6 per group) imposes important limitations, and our findings should be considered hypothesis-generating rather than definitive. Therefore, we strongly encourage future studies with larger, independent cohorts to validate the candidate genes and pathways identified here and to transition from hypothesis generation to definitive predictive modeling. In addition, all findings presented here require independent validation through in vitro (*e.g*., qPCR, Western blot, functional assays) and in vivo studies before any clinical translation. We also acknowledge the absence of experimental validation. While our bioinformatic predictions are supported by multiple lines of evidence (literature, network analysis, and public database validation), definitive proof of causality requires targeted in vitro experiments such as gene knockdown/overexpression studies, migration/invasion assays, and immunohistochemical confirmation at the protein level.

=============================================================================

**Comment 4.20 (Abstract – imprecise language):** Abstract: this is a bit of a stylistic preference but expressions like “profound overexpression” are imprecise and could be rendered more impactful with more granular detail (e.g., two-fold, three-fold?). The additional granularity is also relevant for efforts that mine abstracts with AI. Another example is in line 114: “extreme rarity of this patient population”. This statement is inflammatory and does not account for ultra-rare diseases for which there is an N of 1. Please revise.

**Response**:

We completely agree that imprecise language such as "profound overexpression" and "extreme rarity" should be replaced with objective, measurable terminology to enhance both scientific rigor and machine readability. In response, we have systematically revised our manuscript to eliminate vague adjectives and replace them with precise quantitative data wherever possible:

**Abstract; Page 2; Line 34-42:**

**Results**: Our ML‑driven approach identified seven hub genes central to DGC pathogenesis including *CCL5*, *CXCR4*, *MMP9*, *FOXP3*, *IL18*, *TNFSF11*, and *TNFSF13B*. Among these, three prioritized core hub genes (*CXCR4*, *MMP9*, and *TNFSF13B*) were selected based on statistically significant overexpression and complementary functional roles. Specifically, *CXCR4* showed a Fold Change of 3.12 (Log₂FC = 1.64, FDR = 0.01), *MMP9* exhibited the highest magnitude of upregulation (Fold Change = 16.58, Log₂FC = 4.05), and *TNFSF13B* demonstrated the most statistically significant differential expression (Fold Change = 2.32, Log₂FC = 1.21, FDR < 0.000001). High *CXCR4* expression was identified as a potential prognostic indicator associated with poorer overall survival in the TCGA‑STAD cohort (HR = 1.5, p = 0.0072). We delineated a core regulatory circuitry where NF‑κB (NFKB1/RELA) masterfully regulates the hub gene network. Drug repurposing analysis nominated several FDA‑approved agents, including the VEGF‑A inhibitor Bevacizumab, which indirectly suppresses *CXCR4* and *MMP9*.

**Discussion; Page 30; Line 526-527:**

Alongside *CXCR4*, *TNFSF13B* (also known as BAFF or BLyS) emerged as a significantly upregulated hub gene in GC, demonstrating statistically significant overexpression compared to normal controls (p < 0.000001).

**Discussion; Page 33; Line 590-592:**

*CXCR4* and *TNFSF13B* were prioritized based on significant differential expression, mediating metastasis and a novel immunomodulatory axis, respectively, while *MMP9* was included due to its significant overexpression, underscoring its critical role in extracellular matrix remodeling.

Similarly, we have replaced the inflammatory phrase "extreme rarity of this patient population" with more measured language in the Introduction and Methods sections:

**Introduction; Page 6; Line 116-121:**

To the best of our knowledge, this study is the first to apply machine learning-based feature selection to RNA-seq data for the analysis of DGC in patients younger than 40 years. The limited number of cases of young-onset DGC has made it difficult to conduct large-scale studies and our study is designed as a pilot investigation. Our primary aim is not to provide a final predictive model, but to test the feasibility of this integrated transcriptomic and ML framework. Ultimately, we seek to generate robust hypotheses about oncogenic pathways and candidate driver genes, thereby laying the groundwork for future validation studies with larger cohorts.

**Material and Methods; Subsection 2.1. Data Acquisition and Preprocessing; Page 7; Line 124-133:**

We removed “Given the simultaneous rarity of young-onset GC and the DGC subtype,”:

Early‑onset gastric cancer (≤45 years) more frequently presents as diffuse-type lesions arising in histologically normal mucosa, suggesting a greater role for genetic alterations compared to conventional GC (60–80 years), which is more strongly influenced by environmental factors (29). Given that diffuse‑type gastric cancer consistently presents at a younger age than intestinal‑type gastric cancer, and to specifically investigate the molecular drivers of young‑onset DGC, we restricted our inclusion criteria to patients under 40 years of age. We performed a systematic search of the Gene Expression Omnibus (GEO) database (https://www.ncbi.nlm.nih.gov/) and selected all available samples that met the following criteria: first; all samples were under 40 years old, second; the cancerous tissues were diffuse type, and third; all samples were paired-end sequenced. Ultimately, 12 samples (6 normal and 6 tumor) from two datasets (GSE113255, GSE122401) were identified and selected for this pilot analysis (S1 Table). The mean age of patients with available age data in our cohort (n=9 out of 12 samples) was 30.9 years (range: 25–35 years; SD: ±3.4 years).

=============================================================================

**Comment 4.21 (Age range – "older individuals"):** It would also be appropriate to provide the incidence of DGC by age, since the focus of this manuscript is on patients that are young.You noted that the range 16-82 years contradicts the word "older."

P.4, line 76: authors write “presents in older individuals (16-82 years)”. Could authors provide the mean and SD of age in DGC globally and in the samples that are being analyzed? The range is so broad that the numbers do not fit the word “older”.

**Response**:

We completely agree that describing an age range of 16–82 years as "older individuals" is both inaccurate and misleading. We revised it as **(Introduction; Page 5; Line 79-81)**: In contrast, sporadic DGC may arise from acquired somatic mutations, without a significant family history, and lacks a hereditary component, thus shifting its management toward risk factor modification and symptomatic detection rather than prophylactic surgery.

We acknowledge that there is some variation in the reported mean ages for general diffuse‑type gastric cancer across different populations:

1. Perrot‑Applanat, M., Vacher, S., Pimpie, C., Chemlali, W., Derieux, S., Pocard, M., & Bieche, I. (2019). Differential gene expression in growth factors, epithelial mesenchymal transition and chemotaxis in the diffuse type compared with the intestinal type of gastric cancer. Oncology letters, 18(1), 674-686.:

“The median age of patients with diffuse GC was significantly lower 57 (27–71) years as compared with 75 (59–82) years for intestinal-subtype”

2. Zheng, H., Takahashi, H., Murai, Y., Cui, Z., Nomoto, K., Miwa, S., ... & Takano, Y. (2007). Pathobiological characteristics of intestinal and diffuse-type gastric carcinoma in Japan: an immunostaining study on the tissue microarray. Journal of clinical pathology, 60(3), 273-277.: The patients with gastric carcinoma included 222 men and 94 women (mean age 66.2 years; range 38–88 years).

Table 2:

| **Clinicopathological features** | **Intestinal‐type carcinoma** | **Diffuse‐type carcinoma** |
| --- | --- | --- |
| Histological types according to WHO classification | Papillary and tubular adenocarcinoma; part of moderately differentiated adenocarcinoma | Poorly differentiated or undifferentiated adenocarcinoma, SRC |
| Age (years), mean (SD)) | 67.8 (10.9), n = 163* | 64.5 (10.5) n = 153 |

3. Barbosa-Matos, R., Córdova, L., Schrader, K., & Oliveira, C. (2024). Diffuse Gastric and Lobular Breast Cancer Syndrome. GeneReviews®[Internet].:

“The average age of onset of DGC in the largest cohorts of individuals with CDH1-related DGLBCS ranges from age 42 to 49 years [Xicola et al 2019, Garcia-Pelaez et al 2023, Ryan et al 2024], and DGC onset may occur as young as age 14 years [Gullo et al 2018, Xicola et al 2019, São José et al 2023].”

4. <https://www.pathologyoutlines.com/topic/stomachdiffuse.html>: Stomach Carcinoma Diffuse type: More common in younger patients: mean age 48 years

5. Thrift, A. P., Wenker, T. N., & El-Serag, H. B. (2023). Global burden of gastric cancer: epidemiological trends, risk factors, screening and prevention. Nature reviews Clinical oncology, 20(5), 338-349. :

“In older men (≥65 years of age) intestinal-type gastric cancer is more common than the diffuse type, whereas patients with diffuse-type gastric cancer are more likely to be younger (<70 years of age, 50.9% versus 33.7% for intestinaltype gastric cancer) and/or female (46.6% versus 35.4% for intestinal-type gastric cancer)”

This variation likely reflects differences in study populations, sample sizes, and geographic regions. However, the key point that emerges consistently from previous studies is that diffuse‑type gastric cancer presents at a significantly younger age than intestinal‑type gastric cancer. The distinction is even more pronounced for hereditary and early‑onset diffuse subtypes. Hereditary diffuse gastric cancer (HDGC) has a well‑documented average onset of 38 years (range: 14–69 years).

Given the consistent evidence that DGC, particularly its early‑onset and hereditary forms, affects a significantly younger demographic, we selected samples from patients under 40 years of age to specifically capture the molecular features of young‑onset DGC. We have added the following statement to the Methods section to clarify this rationale:

We have added the following text to the **Materials and Methods section (Subsection 2.1. Data Acquisition and Preprocessing Page 7; Line 124-133)** to provide context on DGC incidence by age:

Early‑onset gastric cancer (≤45 years) more frequently presents as diffuse-type lesions arising in histologically normal mucosa, suggesting a greater role for genetic alterations compared to conventional GC (60–80 years), which is more strongly influenced by environmental factors (29). Given that diffuse‑type gastric cancer consistently presents at a younger age than intestinal‑type gastric cancer, and to specifically investigate the molecular drivers of young‑onset DGC, we restricted our inclusion criteria to patients under 40 years of age. We performed a systematic search of the Gene Expression Omnibus (GEO) database (https://www.ncbi.nlm.nih.gov/) and selected all available samples that met the following criteria: first; all samples were under 40 years old, second; the cancerous tissues were diffuse type, and third; all samples were paired-end sequenced. Ultimately, 12 samples (6 normal and 6 tumor) from two datasets (GSE113255, GSE122401) were identified and selected for this pilot analysis (S1 Table). The mean age of patients with available age data in our cohort (n=9 out of 12 samples) was 30.9 years (range: 25–35 years; SD: ±3.4 years).

**Table 1**. Cohort description of the exploratory RNA-seq analysis in young-onset diffuse gastric cancer. This table documents the most important data for the 12-sample pilot cohort of 6 DGC tumors and 6 normal tissues from datasets GSE113255 and GSE122401. N/A: Not aviable in GEO database.

| No. | GSE ID | GSM ID | SRA ID | Sample Type | Histology | Biospecimen type | Age | Sex | Library  Layout |
| --- | --- | --- | --- | --- | --- | --- | --- | --- | --- |
| 1 | GSE113255 | GSM3101070 | SRR7014300 | Normal | Normal mucosae | Tissue | N/A | N/A | Paired‐end |
| 2 | GSE113255 | GSM3101072 | SRR7014302 | Normal | Normal mucosae | Tissue | N/A | N/A | Paired‐end |
| 3 | GSE113255 | GSM3101068 | SRR7014298 | Normal | Normal mucosae | Tissue | N/A | N/A | Paired‐end |
| 4 | GSE122401 | GSM3501782 | SRR8281317 | Normal | Normal mucosae | Tissue | 33 | Male | Paired‐end |
| 5 | GSE122401 | GSM3501783 | SRR8281318 | Normal | Normal mucosae | Tissue | 29 | Male | Paired‐end |
| 6 | GSE122401 | GSM3501784 | SRR8281319 | Normal | Normal mucosae | Tissue | 30 | Female | Paired‐end |
| 7 | GSE113255 | GSM3101104 | SRR7014334 | Tumor | Diffuse Type | Tissue | 34 | Male | Paired‐end |
| 8 | GSE113255 | GSM3101117 | SRR7014347 | Tumor | Diffuse Type | Tissue | 25 | Female | Paired‐end |
| 9 | GSE113255 | GSM3101139 | SRR7014369 | Tumor | Diffuse Type | Tissue | 35 | Male | Paired‐end |
| 10 | GSE122401 | GSM3501862 | SRR8281397 | Tumor | Diffuse Type | Tissue | 33 | Male | Paired‐end |
| 11 | GSE122401 | GSM3501863 | SRR8281398 | Tumor | Diffuse Type | Tissue | 29 | Male | Paired‐end |
| 12 | GSE122401 | GSM3501864 | SRR8281399 | Tumor | Diffuse Type | Tissue | 30 | Female | Paired‐end |

GSE113255: https://www.ncbi.nlm.nih.gov/geo/query/acc.cgi?acc=GSE113255

GSE122401: https://www.ncbi.nlm.nih.gov/geo/query/acc.cgi?acc=GSE122401

We believe these revisions fully address the reviewer's concern by: (1) replacing the imprecise statement with accurate, evidence‑based language; (2) providing global mean age data for DGC and intestinal‑type GC from multiple independent studies; (3) providing the mean and SD for our own cohort; and (4) clearly justifying our focus on young‑onset patients under 40 years of age.

=============================================================================

**Comment 4.22 (Figure 1):** Figure 1 and associated text in materials and methods: the data was not collected (as this typically refers to new data generation), but rather it was identified from a public repository and reanalyzed. Please modify wording.

Also in Figure 1, “double-end” should be modified to “paired-end”

Figure 1 is a useful figure to provide a high-level view of the approach. However, details are lacking on how DEGs are determined, what samples or other inputs are used (depending on the method described), etc.

**Response**:

We have revised Figure 1 accordingly: (1) 'Data collection' has been replaced with 'Data acquisition from NCBI GEO repository'; (2) 'double-end' has been corrected to 'paired-end'; and (3) we have added detailed information on inputs, outputs, and key parameters for each analytical step, including the statistical thresholds (Log₂FC > 1, p-value < 0.05), feature selection criteria (top 5% of 21,542 genes), PPI confidence score (> 0.7), and miRNA database triangulation (≥2 databases). We believe these revisions provide a comprehensive yet clear overview of our integrated bioinformatics pipeline.

**Fig 1. Comprehensive workflow of the bioinformatics analysis for identifying and validating hub genes in diffuse gastric cancer (DGC).** The schematic outlines the sequential process from RNA‑seq data acquisition to multi‑omics validation. RNA‑seq data were acquired from the GEO public repository (GSE113255 and GSE122401), comprising 12 samples (6 normal and 6 DGC tissues) from patients under 40 years of age with paired‑end sequencing. After quality control, trimming, and alignment to the GRCh38 reference genome using CLC Genomics Workbench v20.0, differential expression analysis was performed using Baggerly's test with thresholds of |Log₂FC| > 1 and p-value < 0.05. Feature selection was conducted using the ReliefF algorithm on all 21,542 genes (k=10, 100 iterations), retaining the top 5% (n=1,260) for downstream analyses. Functional enrichment was performed using DAVID and SR plot (GO, KEGG; p-value < 0.05). Protein‑protein interaction networks were constructed via STRING (confidence > 0.7) and visualized in Cytoscape, with hub genes identified using the MCC algorithm (CytoHubba; top 7 nodes). Validation was conducted using GEPIA2 (TCGA‑STAD and GTEx) with Wilcoxon rank‑sum test and Log‑rank test (p < 0.05). Additional analyses included co‑expression networks (GeneMANIA; Pearson > 0.4) , immune infiltration (TIMER3.0; 5 immune cell types, TME scores) , miRNA regulation (TargetScan, miRDB, miRTarBase; high‑confidence: ≥2 databases), transcription factor prediction (TRRUST; adjusted p-value < 0.05) , and drug repurposing (DGIdb; FDA‑approved compounds, antagonistic/inhibitory mechanisms prioritized).

=============================================================================

**Comment 4.23 (Code deposition):** It would be preferable to submit the code to GitHub or another general repository (e.g., Zenodo or Figshare) so that it would be findable and more directly usable by other bioinformaticians for reproducibility.

**Response**:

The complete Python code for ReliefF analysis has been deposited in a public GitHub repository: <https://github.com/sjsajadi/UncoveringtheMolecularLandscapeofYoungOnsetDiffuseGastric.git>.

The code is also included as S2 Table in the supplementary files.

=============================================================================

**Comment 4.24 (RStudio version):** The version of RStudio used (2023) is quite old, is there a reason for this?

**Response**:

We thank the reviewer for this observation. In this specific section, RStudio 2023.12.1 was used solely as an IDE (Integrated Development Environment) for generating violin and PCA plots. This analysis relied entirely on the R version 4.3.2 and specific visualization packages (e.g., ggplot2), not on the IDE version itself. Importantly, the version of RStudio does not affect the statistical outputs or biological interpretations, as the computations are performed by the underlying R engine and package versions. It is worth noting that our core feature selection and machine learning analyses were conducted in Python (scikit-learn) , where the RStudio version is irrelevant. At the time of our analysis, RStudio 2023.12.1 was the current stable release, and we maintained this version to ensure full reproducibility of our visualization pipeline.

As noted in the other literature, RStudio versions such as 2023.09.0 are still actively used in published bioinformatics studies. For example:

Pérez-Díaz, C., Echeverría, R., Pérez-Carrascosa, F. M., Salcedo-Bellido, I., Requena, P., Barrios-Rodríguez, R., ... & Arrebola, J. P. (2025). Associations of internal persistent organic pollutant levels with sex hormones: An analysis by sex and menopausal status in a Spanish cohort. Iscience, 28(7).

We added the version of R in the manuscript: “R version 4.3.2 within the RStudio environment (2023.12.1)”

**Materials and Methods; Subsection 2.1. Data Acquisition and Preprocessing; Page 7; Lines 134-141**:

To assess and mitigate potential batch effects when combining datasets GSE113255 and GSE122401, we performed Principal Component Analysis (PCA) on the normalized expression matrix of all 12 samples (6 normal and 6 tumor) using the prcomp function in R version 4.3.2 (within the RStudio environment 2023.12.1) with scaling and centering (30). Visualization was performed using the ggplot2 package to confirm the absence of significant batch-related clustering prior to downstream differential expression analysis.

**Materials and Methods; Subsection 2.6. Validation of Hub Genes' Expression and Prognostic Significance; Page 11; Lines 224-226**:

To visually confirm the differential expression of the hub genes within our own dataset, violin plots comparing their expression levels in DGC tumors versus normal tissues were generated using R version 4.3.2 within the RStudio environment (2023.12.1).

=============================================================================

**Comment 4.25 (Figure 2 – colors and axis labeling):** Figure 2: the colors are very difficult to distinguish, hence the plots are hard to read and derive meaning. For ease, the x-axis for Fig 2D should denote that this is the -log(p-value) (log10?).

**Response**:

We have completely revised the figure to address both the color distinguishability and axis labeling concerns.

We have replaced the CNET plots with Sankey‑dot plots, which provide a more comprehensive and visually intuitive representation of the relationships between genes and enriched functional terms. The Sankey diagram clearly illustrates the flow of gene‑to‑term associations, while the adjacent dot plot simultaneously displays the statistical significance (–log₁₀(p‑value)) and gene count (dot size) for each enriched term. This combined visualization enhances readability and allows readers to easily interpret both the connections and the enrichment significance in a single integrated figure. All figures related to GO and KEGG results are moved to Supplementary files.

**S2 Fig. Gene Ontology (GO) enrichment analysis of Biological Processes (BP) for up- and down-regulated genes in diffuse gastric cancer.** The integrated Sankey–dot plot illustrates the relationship between significantly enriched BP terms and their associated differentially expressed genes. (A) Enriched BP terms for up-regulated genes. (B) Enriched BP terms for down-regulated genes. The Sankey diagram (left) connects each GO term to the corresponding genes, with node width proportional to the number of genes annotated to each term. The dot plot (right) visualizes the statistical significance (–log₁₀(p-value)) and gene count (dot size) for each enriched term. Only the significant terms (p-value < 0.05) are displayed.

**S3 Fig. Gene Ontology (GO) enrichment analysis of Molecular Functions (MF) for up- and down-regulated genes in diffuse gastric cancer.** The combined Sankey–dot plot depicts the functional annotations of differentially expressed genes based on their molecular activities. (A) MF terms associated with up-regulated genes. (B) MF terms associated with down-regulated genes. The Sankey diagram (left) connects each GO term to the corresponding genes, with node width proportional to the number of genes annotated to each term. The dot plot (right) visualizes the statistical significance (–log₁₀(p-value)) and gene count (dot size) for each enriched term. Only the significant terms (p-value < 0.05) are displayed.

**S4 Fig. Gene Ontology (GO) enrichment analysis of Cellular Components (CC) for up- and down-regulated genes in diffuse gastric cancer.** The Sankey–dot plot provides a comprehensive overview of the subcellular localization of differentially expressed gene products. (A) CC terms enriched among up-regulated genes. (B) CC terms enriched among down-regulated genes. The Sankey diagram (left) connects each GO term to the corresponding genes, with node width proportional to the number of genes annotated to each term. The dot plot (right) visualizes the statistical significance (–log₁₀(p-value)) and gene count (dot size) for each enriched term. Only the significant terms (p-value < 0.05) are displayed.

=============================================================================

**Comment 4.26 (Tables and figures to supplementary):** Some of the tables and figures from the main text could be moved to the supplementary. For example, Table 1 and the functional analyses of the differentially expressed genes could be moved to the supplementary since they either describe the study design further or provide results that are less emphasized (compared to those from the ReliefF-based network hubs).

**Response**:

We thank the reviewer for this valuable suggestion. In response, we have carefully reviewed all tables and figures and have moved the following items to the Supplementary Materials to streamline the main text and focus on the key findings derived from the ReliefF‑based network hub analysis:

**Figures:**

Fig 1. Comprehensive workflow of the bioinformatics analysis for identifying and validating hub genes in diffuse gastric cancer (DGC).

Fig 2. Identification of hub genes using the Maximal Clique Centrality (MCC) algorithm in Cytoscape.

Fig 3. Violin plots comparing Hub gene expression distributions between diffuse gastric cancer (DGC) tissues and normal samples.

Fig 4. Expression validation of the seven network-derived hub genes in gastric cancer.

Fig 5. Prognostic value of hub genes in gastric cancer.

Fig 6. Co-expression network and functional enrichment of hub genes in gastric cancer.

Fig 7. Correlation of hub genes with immune cell infiltration in gastric cancer.

Fig 8. A comprehensive regulatory and drug-gene interaction network for diffuse gastric cancer (DGC).

Fig 9. Mechanism of Bevacizumab-mediated suppression of the VEGF-A signaling pathway and its downstream effects on metastatic gene expression.

Fig 10. Master transcriptional regulators of the diffuse gastric cancer hub gene network.

**Tables:**

Table 1. Comparative analysis of hub gene expression from the ReliefF-based feature selection algorithm and the CLC Genomics Workbench v20.0 differential expression pipeline.

Table 2. Correlation analysis between hub gene expression and tumor microenvironment characteristics in gastric cancer estimated via TIMER3.0.

Table 3. miRNA regulators of hub genes identified through integrated bioinformatic analysis.

Table 4. Clinically approved drugs predicted to interact with hub genes in gastric cancer, as identified by a computational drug repurposing analysis.

Table 5. Regulatory network analysis of key transcription factors and their target hub genes in diffuse gastric cancer.

**Supporting information captions:**

S1 Fig. Principal Component Analysis (PCA) of the combined RNA-seq datasets GSE113255 and GSE122401.

S2 Fig. Gene Ontology (GO) enrichment analysis of Biological Processes (BP) for up- and down-regulated genes in diffuse gastric cancer.

S3 Fig. Gene Ontology (GO) enrichment analysis of Molecular Functions (MF) for up- and down-regulated genes in diffuse gastric cancer.

S4 Fig. Gene Ontology (GO) enrichment analysis of Cellular Components (CC) for up- and down-regulated genes in diffuse gastric cancer.

S5 Fig. KEGG pathway enrichment analysis of up- and down-regulated genes in diffuse gastric cancer.

S6 Fig. Gene Ontology (GO) enrichment analysis of the identified hub genes in diffuse gastric cancer.

S1 Table. Cohort description of the exploratory RNA-seq analysis in young-onset diffuse gastric cancer.

S2 Table. Python code for ReliefF feature selection analysis

S3 Table. The top 50 most informative upregulated and downregulated genes identified by the RReliefF feature selection algorithm.

**S4 Table. CLC Genomics Workbench analysis results**

**S5 Table. ReliefF-based feature selection analysis results**

**S6 Table. GO and KEGG enrichment analysis results of DEGs and hub genes
S7 Table. Immune cell infiltration analysis of hub genes
S8 Table. miRNA regulatory network analysis results of hub genes
S9 Table. Drug-gene interaction analysis results**

=============================================================================

**Comment 4.27:** Figure panels (e.g., A-D) should be combined into 1 for ease of reading.

**Response**:

In response, we have reorganized the figure panels to improve readability and visual coherence. Specifically:

BP (Biological Process) panels for Upregulated and Downregulated genes have been combined into a single figure (Supplementary Figure 2).

MF (Molecular Function) panels for Upregulated and Downregulated genes have been combined into a single figure (Supplementary Figure 3).

CC (Cellular Component) panels for Upregulated and Downregulated genes have been combined into a single figure (Supplementary Figure 4).

BP, CC, and MF panels for Hub genes have been combined into a single figure (Supplementary Figure 6).

These revisions ensure that related data are presented together, facilitating side‑by‑side comparison and improving the overall flow of the figures. We believe these changes significantly enhance the clarity and ease of interpretation for readers.

Given the number of hub genes (n=7), displaying all panels in a single figure would result in excessive panel density, potentially reducing clarity. Therefore, we have grouped the panels based on the biological functions of the hub genes:

Panel A (ECM‑related and inflammatory genes): CCL5, CXCR4, MMP9, and IL18: these genes are primarily associated with cell migration, extracellular matrix remodeling, and inflammatory responses.

Panel B (Immune regulatory genes): FOXP3, TNFSF11, and TNFSF13B : these genes play key roles in immune suppression, T‑cell differentiation, and tumor microenvironment modulation.

Accordingly:

Figure 3A and 3B (Violin plots)

Figure 4A and 4B (Box plots)

Figure 5A and 5B (Survival curves)

=============================================================================

**Comment 4.28 (Figure 7 – y-axis labels):** Figure 7: It would be useful to add y-axis labels that indicate the meaning of the values (e.g., log expression values, normalized or not?). In the legend of this figure, it could be useful to directly mention what expression values are represented in the y-axis (and how normalized), rather than “The plots illustrate the probability density of expression levels at different values”.

**Response**:

The violin plots display raw expression values (read counts) obtained directly from the RNA‑seq analysis pipeline. The y‑axis represents 'Expression (Raw Read Counts)' , and no normalization or log‑transformation was applied to these data, as the plots were intended to visually confirm the differential expression patterns within our own dataset. We have revised the figure legend to explicitly state this, replacing the vague description with a clear explanation of the data shown. All statistical analyses (e.g., differential expression, Fold Change) were performed using RPKM‑normalized data, as described in the Methods section.

We also revised the Figure legened:

**Fig 3. Violin plots comparing Hub gene expression distributions between diffuse gastric cancer (DGC) tissues and normal samples.** The y‑axis represents 'Expression (Raw Read Counts)', and the x‑axis indicates the sample groups (Tumor vs. Normal). Central lines within the plots denote the median expression, while the box boundaries indicate the interquartile range (IQR). This visualization highlights significant differences in expression profiles, including variations in distribution shape, spread, and central tendency, underscoring transcriptomic alterations in DGC relative to normal tissue. (A) *CCL5*, (B) *CXCR4*, (C) *MMP9*, (D) *IL18*, (E) *FOXP3*, (F) *TNFSF11*, and (G) *TNFSF13B*.

=============================================================================

We believe that the revised manuscript has been substantially improved in response to all reviewer comments. We thank the Academic Editor and all reviewers for their time and constructive feedback. We look forward to your favorable decision.

Respectfully submitted,

Matia Sadat Borhani (Corresponding author)

On behalf of all co-authors
