# Supplementary material for: Uncovering the molecular landscape of young-onset diffuse gastric cancer: A relieff-based feature selection analysis on RNA-Seq data
Source: PLoS One. 2026 Aug 6;21(8):e0354961. doi: 10.1371/journal.pone.0354961 (PMC13446702; doi:10.1371/journal.pone.0354961)
Supplement: Supplementary file 9 [file pone.0354961.s016.docx]

The core idea of this article is to identify several key transcription factors and central genes that play important roles in the pathogenesis of diffuse gastric cancer (DGC) by analyzing the transcription factor central gene interactions in DGC. CXCR4, MMP9, and TNFSF13B were identified as central genes in the study, and NFKB1 and RELA were found to be the most important transcription factors. These genes and transcription factors promote tumor proliferation and invasion by activating purine signaling pathways and enhancing the immunosuppressive tumor microenvironment. In addition, the study also explored the role of these genes in tumor immune escape and growth, and proposed the VEGF inhibitor Bevacizumab as a potential strategy for treating DGC. The research results lay the foundation for future validation studies with larger sample sizes, which will help develop more accurate diagnostic and treatment strategies.
