# Supplementary material for: Uncovering the molecular landscape of young-onset diffuse gastric cancer: A relieff-based feature selection analysis on RNA-Seq data
Source: PLoS One. 2026 Aug 6;21(8):e0354961. doi: 10.1371/journal.pone.0354961 (PMC13446702; doi:10.1371/journal.pone.0354961)
Supplement: Supplementary file 10 [file pone.0354961.s018.docx]

Overall assessment: After carefully reading the fully revised manuscript and all point-by-point revision responses addressing prior editorial and peer reviewer suggestions, I am satisfied with the comprehensive improvements made by the authors. The manuscript now presents a rigorous, transparent, well-structured bioinformatics pilot study focused on young-onset diffuse gastric cancer (DGC) utilizing ReliefF machine learning feature selection on RNA-seq data, and I support its acceptance for publication in PLOS ONE subject to no further major revisions. Detailed comments are as follows:

1. Manuscript completeness and standardization are significantly improved

The authors fully followed PLOS ONE formatting guidelines, rearranged all figure legends to the end of the text, standardized software, database versions and citation formats throughout the Methods section, supplemented critical cohort clinical information in Supplementary Table S1, and added PCA analysis to rule out cross-dataset batch effects, which effectively resolved multiple methodological clarity issues raised previously. All supplementary materials (Python code deposited on GitHub, complete analysis tables, high-resolution 600 dpi figures) are fully provided and accessible, meeting the journal’s open data and code sharing requirements.

2. Methodological ambiguities have been thoroughly clarified

The authors revised the ambiguous description of ReliefF screening logic, clearly distinguishing between "top 5% genes ranked by ReliefF discrimination weight" and simple average expression filtering; corrected code numerical typos and removed redundant train-test split residual codes inappropriate for the small sample cohort; explicitly stated the limitation that PPI network inference relies on transcript levels rather than protein abundance; and explained the rationality of retaining all 12 samples for feature selection given the rarity of young-onset DGC cases. The comparison between ReliefF multivariate screening and traditional univariate differential analysis (MMP9 high fold change but non-significant p-value) is now logically elaborated, which well highlights the advantages of machine learning in mining coordinated gene interactions ignored by conventional statistics.

3. Scientific narrative and logical consistency are optimized

The authors uniformly adopted the standardized term "three prioritized core hub genes" to differentiate the three key therapeutic targets (CXCR4, MMP9, TNFSF13B) from the seven hub genes derived from initial PPI network screening, eliminating textual confusion in Abstract, Highlights, Results and Discussion sections. The discussion section added explicit statements on the inherent limitations of the small pilot sample (n=6 per group) and the necessity of subsequent in vitro/in vivo experimental verification and large independent cohort validation, objectively positioning this work as a hypothesis-generating exploratory study without overstating clinical conclusions, greatly enhancing the objectivity of the paper.

4. Core research conclusions remain robust and valuable

The core scientific outputs of the paper are intact and strengthened after revision: seven hub genes driving young-onset DGC were identified, with CXCR4 confirmed as an independent poor prognostic biomarker (HR=1.5, p<0.01); NFKB1/RELA serve as master transcription factors governing the hub gene regulatory network; multi-layer omics analyses (immune infiltration, miRNA regulatory network, drug repurposing) systematically elucidate the metastasis, immune evasion and metabolic reprogramming mechanisms of DGC. The repurposing potential of bevacizumab, belimumab and denosumab is clearly supported by computational evidence, providing actionable therapeutic clues for precision treatment of young-onset diffuse gastric cancer.

5. Minor suggestions (optional polishing only, no mandatory revision)

- Minor language polishing: Individual repeated sentence structures in the Discussion section can be streamlined for readability;
- Supplementary figures labels: Check a small number of subgraph label alignment issues for uniform visual presentation.

Final Recommendation

The manuscript has fully addressed all prior critical concerns regarding sample description, batch correction, methodological reporting, writing consistency and study limitation disclosure. The study provides a reliable integrative machine learning-transcriptome analysis pipeline for rare early-onset DGC, and offers promising prognostic biomarkers and repurposing drug candidates for follow-up experimental research. I recommend the manuscript be accepted for publication in PLOS ONE after minor language and figure formatting touch-ups.
