# Supplementary material for: Uncovering the molecular landscape of young-onset diffuse gastric cancer: A relieff-based feature selection analysis on RNA-Seq data
Source: PLoS One. 2026 Aug 6;21(8):e0354961. doi: 10.1371/journal.pone.0354961 (PMC13446702; doi:10.1371/journal.pone.0354961)
Supplement: Peer review history

# Peer review: Decision Letter 0

**DOI:** 10.1371/journal.pone.0354961.r001

**Authors:** Zhanzhan Li

6 May 2026

PONE-D-25-58282

Uncovering the Molecular Landscape of Young-Onset Diffuse Gastric Cancer: A ReliefF-Based Feature Selection Analysis on RNA-Seq Data

PLOS One

Dear Dr. Borhani,

Thank you for submitting your manuscript to PLOS ONE. After careful consideration, we feel that it has merit but does not fully meet PLOS ONE’s publication criteria as it currently stands. Therefore, we invite you to submit a revised version of the manuscript that addresses the points raised during the review process.

Please submit your revised manuscript by Jun 20 2026 11:59PM. If you will need more time than this to complete your revisions, please reply to this message or contact the journal office at plosone@plos.org. When you're ready to submit your revision, log on to https://www.editorialmanager.com/pone/ and select the 'Submissions Needing Revision' folder to locate your manuscript file.

Please include the following items when submitting your revised manuscript:

- A letter that responds to each point raised by the academic editor and reviewer(s). You should upload this letter as a separate file labeled 'Response to Reviewers'.
- A marked-up copy of your manuscript that highlights changes made to the original version. You should upload this as a separate file labeled 'Revised Manuscript with Track Changes'.
- An unmarked version of your revised paper without tracked changes. You should upload this as a separate file labeled 'Manuscript'.

If you would like to make changes to your financial disclosure, please include your updated statement in your cover letter. Guidelines for resubmitting your figure files are available below the reviewer comments at the end of this letter.

If applicable, we recommend that you deposit your laboratory protocols in protocols.io to enhance the reproducibility of your results. Protocols.io assigns your protocol its own identifier (DOI) so that it can be cited independently in the future. For instructions see: https://journals.plos.org/plosone/s/submission-guidelines#loc-laboratory-protocols. Additionally, PLOS ONE offers an option for publishing peer-reviewed Lab Protocol articles, which describe protocols hosted on protocols.io. Read more information on sharing protocols at https://plos.org/protocols?utm_medium=editorial-email&utm_source=authorletters&utm_campaign=protocols.

As the corresponding author, your ORCID iD is verified in the submission system and will appear in the published article. PLOS supports the use of ORCID, and we encourage all coauthors to register for an ORCID iD and use it as well. Please encourage your coauthors to verify their ORCID iD within the submission system before final acceptance, as unverified ORCID iDs will not appear in the published article. *Only*  the individual author can complete the verification step; PLOS staff *cannot*  verify ORCID iDs on behalf of authors.

We look forward to receiving your revised manuscript.

Kind regards,

Zhanzhan Li

Academic Editor

PLOS One

**Journal Requirements:**

When submitting your revision, we need you to address these additional requirements.

1. Please ensure that your manuscript meets PLOS ONE's style requirements, including those for file naming. The PLOS ONE style templates can be found at

https://journals.plos.org/plosone/s/file?id=wjVg/PLOSOne_formatting_sample_main_body.pdf and

https://journals.plos.org/plosone/s/file?id=ba62/PLOSOne_formatting_sample_title_authors_affiliations.pdf

2. Please note that PLOS One has specific guidelines on code sharing for submissions in which author-generated code underpins the findings in the manuscript. In these cases, we expect all author-generated code to be made available without restrictions upon publication of the work. Please review our guidelines at https://journals.plos.org/plosone/s/materials-and-software-sharing#loc-sharing-code and ensure that your code is shared in a way that follows best practice and facilitates reproducibility and reuse.

3. Please ensure that you refer to Figure 14 in your text as, if accepted, production will need this reference to link the reader to the figure.

4. Please upload a new copy of Figures 6D, 8A-8G, 9A-9G, 10, 11, 13, and 14 as the detail is not clear. Please follow the link for more information:  https://journals.plos.org/plosone/s/figures

5. If the reviewer comments include a recommendation to cite specific previously published works, please review and evaluate these publications to determine whether they are relevant and should be cited. There is no requirement to cite these works unless the editor has indicated otherwise.

[Note: HTML markup is below. Please do not edit.]

Reviewers' comments:

Reviewer's Responses to Questions

**Comments to the Author**

1. Is the manuscript technically sound, and do the data support the conclusions?

The manuscript must describe a technically sound piece of scientific research with data that supports the conclusions. Experiments must have been conducted rigorously, with appropriate controls, replication, and sample sizes. The conclusions must be drawn appropriately based on the data presented.

Reviewer #1: Yes

Reviewer #2: No

Reviewer #3: Yes

Reviewer #4: Partly

**********

2. Has the statistical analysis been performed appropriately and rigorously?

Reviewer #1: Yes

Reviewer #2: No

Reviewer #3: Yes

Reviewer #4: No

**********

3. Have the authors made all data underlying the findings in their manuscript fully available?

The PLOS Data policy requires authors to make all data underlying the findings described in their manuscript fully available without restriction, with rare exception (please refer to the Data Availability Statement in the manuscript PDF file). The data should be provided as part of the manuscript or its supporting information, or deposited to a public repository. For example, in addition to summary statistics, the data points behind means, medians and variance measures should be available. If there are restrictions on publicly sharing data—e.g. participant privacy or use of data from a third party—those must be specified.

Reviewer #1: Yes

Reviewer #2: Yes

Reviewer #3: Yes

Reviewer #4: Yes

**********

4. Is the manuscript presented in an intelligible fashion and written in standard English?

PLOS ONE does not copyedit accepted manuscripts, so the language in submitted articles must be clear, correct, and unambiguous. Any typographical or grammatical errors should be corrected at revision, so please note any specific errors here.

Reviewer #1: Yes

Reviewer #2: No

Reviewer #3: Yes

Reviewer #4: No

**********

5. Review Comments to the Author

Please use the space provided to explain your answers to the questions above. You may also include additional comments for the author, including concerns about dual publication, research ethics, or publication ethics. (Please upload your review as an attachment if it exceeds 20,000 characters)

Reviewer #1: Overall Recommendation: Minor Revisions

Summary

This manuscript presents a compelling pilot study that applies a machine learning (ML) feature selection approach (ReliefF algorithm) to RNA-seq data from a rare cohort of young-onset diffuse gastric cancer (DGC) patients. The study identifies a network of seven hub genes and subsequently focuses on three central players—CXCR4, MMP9, and TNFSF13B—based on their statistical significance, profound overexpression, and complementary functional roles. The authors employ a comprehensive multi-omics validation strategy, including prognostic analysis, immune microenvironment assessment, and drug repositioning, to build a convincing case for the biological and clinical relevance of these hub genes. The work is novel, well-structured, and addresses an important unmet need in oncology. The methodology is sound for a pilot investigation, and the conclusions are supported by the data. The manuscript is suitable for publication after addressing the minor points outlined below.

Major Strengths

1. Novelty and Importance: The focus on young-onset DGC, an aggressive and understudied subtype, is a significant strength. The application of an ML-based feature selection method (ReliefF) to overcome the limitations of conventional statistics in a small sample size context is innovative and well-justified.

2. Comprehensive Analysis: The study goes beyond simple differential expression. The integrative pipeline, encompassing PPI network analysis, functional enrichment, immune infiltration, miRNA/TF regulation, and drug-gene interaction analysis, provides a holistic view of the molecular landscape.

3. Clinical Translational Potential: The identification of CXCR4 as a high-risk prognostic biomarker (HR=1.5, p<0.01) and the drug repositioning analysis suggesting Bevacizumab as an indirect therapeutic strategy are highly valuable findings with clear clinical implications. The highlighting of TNFSF13B as a novel immunomodulatory target is particularly insightful.

Comments for the Authors

The following suggestions are offered to further strengthen the manuscript prior to publication. These are considered minor revisions.

1. Clarification on Sample Size and Pilot Nature:

The authors correctly and transparently frame this as a pilot study. To further preempt potential reviewer concerns, it would be beneficial to briefly expand the discussion on the inherent limitations of the small sample size (n=6 per group). A sentence or two could explicitly discuss how future studies with larger, independent cohorts are essential to validate these findings and to move from hypothesis-generation to a definitive predictive model.

2. Statistical Interpretation:

The handling of the MMP9 result is appropriate (acknowledging the high fold-change despite non-significant p-value). To enhance clarity, consider adding a brief statement in the results or discussion explicitly attributing this discrepancy to the pilot-scale sample size and its impact on statistical power, which the ReliefF algorithm is designed to mitigate. This reinforces the methodological choice.

3. Terminology Consistency:

There is a minor inconsistency in the abstract and highlights regarding the number of "central players." The abstract mentions "three central players—CXCR4, MMP9, and TNFSF13B," while the first highlight states "a core hub gene network (CXCR4, MMP9, TNFSF13B)." However, the introduction lists seven hub genes. This is not incorrect but could be slightly confusing. Consider using a consistent term such as "three prioritized core hub genes" or "three central hub genes" to distinguish them from the initial seven identified through network analysis.

Conclusion

This is a robust and thoughtfully executed pilot study that makes a valuable contribution to the field of gastric cancer research. The integrative approach effectively bridges ML and network biology, providing a strong foundation for future research into DGC. The manuscript is well-written, and the findings are of interest to the readership of PLOS ONE. I recommend acceptance after the minor revisions suggested above have been addressed.

Reviewer #2: The authors analyzed publicly available RNA-seq data from diffuse gastric cancer using machine learning-based feature selection to prioritize hub genes. They identified CXCR4, MMP9, and TNFSF13B as a core hub gene network with the potential to drive diffuse gastric cancer. They also found that the VEGF inhibitor Bevacizumab indirectly targets CXCR4 and MMP9. Additionally, they proposed TNFSF13B as a novel and highly significant immunomodulatory hub gene.

All analyses are based on public sequencing datasets, and no experiments were conducted to validate their hypotheses. While bioinformatics data-mining approaches are useful and acceptable, the hypotheses generated should be further tested and verified through in vitro and in vivo studies. Without such validation, the analysis lacks meaningful support.

The manuscript is poorly organized. The figure legends should be placed at the end of the manuscript.

Reviewer #3: The core idea of this article is to identify several key transcription factors and central genes that play important roles in the pathogenesis of diffuse gastric cancer (DGC) by analyzing the transcription factor central gene interactions in DGC. CXCR4, MMP9, and TNFSF13B were identified as central genes in the study, and NFKB1 and RELA were found to be the most important transcription factors. These genes and transcription factors promote tumor proliferation and invasion by activating purine signaling pathways and enhancing the immunosuppressive tumor microenvironment. In addition, the study also explored the role of these genes in tumor immune escape and growth, and proposed the VEGF inhibitor Bevacizumab as a potential strategy for treating DGC. The research results lay the foundation for future validation studies with larger sample sizes, which will help develop more accurate diagnostic and treatment strategies.

Reviewer #4: Authors address an important gap in understanding the molecular disruptions that occur in diffuse gastric cancer and aim to define driver genes and their function to propose potential therapeutic interventions. Generally, the multi-layered approach of leveraging various types of annotation on protein-protein interactions, tumor micro-environment, survival, miRNA regulation and other methods is viable. Given the small number of sample sizes, authors aimed to provide depth into the potential functional impact of genes relevant to DGC by leveraging public knowledge sources. However, the clarity of the methods and approach used is insufficient to reproduce the results, and some results/conclusions are contradictory and overstated.

Major comments:

- Further information about the materials and methods should be included such as:

o Clear description of all samples that are used in the study, including age, sex, and biospecimen type (e.g., blood, tissue, etc.) at the minimum.

- Methods:

o Authors are combining samples from two different studies. Have they evaluated the feasibility of this? Are there possible batch effects that should be considered? Are control samples showing similar profiles? Similarly, authors combined cohort and GTEx normal tissue samples for survival analyses. How were these samples combined? How many samples are are utilized? Are the normal tissue appropriate for comparison against the STAD cohort (e.g., demographic matching)?

o References and versions are missing in the methods for tools that are used (e.g., CLC Genomic Workbench, FastQC, STRING, EPIC method, miRNA databases, etc).

o Focusing the analyses on only the top 5% most expressed genes is quite restrictive (lines 177-178). Are these top 5% based on mean expression only? Or differential expression, with or without statistical significance? From text later in the manuscript, the assumption is that these 1,260 are derived from ReliefF but then the statement on lines 177-178 is incorrect and confusing. Please clarify.

o Feature selection with ReliefF, mRMR, and F Test (Supplementary materials):

• The code indicates that authors split the data into training and testing with an 80:20 split. Did authors repeat that training/testing and evaluate the stability of the ReliefF weights, as well as the mRMR, and F test feature selection? While the authors are limited by the sample size, the stability (and thus ability to replicate) of the results could be demonstrated a bit further.

• The code indicates that “num_features=1262”. How was this determined? (Also for clarity, it may be best to remove that “num_features=1382” from the function definitions as those are overwritten with 1262 during the function calls.)

• What is the meaning of the function diff() in the pseudocode of Supp File 1?

o For the PPI network, were all interaction types (e.g., predicted and experimentally validated) utilized? What was the date of access?

o Authors use TPM in some analyses (e.g., tumor immune microenvironment) but it seems RPKM with others. Is this correct? If so, an explanation of why different data are used as input would be helpful.

- Results:

o Line 245 states that 1,260 genes were selected by ReliefF, yet the methods state that the 1,260 are genes with the highest average expression across all samples (line 178). Did the 1,260 genes result from differential analyses?

o Authors are measuring the transcriptome yet are using significant genes to draw PPI networks. While this can be useful, the limitation is the assumption that all genes are translated into functional proteins. This limitation should be stated.

o Line 299: authors state that results from ReliefF were “cross-validated against the differential expression results obtained from the CLC Genomics Workbench 20.0 pipeline.” What does this mean? Section 2.2 in the methods that describe the CLC Genomics Workbench does not describe any differential analysis. (Also, “CLC Genomics” and “CLC Genomic” are used, please be consistent).

o In Table 3, the use of Baggerley’s test is not explained nor cited in the Methods. The assumption is that those metrics are outputs from CLC but this needs clarity. This table would also benefit from showing the actual ReliefF weights. As is, there is no direct comparative analysis as is indicated in the title of the table. The normalized expression values shown could be moved to a supplementary material to focus the attention on the feature selection itself. Also, an FDR of 0.00 should be described with more precision (e.g., scientific notation can be used if needed).

- Authors make the point that the ReliefF method is more powerful than traditional differential expression methods (e.g., t-tests) because it considers relationships between genes. While multivariate methods are indeed complementary to univariate ones, authors state in lines 299-301 that “This comparative analysis confirmed a strong concordance between the two methods, with the selected hub genes showing significant and consistent expression patterns.” This statement contradicts results in Table 3 that clearly show that only 2 of the 7 hub genes identified with ReliefF are statistically significant in differential analyses.

- In Results, Line 271: a brief explanation of what CNET plots would help with understanding and readability.

- Throughout the results, new methods and approaches are introduced that are not described in the methods. Examples include GEPIA and Baggerly’s test. Further, the language to describe the purpose of the analyses is inconsistent, making the readability of the work challenging, particularly for readers that may not be familiar with all the tools utilized. For example, the “drug-gene interaction of hub genes” section identifies potential repurposed drugs for DGC. Rather than labeling the section names by what the methods are doing, labeling section names by the impact of the methods (e.g., in this case drug repurposing), would provide much clarity throughout the manuscript.

o Also, please note that drug repurposing and drug repositioning are not synonymous and in this case, it seems that authors are attempting to perform drug repurposing. Please clarity.

- Discussion, lines 560-561: authors state that “Kaplan-Meier overall survival (OS) analysis further established CXCR4 as a significant high-risk biomarker”. In and of itself, results from a univariate K-M analysis is not going to conclude that the analyzed gene is a significant biomarker. A biomarker requires validation that is not provided in this work.

- No limitations are provided except the small sample size.

Minor comments:

- Abstract: this is a bit of a stylistic preference but expressions like “profound overexpression” are imprecise and could be rendered more impactful with more granular detail (e.g., two-fold, three-fold?). The additional granularity is also relevant for efforts that mine abstracts with AI.

o Another example is in line 114: “extreme rarity of this patient population”. This statement is inflammatory and does not account for ultra-rare diseases for which there is an N of 1. Please revise. It would also be appropriate to provide the incidence of DGC by age, since the focus of this manuscript is on patients that are young.

- P.4, line 76: authors write “presents in older individuals (16-82 years)”. Could authors provide the mean and SD of age in DGC globally and in the samples that are being analyzed? The range is so broad that the numbers do not fit the word “older”.

- Figure 1 and associated text in materials and methods: the data was not collected (as this typically refers to new data generation), but rather it was identified from a public repository and reanalyzed. Please modify wording.

o Also in Figure 1, “double-end” should be modified to “paired-end”

o Figure 1 is a useful figure to provide a high-level view of the approach. However, details are lacking on how DEGs are determined, what samples or other inputs are used (depending on the method described), etc.

- It would be preferable to submit the code to GitHub or another general repository (e.g., Zenodo or Figshare) so that it would be findable and more directly usable by other bioinformaticians for reproducibility.

- The version of RStudio used (2023) is quite old, is there a reason for this?

- Figure 2: the colors are very difficult to distinguish, hence the plots are hard to read and derive meaning. For ease, the x-axis for Fig 2D should denote that this is the -log(p-value) (log10?).

- Some of the tables and figures from the main text could be moved to the supplementary. For example, Table 1 and the functional analyses of the differentially expressed genes could be moved to the supplementary since they either describe the study design further or provide results that are less emphasized (compared to those from the ReliefF-based network hubs).

- Figure panels (e.g., A-D) should be combined into 1 for ease of reading.

- Figure 7: It would be useful to add y-axis labels that indicate the meaning of the values (e.g., log expression values, normalized or not?). In the legend of this figure, it could be useful to directly mention what expression values are represented in the y-axis (and how normalized), rather than “The plots illustrate the probability density of expression levels at different values”.

**********

6. PLOS authors have the option to publish the peer review history of their article (what does this mean?). If published, this will include your full peer review and any attached files.

If you choose “no”, your identity will remain anonymous but your review may still be made public.

**Do you want your identity to be public for this peer review?**  For information about this choice, including consent withdrawal, please see our Privacy Policy.

Reviewer #1: No

Reviewer #2: No

Reviewer #3: **Yes:** Jian Liu

Reviewer #4: No

**********

[NOTE: If reviewer comments were submitted as an attachment file, they will be attached to this email and accessible via the submission site. Please log into your account, locate the manuscript record, and check for the action link "View Attachments". If this link does not appear, there are no attachment files.]

To ensure your figures meet our technical requirements, please review our figure guidelines: https://journals.plos.org/plosone/s/figures

You may also use PLOS’s free figure tool, NAAS, to help you prepare publication quality figures: https://journals.plos.org/plosone/s/figures#loc-tools-for-figure-preparation.

NAAS will assess whether your figures meet our technical requirements by comparing each figure against our figure specifications.

Attachment Submitted filename: Reviewer Attachments PONE-D-25-58282.docx

# Author comment: Author response to Decision Letter 1

**DOI:** 10.1371/journal.pone.0354961.r002

7 Jul 2026

Manuscript ID: PONE-D-25-58282

Uncovering the Molecular Landscape of Young-Onset Diffuse Gastric Cancer: A ReliefF-Based Feature Selection Analysis on RNA-Seq Data

Dear Dr. Zhanzhan Li (Academic Editor) and Reviewers,

We thank you for the opportunity to revise our manuscript. We are grateful to the reviewers for their thoughtful, rigorous, and constructive comments, which have significantly improved the clarity, transparency, and scientific rigor of our work. Below, we provide a point-by-point response to all comments from the Academic Editor and each reviewer. All changes in the manuscript have been marked using track changes.

=============================================================================

Academic Editor – Journal Requirements

=============================================================================

Comment 1: Please ensure that your manuscript meets PLOS ONE's style requirements.

Response:

We have reformatted the entire manuscript according to the PLOS ONE templates. This includes proper file naming, reference formatting, Tables and figure placement. All figure legends have been moved to the end of the manuscript, immediately after the References section.

=============================================================================

Comment 2: Code sharing – author-generated code must be made available without restrictions.

Response:

The complete Python code for ReliefF analysis has been deposited in a public GitHub repository: https://github.com/sjsajadi/UncoveringtheMolecularLandscapeofYoungOnsetDiffuseGastric.git.

The code is also included as S2 Table in the supplementary files.

=============================================================================

Comment 3: Please ensure that you refer to Figure 14 in your text.

Response:

Thank you for pointing this out. The name of this figure is changed to Fig 10 according to the reviewers´ comment. We have corrected the citation in the manuscript: Results section (Page 25; Lines 447-454; Subsection 3.10. Prediction of transcription factors of Hub genes:

Transcriptional regulatory network analysis using the TRRUST database identified several key transcription factors (TFs) as central regulators of the DGC hub gene landscape (Fig 10, Table 5). The TFs NFKB1 and RELA emerged as the most statistically significant master regulators (FDR = 1.57e-09 and 1.43e-07, respectively), demonstrating a complex regulatory profile by predominantly activating hub genes including CCL5, CXCR4, FOXP3, IL18, and MMP9. Notably, both TFs also exhibited a dual role by repressing MMP9. Further analysis defined a cohesive regulatory module wherein hub genes like MMP9 and CXCR4 are co-regulated by a suite of TFs. This intricate network pinpoints NF-κB signaling and MMP9 regulation as critical, multifaceted nodes in DGC pathogenesis.

=============================================================================

Comment 4: Please upload a new copy of Figures 6D, 8A-8G, 9A-9G, 10, 11, 13, and 14 as the detail is not clear.

Response:

All figures have been regenerated at 600 dpi resolution with improved contrast, labeling, and legibility.

=============================================================================

Comment 5: Please review and evaluate any recommended citations from reviewers.

Response:

We have carefully reviewed all citations suggested by the reviewers. Those that were directly relevant to our methods or findings have been incorporated into the reference list. Those that were not directly relevant have been noted but not cited, as permitted by the editor's instructions.

=============================================================================

Reviewer 1

=============================================================================

We sincerely thank Reviewer 1 for the positive and constructive feedback. Your recognition of the novelty, comprehensive analysis, and clinical translational potential of our work is greatly appreciated.

=============================================================================

Comment 1.1- Clarification on Sample Size and Pilot Nature: The authors correctly and transparently frame this as a pilot study. To further preempt potential reviewer concerns, it would be beneficial to briefly expand the discussion on the inherent limitations of the small sample size (n=6 per group). A sentence or two could explicitly discuss how future studies with larger, independent cohorts are essential to validate these findings and to move from hypothesis-generation to a definitive predictive model.

Response:

We agree completely. In the revised manuscript, we have added a dedicated paragraph in the Discussion section (Page 34; lines 609-617):

While our pilot study successfully demonstrates the feasibility of integrating machine learning with transcriptomic data for DGC analysis, the small sample size (n=6 per group) imposes important limitations, and our findings should be considered hypothesis-generating rather than definitive. Therefore, we strongly encourage future studies with larger, independent cohorts to validate the candidate genes and pathways identified here and to transition from hypothesis generation to definitive predictive modeling. In addition, all findings presented here require independent validation through in vitro (e.g., qPCR, Western blot, functional assays) and in vivo studies before any clinical translation. We also acknowledge the absence of experimental validation. While our bioinformatic predictions are supported by multiple lines of evidence (literature, network analysis, and public database validation), definitive proof of causality requires targeted in vitro experiments such as gene knockdown/overexpression studies, migration/invasion assays, and immunohistochemical confirmation at the protein level.

=============================================================================

Comment 1.2 (Statistical interpretation): The handling of the MMP9 result is appropriate (acknowledging the high fold-change despite non-significant p-value). To enhance clarity, consider adding a brief statement in the results or discussion explicitly attributing this discrepancy to the pilot-scale sample size and its impact on statistical power, which the ReliefF algorithm is designed to mitigate. This reinforces the methodological choice.

Response:

Thank you for this excellent suggestion. We have added a clear sentence in the Discussion section (Page 30-31; Lines: 533-545). The new text reads:

MMP9, a zinc-dependent matrix metalloproteinase, plays a critical role in GC progression and metastasis by degrading extracellular matrix components, notably type IV collagen in basement membranes (103, 104). Its expression is influenced by factors such as H. pylori infection and specific promoter polymorphisms like the -1562C/T variant, which elevates its mRNA and protein levels (105). Functionally, inhibiting MMP9 has been shown to suppress distant metastasis in GC through an MMP- 9/PI3K/AKT/Snail-dependent pathway, underscoring its therapeutic relevance (106). In our analysis, MMP9 exhibited the most pronounced overexpression (fold change = 16.6) among all hub genes, yet this did not reach statistical significance (p = 0.34) (Table 1). This discrepancy between a large effect size and a non-significant p-value is a well-documented phenomenon, often attributed to factors such as limited sample size, technical variation, or tumor heterogeneity, rather than a lack of biological relevance. For example, the substantial fold change, consistent with extensive research linking MMP9 overexpression to advanced GC stage and poor prognosis, solidifies its position as a biologically pivotal node in DGC progression, warranting further investigation in larger cohorts (107, 108). The ReliefF algorithm, which captures multivariate relationships, prioritized MMP9 as a top feature despite the non-significant univariate p-value, highlighting the complementary value of machine learning approaches in small-sample transcriptomic studies.

============================================================================

Comment 1.3 (Terminology consistency): There is a minor inconsistency in the abstract and highlights regarding the number of "central players." The abstract mentions "three central players—CXCR4, MMP9, and TNFSF13B," while the first highlight states "a core hub gene network (CXCR4, MMP9, TNFSF13B)." However, the introduction lists seven hub genes. This is not incorrect but could be slightly confusing. Consider using a consistent term such as "three prioritized core hub genes" or "three central hub genes" to distinguish them from the initial seven identified through network analysis.

Response:

This has been corrected. Throughout the abstract and highlights, we now consistently use the phrase " three prioritized core hub genes" (CXCR4, MMP9, TNFSF13B) to clearly distinguish them from the initial set of seven hub genes identified through network analysis:

Abstract (Results;Page 2; line 34-36): Our ML driven approach identified seven hub genes central to DGC pathogenesis including CCL5, CXCR4, MMP9, FOXP3, IL18, TNFSF11, and TNFSF13B. Among these, three prioritized core hub genes (CXCR4, MMP9, and TNFSF13B) were selected based on statistically significant overexpression and complementary functional roles.

Highlights (Page 3; line 52-53; point 1): changed from "a core hub gene network" to "a prioritized core hub gene network (CXCR4, MMP9, TNFSF13B)":

1. Machine learning identifies a prioritized core hub gene network (CXCR4, MMP9, TNFSF13B) driving diffuse gastric cancer (DGC) pathogenesis, revealing key drivers of metastasis, immune evasion, and extracellular matrix remodeling.

Results (Page 16; Line 337; Subsection 3.3. Analysis of protein-protein interaction network and Hub gene modules): added clarifying sentence: Subsequent analysis of the PPI network via the cytoHubba plugin in Cytoscape identified the initial seven hub genes including, CCL5 (chemokine ligand 5), CXCR4 (C-X-C chemokine receptor type 4 or RANTES), .....

Results (Page 19; Line 380; Subsection 3.5. Validation and Prognostic Value of Hub Genes): We further assessed the prognostic significance of the initial seven hub genes by generating overall survival (OS) curves in GEPIA, ....

Results (Page 25; Line 449-451; Subsection 3.10. Prediction of transcription factors of Hub genes): The TFs NFKB1 and RELA emerged as the most statistically significant master regulators (FDR = 1.57e-09 and 1.43e-07, respectively), demonstrating a complex regulatory profile by predominantly activating hub genes including CCL5, CXCR4, FOXP3, IL18, and MMP9.

Discussion (Page 29; Line 510): Network analysis identified initial seven hub genes exhibiting high connectivity, CCL5, CXCR4, MMP9, FOXP3, IL18, TNFSF11, and TNFSF13B, ......

Discussion (Page 33; Line 589): The final selection of CXCR4, MMP9, and TNFSF13B as prioritized core hub genes was driven by their statistical significance, elevated expression profiles, and complementary functional roles in DGC pathogenesis.

=============================================================================

Reviewer 2:

Comment 2.1 (Lack of experimental validation): The authors analyzed publicly available RNA-seq data from diffuse gastric cancer using machine learning-based feature selection to prioritize hub genes. They identified CXCR4, MMP9, and TNFSF13B as a core hub gene network with the potential to drive diffuse gastric cancer. They also found that the VEGF inhibitor Bevacizumab indirectly targets CXCR4 and MMP9. Additionally, they proposed TNFSF13B as a novel and highly significant immunomodulatory hub gene. All analyses are based on public sequencing datasets, and no experiments were conducted to validate their hypotheses. While bioinformatics data-mining approaches are useful and acceptable, the hypotheses generated should be further tested and verified through in vitro and in vivo studies. Without such validation, the analysis lacks meaningful support.

Response:

We appreciate Reviewer 2's candid assessment. Bioinformatics analyses of public data are a legitimate and widely accepted first step in cancer research, especially for rare diseases like young-onset DGC where sample collection is extremely difficult. We believe our study provides a valuable resource for guiding future experimental work. Our manuscript is explicitly framed as a pilot bioinformatics study with the primary aim of hypothesis generation, not definitive validation. We have been transparent about this

from the Abstract (Subsection Conclusion; Page 2; Line 43): "This pilot study establishes a robust integrative framework..."),

through the Introduction (Page 6; Line 119-121): Ultimately, we seek to generate robust hypotheses about oncogenic pathways and candidate driver genes, thereby laying the groundwork for future validation studies with larger cohorts."),

to the Discussion (Page 34; Line 609-613): While our pilot study successfully demonstrates the feasibility of integrating machine learning with transcriptomic data for DGC analysis, the small sample size (n=6 per group) imposes important limitations, and our findings should be considered hypothesis-generating rather than definitive. Therefore, we strongly encourage future studies with larger, independent cohorts to validate the candidate genes and pathways identified here and to transition from hypothesis generation to definitive predictive modeling.

Also, in the Conclusion section (Page 34; Lines 623-626): Although definitive confirmation in a larger, independent cohort is an essential next step, this work provides a powerful methodological foundation and valuable insights. We believe this pilot study will be a valuable resource for guiding future research, and its findings are poised for significant improvement with increased sample sizes, paving the way for more precise diagnostic and therapeutic strategies in this aggressive cancer.).

Additionally, we have substantially strengthened the manuscript based on your comment.

In the Discussion (Page 34; Lines 613-617), we have added this paragraph: In addition, all findings presented here require independent validation through in vitro (e.g., qPCR, Western blot, functional assays) and in vivo studies before any clinical translation. We also acknowledge the absence of experimental validation. While our bioinformatic predictions are supported by multiple lines of evidence (literature, network analysis, and public database validation), definitive proof of causality requires targeted in vitro experiments such as gene knockdown/overexpression studies, migration/invasion assays, and immunohistochemical confirmation at the protein level.

=============================================================================

Comment 2.2 (Poor organization and figure legends): The manuscript is poorly organized. The figure legends should be placed at the end of the manuscript.

Response:

We have completely reorganized the manuscript according to PLOS ONE standards. All figure legends have been moved to the end of the manuscript, immediately following the References section.

=============================================================================

Reviewer 3:

The core idea of this article is to identify several key transcription factors and central genes that play important roles in the pathogenesis of diffuse gastric cancer (DGC) by analyzing the transcription factor central gene interactions in DGC.

CXCR4, MMP9, and TNFSF13B were identified as central genes in the study, and NFKB1 and RELA were found to be the most important transcription factors. These genes and transcription factors promote tumor proliferation and invasion by activating purine signaling pathways and enhancing the immunosuppressive tumor microenvironment.

In addition, the study also explored the role of these genes in tumor immune escape and growth, and proposed the VEGF inhibitor Bevacizumab as a potential strategy for treating DGC. The research results lay the foundation for future validation studies with larger sample sizes, which will help develop more accurate diagnostic and treatment strategies.

Response:

We greatly appreciate your positive evaluation of our work. Your summary accurately capture

Attachment Submitted filename: Response to Reviewers.docx

# Peer review: Decision Letter 1

**DOI:** 10.1371/journal.pone.0354961.r003

**Authors:** Zhanzhan Li

16 Jul 2026

Uncovering the Molecular Landscape of Young-Onset Diffuse Gastric Cancer: A ReliefF-Based Feature Selection Analysis on RNA-Seq Data

PONE-D-25-58282R1

Dear Dr. Matia Sadat Sadat Borhani,

We’re pleased to inform you that your manuscript has been judged scientifically suitable for publication and will be formally accepted for publication once it meets all outstanding technical requirements.

Within one week, you’ll receive an e-mail detailing the required amendments. When these have been addressed, you’ll receive a formal acceptance letter and your manuscript will be scheduled for publication.

An invoice will be generated when your article is formally accepted. Please note, if your institution has a publishing partnership with PLOS and your article meets the relevant criteria, all or part of your publication costs will be covered. Please make sure your user information is up-to-date by logging into Editorial Manager at Editorial Manager® and clicking the ‘Update My Information' link at the top of the page. For questions related to billing, please contact billing support.

If your institution or institutions have a press office, please notify them about your upcoming paper to help maximize its impact. If they’ll be preparing press materials, please inform our press team as soon as possible -- no later than 48 hours after receiving the formal acceptance. Your manuscript will remain under strict press embargo until 2 pm Eastern Time on the date of publication. For more information, please contact onepress@plos.org.

Kind regards,

Zhanzhan Li

Academic Editor

PLOS One

Additional Editor Comments (optional):

Reviewers' comments:

Reviewer's Responses to Questions

**Comments to the Author**

1. If the authors have adequately addressed your comments raised in a previous round of review and you feel that this manuscript is now acceptable for publication, you may indicate that here to bypass the “Comments to the Author” section, enter your conflict of interest statement in the “Confidential to Editor” section, and submit your "Accept" recommendation.

Reviewer #3: All comments have been addressed

**********

2. Is the manuscript technically sound, and do the data support the conclusions?

The manuscript must describe a technically sound piece of scientific research with data that supports the conclusions. Experiments must have been conducted rigorously, with appropriate controls, replication, and sample sizes. The conclusions must be drawn appropriately based on the data presented.

Reviewer #3: Yes

**********

3. Has the statistical analysis been performed appropriately and rigorously?

Reviewer #3: Yes

**********

4. Have the authors made all data underlying the findings in their manuscript fully available?

The PLOS Data policy requires authors to make all data underlying the findings described in their manuscript fully available without restriction, with rare exception (please refer to the Data Availability Statement in the manuscript PDF file). The data should be provided as part of the manuscript or its supporting information, or deposited to a public repository. For example, in addition to summary statistics, the data points behind means, medians and variance measures should be available. If there are restrictions on publicly sharing data—e.g. participant privacy or use of data from a third party—those must be specified.

Reviewer #3: Yes

**********

5. Is the manuscript presented in an intelligible fashion and written in standard English?

PLOS ONE does not copyedit accepted manuscripts, so the language in submitted articles must be clear, correct, and unambiguous. Any typographical or grammatical errors should be corrected at revision, so please note any specific errors here.

Reviewer #3: Yes

**********

6. Review Comments to the Author

Please use the space provided to explain your answers to the questions above. You may also include additional comments for the author, including concerns about dual publication, research ethics, or publication ethics. (Please upload your review as an attachment if it exceeds 20,000 characters)

Reviewer #3: Overall assessment: After carefully reading the fully revised manuscript and all point-by-point revision responses addressing prior editorial and peer reviewer suggestions, I am satisfied with the comprehensive improvements made by the authors. The manuscript now presents a rigorous, transparent, well-structured bioinformatics pilot study focused on young-onset diffuse gastric cancer (DGC) utilizing ReliefF machine learning feature selection on RNA-seq data, and I support its acceptance for publication in PLOS ONE subject to no further major revisions. Detailed comments are as follows:

Manuscript completeness and standardization are significantly improved

The authors fully followed PLOS ONE formatting guidelines, rearranged all figure legends to the end of the text, standardized software, database versions and citation formats throughout the Methods section, supplemented critical cohort clinical information in Supplementary Table S1, and added PCA analysis to rule out cross-dataset batch effects, which effectively resolved multiple methodological clarity issues raised previously. All supplementary materials (Python code deposited on GitHub, complete analysis tables, high-resolution 600 dpi figures) are fully provided and accessible, meeting the journal’s open data and code sharing requirements.

Methodological ambiguities have been thoroughly clarified

The authors revised the ambiguous description of ReliefF screening logic, clearly distinguishing between "top 5% genes ranked by ReliefF discrimination weight" and simple average expression filtering; corrected code numerical typos and removed redundant train-test split residual codes inappropriate for the small sample cohort; explicitly stated the limitation that PPI network inference relies on transcript levels rather than protein abundance; and explained the rationality of retaining all 12 samples for feature selection given the rarity of young-onset DGC cases. The comparison between ReliefF multivariate screening and traditional univariate differential analysis (MMP9 high fold change but non-significant p-value) is now logically elaborated, which well highlights the advantages of machine learning in mining coordinated gene interactions ignored by conventional statistics.

Scientific narrative and logical consistency are optimized

The authors uniformly adopted the standardized term "three prioritized core hub genes" to differentiate the three key therapeutic targets (CXCR4, MMP9, TNFSF13B) from the seven hub genes derived from initial PPI network screening, eliminating textual confusion in Abstract, Highlights, Results and Discussion sections. The discussion section added explicit statements on the inherent limitations of the small pilot sample (n=6 per group) and the necessity of subsequent in vitro/in vivo experimental verification and large independent cohort validation, objectively positioning this work as a hypothesis-generating exploratory study without overstating clinical conclusions, greatly enhancing the objectivity of the paper.

Core research conclusions remain robust and valuable

The core scientific outputs of the paper are intact and strengthened after revision: seven hub genes driving young-onset DGC were identified, with CXCR4 confirmed as an independent poor prognostic biomarker (HR=1.5, p<0.01); NFKB1/RELA serve as master transcription factors governing the hub gene regulatory network; multi-layer omics analyses (immune infiltration, miRNA regulatory network, drug repurposing) systematically elucidate the metastasis, immune evasion and metabolic reprogramming mechanisms of DGC. The repurposing potential of bevacizumab, belimumab and denosumab is clearly supported by computational evidence, providing actionable therapeutic clues for precision treatment of young-onset diffuse gastric cancer.

Minor suggestions (optional polishing only, no mandatory revision)

Minor language polishing: Individual repeated sentence structures in the Discussion section can be streamlined for readability;

Supplementary figures labels: Check a small number of subgraph label alignment issues for uniform visual presentation.

Final Recommendation

The manuscript has fully addressed all prior critical concerns regarding sample description, batch correction, methodological reporting, writing consistency and study limitation disclosure. The study provides a reliable integrative machine learning-transcriptome analysis pipeline for rare early-onset DGC, and offers promising prognostic biomarkers and repurposing drug candidates for follow-up experimental research. I recommend the manuscript be accepted for publication in PLOS ONE after minor language and figure formatting touch-ups.

**********

7. PLOS authors have the option to publish the peer review history of their article (what does this mean?). If published, this will include your full peer review and any attached files.

If you choose “no”, your identity will remain anonymous but your review may still be made public.

**Do you want your identity to be public for this peer review?**  For information about this choice, including consent withdrawal, please see our Privacy Policy.

Reviewer #3: **Yes:** Jian Liu

**********

Attachment Submitted filename: Reviewer 3 Revised Manuscript Comments.docx

# Editor report: Acceptance letter

**DOI:** 10.1371/journal.pone.0354961.r004

**Authors:** Zhanzhan Li

PONE-D-25-58282R1

PLOS One

Dear Dr. Borhani,

I'm pleased to inform you that your manuscript has been deemed suitable for publication in PLOS One. Congratulations! Your manuscript is now being handed over to our production team.

At this stage, our production department will prepare your paper for publication. This includes ensuring the following:

* All references, tables, and figures are properly cited

* All relevant supporting information is included in the manuscript submission,

* There are no issues that prevent the paper from being properly typeset

You will receive further instructions from the production team, including instructions on how to review your proof when it is ready. Please keep in mind that we are working through a large volume of accepted articles, so please give us a few days to review your paper and let you know the next and final steps.

Lastly, if your institution or institutions have a press office, please let them know about your upcoming paper now to help maximize its impact. If they'll be preparing press materials, please inform our press team within the next 48 hours. Your manuscript will remain under strict press embargo until 2 pm Eastern Time on the date of publication. For more information, please contact onepress@plos.org.

You will receive an invoice from PLOS for your publication fee after your manuscript has reached the completed accept phase. If you receive an email requesting payment before acceptance or for any other service, this may be a phishing scheme. Learn how to identify phishing emails and protect your accounts at https://explore.plos.org/phishing.

If we can help with anything else, please email us at customercare@plos.org.

Thank you for submitting your work to PLOS ONE and supporting open access.

Kind regards,

PLOS ONE Editorial Office Staff

on behalf of

Dr. Zhanzhan Li

Academic Editor

PLOS One
